# GNPS-assisted identification of bioactive constituents from *Scoparia dulcis* and their anti-inflammatory mechanisms *via* network pharmacology and experimental validation

**DOI:** 10.1039/d6ra06433g

**Published:** 2026-10-07

**Authors:** Ngoc-Thac Pham, Thanh-Sang Nguyen, Ngo Thi My Duyen, Huong-Giang Le, Thuy-Tien Thi Phan, Quoc Dung Huynh Tran, Phuong Vu Luu, Quang-Hien Kha, Vu Duc Loi, Duc-Toan Nguyen, Chung-Kuang Lu, Kuei-Hung Lai

**Affiliations:** a Department of Pharmacognosy and Traditional Pharmacy, School of Pharmacy, University of Medicine and Pharmacy at Ho Chi Minh City Ho Chi Minh City 700000 Vietnam; b PhD Program in Clinical Drug Development of Herbal Medicine, College of Pharmacy, Taipei Medical University Taipei 110301 Taiwan kueihunglai@tmu.edu.tw; c Food Technology Faculty, Saigon Technology University Ho Chi Minh City 700000 Vietnam; d Faculty of Pharmacy, Lac Hong University 10 Huynh Van Nghe Street, Tran Bien Ward Dong Nai City Vietnam; e Institute of Pharmaceutical Education and Research, Binh Duong University Ho Chi Minh City Vietnam; f Faculty of Pharmacy, Ton Duc Thang University Ho Chi Minh City Vietnam; g International PhD Program in Medicine, College of Medicine, Taipei Medical University Taipei 110301 Taiwan; h Vietnam University of Traditional Medicine 2 Tran Phu, Dai Mo Hanoi Vietnam; i University of Information Technology, Computer Science Ho Chi Minh City Vietnam; j National Research Institute of Chinese Medicine, Ministry of Health and Welfare Taipei 112026 Taiwan cklu@nricm.edu.tw; k Department of Life Sciences, Institute of Genome Sciences, National Yang Ming Chiao Tung University No. 155, Sec. 2, Li-Nong Street, Beitou Taipei 112304 Taiwan; l Traditional Herbal Medicine Research Center, Taipei Medical University Hospital Taipei 110301 Taiwan; m National Museum of Marine Biology and Aquarium Pingtung 944401 Taiwan; n Graduate Institute of Pharmacognosy, College of Pharmacy, Taipei Medical University Taipei 110301 Taiwan

## Abstract

*Scoparia dulcis* L. (SD) is an edible medicinal herb traditionally consumed as a cooling beverage in tropical Asia for the management of metabolic and inflammatory disorders; however, its dietary bioactive constituents and anti-inflammatory mechanisms have not yet been fully elucidated. In the present study, the metabolite profile of the aerial parts of SD (ASD) was characterized, and their anti-inflammatory potential was evaluated through an integrated approach incorporating LC-HRMS/MS-based molecular networking, network pharmacology, molecular docking, molecular dynamics simulations, and *in vitro* validation in macrophages. Untargeted LC-HRMS/MS combined with GNPS feature-based molecular networking enabled the annotation of 184 metabolites in the ASD extract, mainly comprising shikimates and phenylpropanoids (44.0%) and terpenoids (39.7%), with flavonoids constituting the most abundant superclass and including known food bioactives such as β-sitosterol, pentamethoxyflavone, naringin, and catechin. ADMET-based screening prioritized 31 absorbable constituents, while network pharmacology identified 179 inflammation-related targets, with TNF-α, IL6, STAT3, AKT1, and JAK2 emerging as key hubs. Enrichment analysis highlighted the TNF, PI3K–AKT, JAK–STAT, and AGE–RAGE pathways as major signaling routes associated with low-grade diet-related inflammation. Molecular docking and 300 ns molecular dynamics simulations further identified zeorinone as a stable dual binder of TNF-α and IL6, exhibiting the most favorable MM–PBSA binding free energies among the compounds selected for molecular dynamics analysis. Consistent with these computational findings, the ASD extract showed no cytotoxicity in RAW264.7 macrophages at 10–100 µg mL^−1^ and significantly reduced LPS-induced TNF-α and IL6 secretion at 50–100 µg mL^−1^, with effects comparable to dexamethasone. Overall, these findings establish SD as a promising source of dietary anti-inflammatory bioactives and provide a chemical and mechanistic basis for its potential development as a functional food ingredient or herbal beverage targeting chronic diet-related inflammation.

## Abstract 2

*Scoparia dulcis* L. is a medicinal herb traditionally consumed in tropical Asia for the management of metabolic and inflammatory disorders; however, its bioactive constituents and anti-inflammatory mechanisms have not yet been fully elucidated.

## Graphical abstract

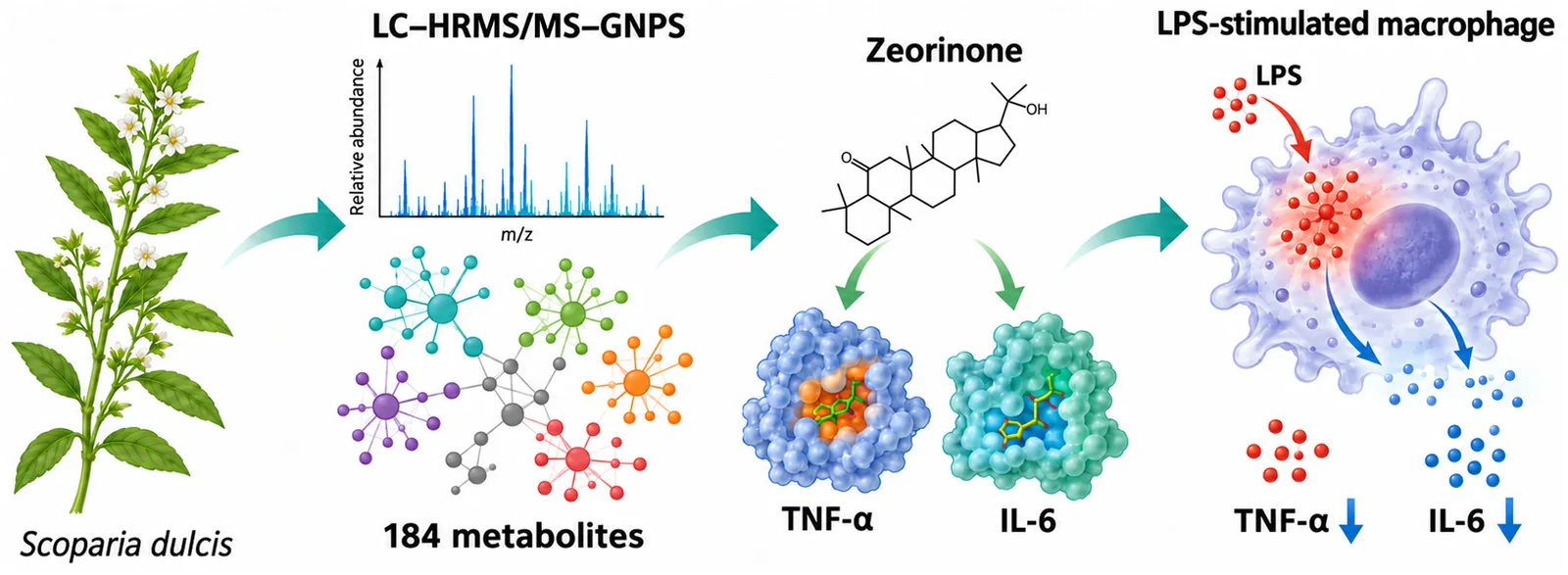

## Introduction

Inflammation is a fundamental host response to infection or tissue injury and, when properly resolved, is essential for wound healing and tissue homeostasis.^1^ However, when resolution is impaired, the same machinery sustains a state of chronic, low-grade systemic inflammation that is now widely recognised as a central driver of many diet-related non-communicable diseases, including type 2 diabetes, obesity, atherosclerosis, and metabolic syndrome.^2,3^ In the initial stage of inflammation, neutrophils and monocyte-derived macrophages are rapidly recruited to the site of injury, where they secrete pro-inflammatory mediators, including TNF-α, IL6, and IL-1β, thereby amplifying the local immune response. Under physiological conditions, this response is self-limiting through neutrophil apoptosis, efferocytosis, and a phenotypic switch of macrophages toward a pro-resolving, tissue-reparative state.^1^ When these checkpoints fail, sustained leukocyte infiltration, excessive generation of reactive oxygen species and inflammatory mediators, and inflammatory forms of cell death (necrosis, pyroptosis, ferroptosis, and NETosis) perpetuate tissue damage and accelerate disease progression, and aging itself contributes through the persistent low-grade phenotype known as inflammaging.^1,4^ Crucially, dietary patterns are now established as one of the strongest modifiable determinants of this inflammatory tone: Western-style diets rich in refined carbohydrates, saturated fats, and ultra-processed foods promote a pro-inflammatory phenotype, whereas diets rich in fruits, vegetables, whole grains, herbs, and herbal beverages are consistently associated with lower circulating concentrations of inflammatory biomarkers.^2,5^ While conventional anti-inflammatory drugs (*e.g.*, NSAIDs, corticosteroids, statins, metformin) remain widely used, their long-term consumption is constrained by gastrointestinal, renal, cardiovascular, and metabolic adverse effects.^6^ In this context, bioactive compounds derived from foods, especially polyphenols, flavonoids, and terpenoids, have gained increasing attention as safer and more sustainable approaches for modulating chronic inflammation by targeting key pathways such as NF-κB, MAPK signaling pathway, and cytokine networks.^6–8^ Edible herbs and traditional herbal infusions are a particularly attractive source, because they are already embedded in local food cultures, possess long records of safe human consumption, and combine sensory acceptance with measurable physiological benefits.^9,10^

*Scoparia dulcis* L. (SD; sweet broomweed, Plantaginaceae) is a perennial edible herb widely distributed across the tropical and subtropical regions of Asia, Africa, the Americas, and India.^11^ In Taiwan, southern China, and other parts of East and Southeast Asia, the aerial parts of SD have long been incorporated into “cooling” herbal beverages (*e.g.*, qīng-căo-chá/liáng chá) traditionally consumed to clear heat, soothe the throat, and support metabolic, hepatic, and gastrointestinal health.^10,12^ Phytochemical studies indicate that SD is a rich source of dietary relevant compounds, including flavonoids such as catechin, naringin, and apigenin derivatives, as well as phenolic acids, diterpenoids of the scopadulane series such as scopadulcic acids and scopadulciol, triterpenoids, steroids, and benzoxazinoids. Many of these compounds are well known for their antioxidant and anti-inflammatory activities in food related contexts.^11,13^ Beverages and aqueous extracts derived from SD have been reported to exert antidiabetic, hepatoprotective, antihyperlipidemic, antioxidant, anti-arthritic, and anti-atherosclerotic activities,^11,14^ collectively supporting its potential as a functional-food/beverage candidate for the dietary management of low-grade systemic inflammation associated with metabolic disorders. Despite this promising profile, a comprehensive metabolite-level characterisation of SD as a food matrix, and a mechanistic understanding of how its constituents engage the host inflammatory network, are still lacking. Untargeted mass-spectrometry-based metabolomics coupled with molecular networking has emerged as a powerful workflow for the chemical annotation of complex plant-derived food matrices, enabling rapid dereplication of known metabolites and the discovery of novel bioactives.^15^ Building on this rationale, the present study used Global Natural Products Social Molecular Networking (GNPS) to profile the chemical landscape of SD extracts,^15^ coupled with *in silico* ADMET filtering, target prediction, and network pharmacology to identify their potential molecular targets and inflammation-related signalling pathways.^16–19^ The anti-inflammatory activity of the crude extracts was further evaluated *in vitro*. The findings are intended to provide a chemical and functional basis for the development of SD-based food ingredients and functional beverages aimed at modulating diet-related chronic inflammation.

## Results and discussion

### Chemical landscape of ASD revealed by LC-HRMS/MS and FBMN

The currently reported chemical space of *Scoparia* spp., based on 385 natural products retrieved from the Reaxys database, is predominantly composed of terpenoids (54.03%), followed by shikimates and phenylpropanoids (32.73%), whereas alkaloids (5.71%), polyketides (4.16%), fatty acids (2.86%), and amino acids and peptides (0.52%) constitute comparatively minor fractions. Overall, this distribution indicates that the phytochemical profile of *Scoparia* spp. is largely dominated by terpenoid and phenolic-related metabolites, which appear to form the principal chemical framework of the genus and represent two of the most extensively reported classes of dietary bioactive compounds in edible plants and functional beverages (Fig. 1).

**Fig. 1:**
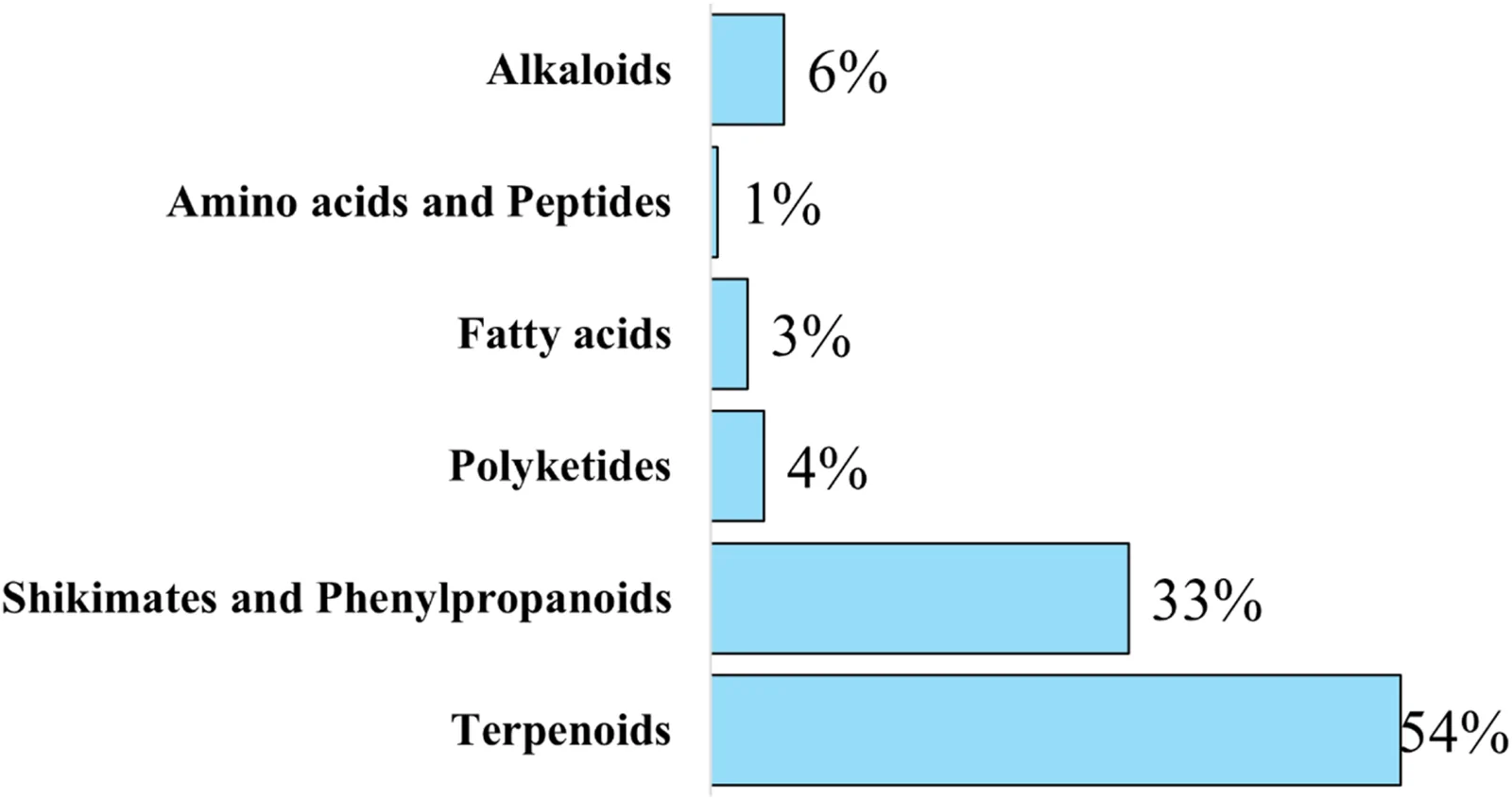
Natural products reported in *Scoparia* spp. (*n* = 385) retrieved from Reaxys.

From a chemical perspective, the strong predominance of terpenoids is consistent with the chemotaxonomic features of edible aromatic and medicinal-food plants, in which this class frequently contributes to flavor, antioxidant capacity, and anti-inflammatory activity, and is now widely exploited as natural food preservatives and functional-food ingredients.^20,21^ The substantial proportion of shikimates and phenylpropanoids further highlights the importance of aromatic and polyphenol-related metabolites in *Scoparia*, which may contribute to antioxidant, anti-inflammatory, and other health-promoting properties frequently associated with herbal teas and plant-based foods.^11^ Although minor classes such as alkaloids, polyketides, and fatty acids were present at lower proportions, their occurrence still broadens the overall metabolic diversity of *Scoparia* and may provide additional functional relevance. Overall, the Reaxys-derived distribution demonstrates that *Scoparia* spp. possesses a chemically rich but clearly biased metabolite repertoire, with terpenoids and shikimates/phenylpropanoids representing the major documented constituents and thus providing a useful phytochemical basis for subsequent metabolomic and bioactivity-oriented investigations of this edible herb.

The feature-based molecular networks of the ASD extract, acquired in both negative- and positive-ion modes, together with their corresponding chemical class distributions, are presented in Fig. 2. Overall, the positive-ion dataset afforded broader metabolite coverage than the negative-ion dataset, comprising 2350 total features, of which 348 were annotated, whereas the negative-ion dataset contained 1414 total features, including 212 identified features. Despite this difference in total feature count, the annotation rates of the two ionization modes were highly comparable, corresponding to approximately 14.8% in positive mode and 15.0% in negative mode. These findings indicate that positive-ion acquisition expanded the detectable metabolome primarily by increasing the total number of observed ions, rather than substantially enhancing the proportion of structurally annotated metabolites. In both molecular networks, the annotated square nodes were dispersed among a considerably larger number of circular unannotated nodes, underscoring the substantial chemical complexity of the extract as well as the still-limited coverage of current spectral libraries and *in silico* annotation tools for plant secondary metabolites.

**Fig. 2:**
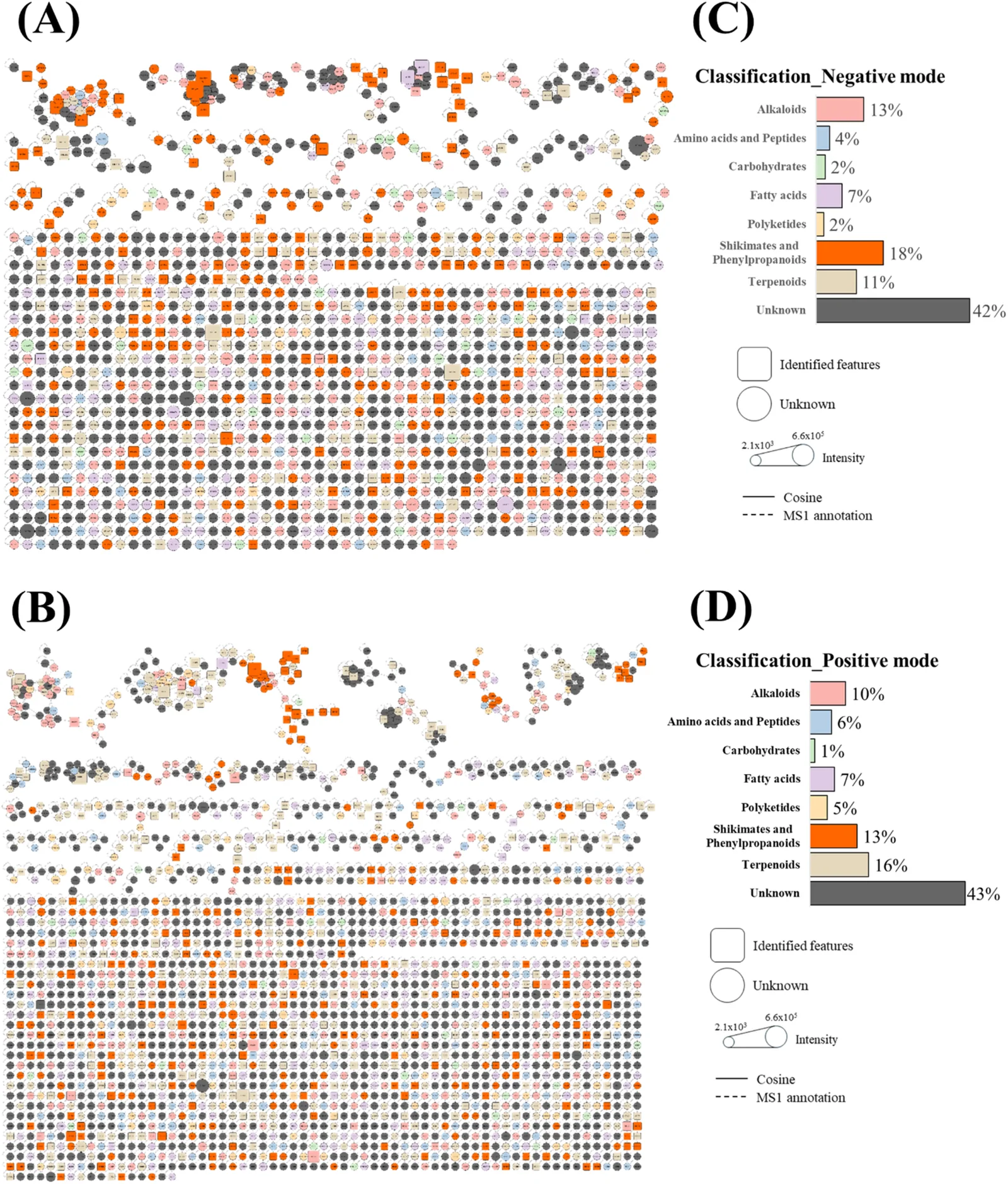
Feature-based molecular networks of the ASD extract acquired in (A) negative-ion mode and (B) positive-ion mode, together with the chemical class distribution of detected features in (C) negative-ion mode and (D) positive-ion mode. In the molecular networks, each node represents a detected LC-MS/MS feature, with node size proportional to signal intensity. Square nodes indicate annotated features, whereas circular nodes represent unknown features. Solid edges denote cosine-based MS/MS spectral similarity, while dashed edges indicate MS1 annotation relationships.

The class-distribution profiles further highlight the complementary nature of the two ionization modes. In the negative-ion dataset, unknown features represented the largest proportion (42%), followed by shikimates and phenylpropanoids (18%), alkaloids (13%), terpenoids (11%), fatty acids (7%), amino acids and peptides (4%), and minor proportions of carbohydrates and polyketides (2% each). In the positive-ion dataset, unknown features also predominated (42%), while shikimates and phenylpropanoids (15%), terpenoids (14%), and alkaloids (11%) remained the major annotated classes, followed by fatty acids (7%), amino acids and peptides (5%), polyketides (4%), and carbohydrates (2%). These patterns suggest that negative-ion mode was more effective for capturing relatively acidic and oxygenated metabolites, particularly phenolic and phenylpropanoid-related constituents, whereas positive-ion mode provided broader coverage of protonatable or adduct-forming compounds,^22,23^ thereby increasing the overall number of detected features.^24^ Taking together, the FBMN results demonstrate that the combined use of both ionization modes substantially broadened the observable chemical space of SD and provided a stronger foundation for metabolite annotation, molecular family organization, and subsequent bioactivity-oriented investigation.

As summarized in Table S1, metabolite annotation of the ASD extract revealed 184 characterized constituents, predominantly shikimates and phenylpropanoids (44.0%) and terpenoids (39.7%), whereas alkaloids, fatty acids, and polyketides were present in comparatively lower proportions. Flavonoids represented the most abundant superclass, and the complementary detection of compounds across both positive- and negative-ionization modes further confirmed that dual-mode LC-HRMS/MS improved the coverage of chemically diverse constituents. Importantly, the annotated metabolome encompassed several well-documented dietary bioactives with established roles in human nutrition and food science, including β-sitosterol, a major plant phytosterol broadly distributed in vegetables, legumes, nuts, and seeds, and previously shown to attenuate LPS-induced secretion of TNF-α, IL-6, and IL-1β in vascular endothelial cells;^22^ 5-hydroxy-6,7,8,3′,4′-pentamethoxyflavone, a polymethoxyflavone structurally related to citrus-derived nobiletin and tangeretin, with reported anti-inflammatory activity in cytokine-driven skin and immune models;^23^ and the dietary flavonoids catechin (a flavan-3-ol abundant in tea and cocoa) and naringin (a flavanone glycoside characteristic of citrus fruits), both previously reported in SD and widely studied for their antioxidant, antihyperglycemic, and anti-inflammatory effects.^8^ The extract also contained the *Scoparia*-characteristic diterpenoids scopadulcic acids and scopadulciol, together with the hopane-type triterpenoid zeorinone, further enriching the terpenoid fraction. Collectively, the FBMN-derived metabolome positions SD as a phenolic- and terpenoid-rich edible herb whose chemical inventory closely overlaps with bioactive classes traditionally associated with anti-inflammatory functional foods and beverages, providing a chemically rational basis for the network pharmacology and *in vitro* analyses described in the following sections.

### Anti-inflammatory activity of ASD extract in LPS-stimulated RAW 264.7 macrophages

To evaluate the anti-inflammatory potential of the chemically diverse, phenolic- and terpenoid-rich ASD extract identified above, the LPS-stimulated RAW 264.7 macrophage model was employed. The cytotoxic effects of the ASD extract on RAW 264.7 cells were evaluated using the MTT assay at concentrations of 10, 50, and 100 µg mL^−1^ (Fig. 3A). Following 24 h of exposure, RAW264.7 cell viability remained unchanged across all tested concentrations, suggesting that the ASD extract did not produce detectable cytotoxic effects under the conditions employed. Based on this result, the extract was further examined for its influence on inflammatory cytokine release. In LPS-activated RAW264.7 macrophages, ASD significantly altered the secretion of the pro-inflammatory mediators TNF-α and IL-6 in a concentration-dependent manner. In particular, treatment with 100 and 50 µg mL^−1^ markedly reduced TNF-α levels relative to the LPS-treated control, with the strongest suppression observed at 100 µg mL^−1^, approaching the effect of dexamethasone (Fig. 3B). In contrast, treatment at 10 µg mL^−1^ did not suppress TNF-α production and instead resulted in a significant increase compared with the LPS group, indicating that very low doses of the extract are insufficient to counteract LPS-induced cytokine release and may even potentiate it. Such hormetic, dose-dependent behaviour is well documented for polyphenol- and terpenoid-rich plant extracts, in which low doses can transiently promote redox- or NF-κB-mediated cytokine release, whereas higher doses overwhelm these pathways and exert net anti-inflammatory effects.^7,8^ A broadly similar but more pronounced concentration-dependent pattern was observed for IL6 (Fig. 3C). ASD at 50 and 100 µg mL^−1^ significantly attenuated IL6 secretion, and at 100 µg mL^−1^ IL6 levels were reduced to approximately half of those obtained with dexamethasone, indicating that the ASD extract was more effective than the steroidal reference control in suppressing LPS-induced IL6 release at this concentration. Although 10 µg mL^−1^ also produced a statistically significant reduction in IL6 levels relative to LPS alone, its effect was considerably weaker than those observed at the higher concentrations. The cytokine response at the lowest dose showed a different pattern, with a slight decrease in IL6 but an unexpected increase in TNF-α. This difference can be explained by the distinct regulatory mechanisms of these cytokines, as TNF-α is mainly controlled by NF-κB, while IL6 is regulated by both NF-κB and STAT3.^7,8,25,26^ These findings further support a hormetic effect, suggesting that the optimal active dose range of the extract is between 50 and 100 µg per mL.

**Fig. 3:**
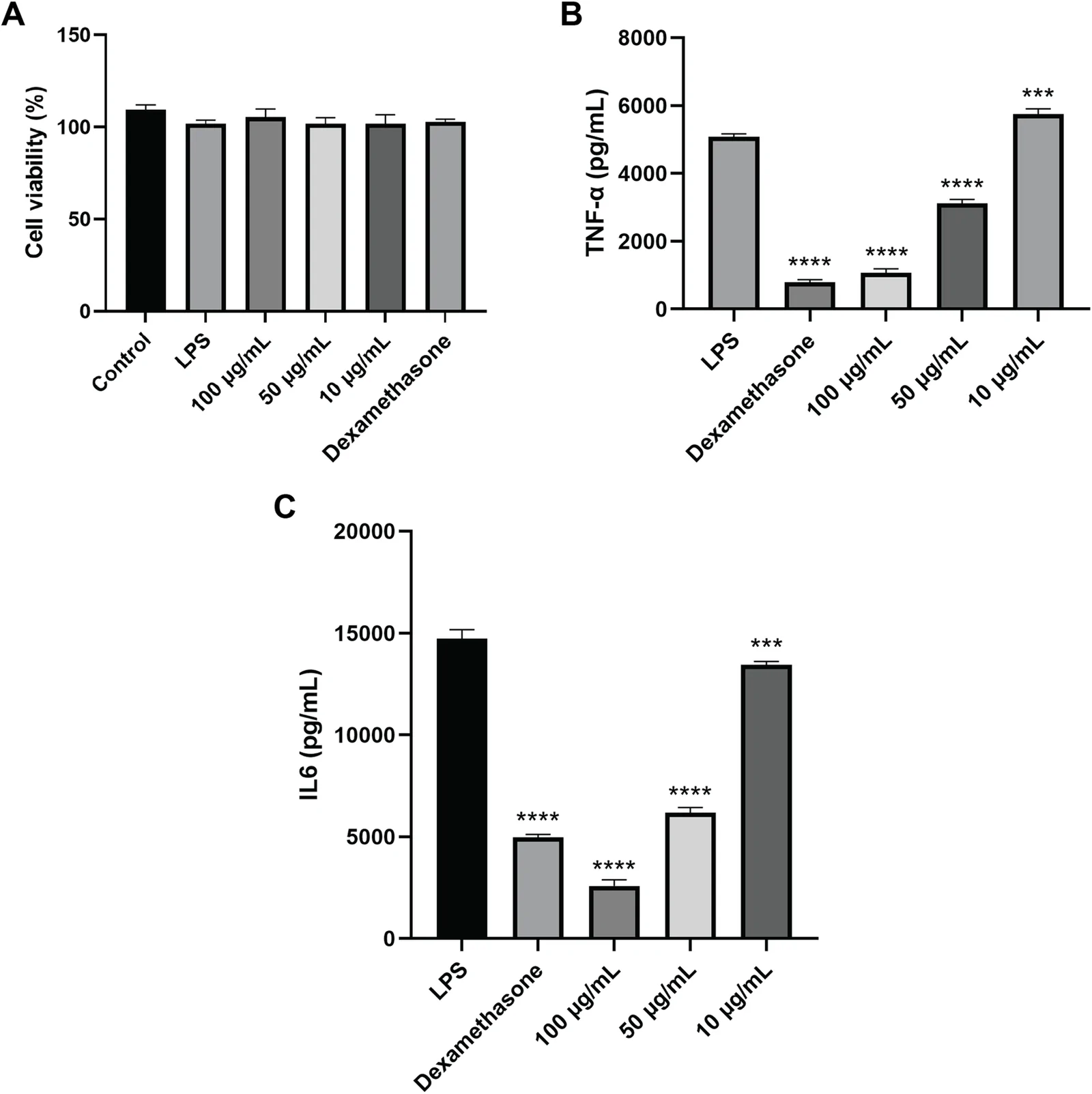
Cytotoxic effects of ASD extracts on RAW 264.7 cells (A), and their effects on TNF-α (B) and IL6 (C) production in LPS-stimulated RAW 264.7 cells. Dexamethasone (10 µM) was used as the reference control. Data are presented as the mean ± S.D. from three independent experiments performed in triplicate. *p < 0.05, **p < 0.01, and ***p < 0.001 *versus* the LPS-treated group.

### Network pharmacology links SD food bioactives to inflammation-related protein targets

#### PPI network analysis

To gain mechanistic insight into the cytokine-suppressive effects observed above, the annotated food-derived bioactive compounds were subjected to an integrated network pharmacology workflow incorporating ADMET-based screening, target prediction, and protein–protein interaction (PPI) analysis. LC-MS/MS analysis of the SD crude extract resulted in the identification of 184 chemical constituents. Subsequent pharmacokinetic profiling using ADMETlab 3.0 (Table S2) indicated that 31 compounds satisfied the predefined screening criteria, including Lipinski's rule of five, Caco-2 permeability, and oral bioavailability. These findings suggest that these compounds possess favorable properties for intestinal permeability and absorption, thereby supporting their potential as candidate bioactive agents for further investigation. Subsequent target prediction, integrating data from SwissTargetPrediction, identified 644 unique molecular targets. Comparative analysis using a Venn diagram (Fig. 4A) revealed 179 overlapping targets between these predicted targets and the consolidated set of inflammation-related genes (*n* = 1067) retrieved from the GeneCards, OMIM, and DisGeNET databases. These shared targets were then used to construct a protein–protein interaction (PPI) network, which comprised 169 nodes representing target proteins and 1122 edges representing protein–protein interactions, with an average node degree of 13.28, as shown in Fig. 4B. Topological analysis of this network identified ten hub proteins, namely TNF-α, IL6, STAT3, SRC, AKT1, EGFR, JAK2, BCL2, PIK3R1, and PIK3CA, as the most central nodes based on degree values (Table 1 and Fig. 4C), underscoring their likely importance in mediating the anti-inflammatory activity of ASD.

**Fig. 4:**
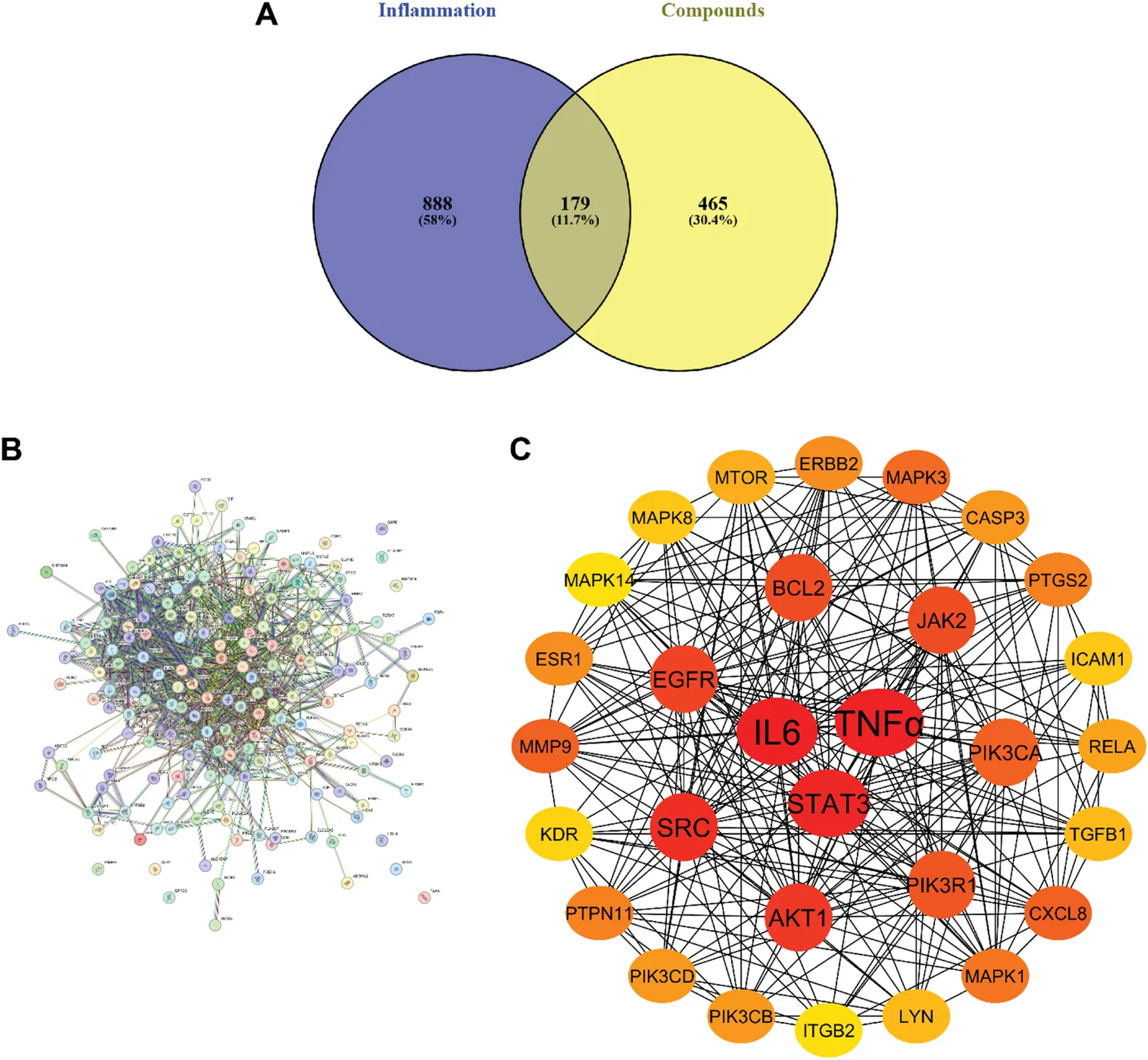
Comprehensive network pharmacology investigation of inflammation-related targets linked to ASD. (A) Venn diagram illustrating overlapping target genes among ASD and inflammation. (B) PPI network of shared targets constructed using the STRING database. (C) Top 30 core target genes identified *via* topological analysis based on node degree. Nodes represent targets associated with the identified compounds, with darker coloration and larger node size indicating stronger correlations.

**Table 1:** Key characteristics of the top 30 proteins within the PPI-network

Name	Degree	Betweenness centrality	Closeness centrality
TNF	64	0.122	0.571
IL6	64	0.130	0.573
STAT3	62	0.087	0.564
SRC	58	0.097	0.562
AKT1	51	0.051	0.542
EGFR	50	0.057	0.553
JAK2	39	0.025	0.491
BCL2	39	0.039	0.514
PIK3R1	38	0.024	0.458
PIK3CA	37	0.009	0.457
CXCL8	37	0.038	0.499
MMP9	37	0.039	0.508
MAPK3	35	0.022	0.508
MAPK1	34	0.017	0.511
PTGS2	33	0.103	0.512
PTPN11	33	0.008	0.469
ERBB2	29	0.029	0.488
ESR1	29	0.037	0.494
CASP3	28	0.013	0.484
PIK3CD	28	0.004	0.435
PIK3CB	28	0.004	0.435
RELA	27	0.011	0.490
MTOR	26	0.008	0.472
TGFB1	25	0.010	0.476
LYN	25	0.003	0.442
ICAM1	24	0.009	0.471
MAPK8	24	0.011	0.475
KDR	23	0.008	0.449
MAPK14	22	0.007	0.471
PTK2	22	0.005	0.442

#### GO enrichment and KEGG pathway analysis

Using ShinyGO v0.82, Gene Ontology (GO) enrichment analysis was performed on the top 50 inflammation-related genes associated with ASD, and the top 20 enriched terms were subsequently examined across the three GO categories, namely Biological Process (BP), Cellular Component (CC), and Molecular Function (MF) (Fig. 5A–C). Overall, the enrichment profiles indicate that the hub proteins are closely linked to inflammation-related functions. In the BP category, enriched terms such as regulation of immune system process, immune system process, response to external stimulus, response to stress, and cellular response to chemical stimulus suggest broad involvement in cytokine- and stress-mediated inflammatory signaling. The concurrent enrichment of protein phosphorylation, phosphorylation, and regulation of intracellular signal transduction further underscores the importance of kinase-dependent signaling cascades, consistent with the roles of JAK2, SRC, AKT1, EGFR, PIK3R1, and PIK3CA. The presence of apoptotic process and programmed cell death terms additionally suggests a role in inflammation-associated cell survival and death, with BCL2 and TNF likely contributing to these effects. In the CC category, enrichment of phosphatidylinositol 3-kinase complex class IA, phosphatidylinositol 3-kinase complex class I, plasma membrane signaling receptor complex, membrane raft, and membrane microdomain highlights the importance of membrane-associated signaling platforms, particularly those involving the PI3K/AKT axis. In the MF category, the predominance of terms related to protein tyrosine kinase activity, protein kinase activity, kinase binding, and ATP binding indicates that phosphorylation-dependent molecular functions are central to the enriched target set. Collectively, these findings suggest that the hub proteins regulate inflammation through interconnected immune signaling, membrane-associated receptor activity, kinase-driven intracellular transduction, and apoptosis-related mechanisms, with the TNF/IL6/JAK2/STAT3 and PI3K/AKT/EGFR/SRC axes emerging as major inflammatory networks.

**Fig. 5:**
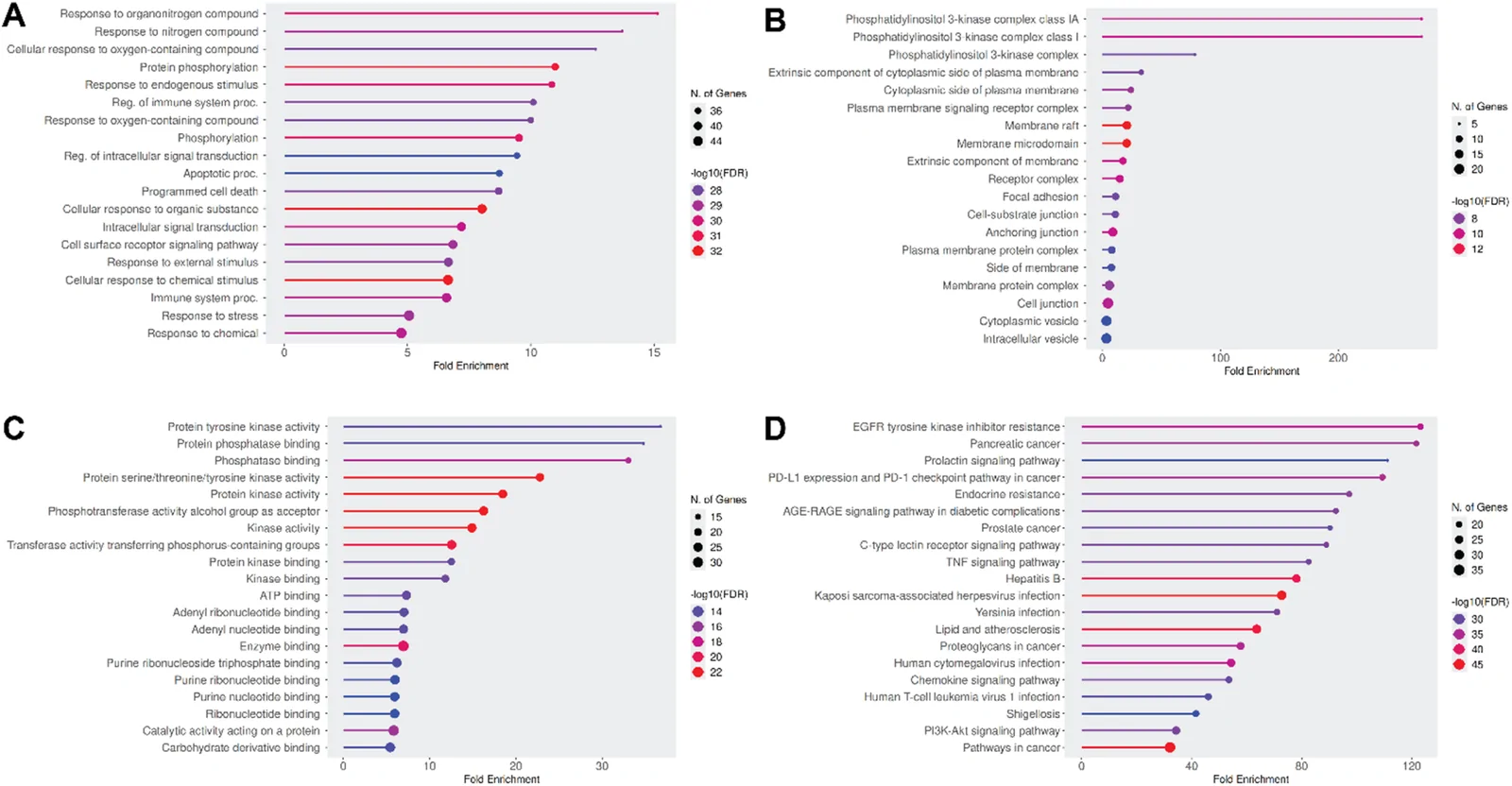
Gene Ontology (GO) enrichment analysis of the target genes was summarized across three domains: (A) Biological Process (BP), (B) Cellular Component (CC), and (C) Molecular Function (MF), together with (D) KEGG pathway enrichment. Bubble color intensity, ranging from red to blue, represents the −log_10_(FDR) value, with red indicating greater statistical significance and blue indicating lower significance. Bubble size corresponds to the number of genes enriched in each term or pathway.

The top 20 pathways identified by KEGG pathway enrichment analysis are presented in Fig. 5D. Overall, the enrichment profile suggests that the hub proteins are predominantly involved in inflammation-related signaling networks, indicating that inflammatory regulation is a major biological theme underlying the dataset. In particular, TNF and IL6 are closely associated with the TNF signaling pathway, chemokine signaling pathway, AGE–RAGE signaling pathway in diabetic complications, and several infection-related pathways, including hepatitis B, *Yersinia* infection, and Shigellosis, all of which are strongly linked to inflammatory and immune responses. JAK2 and STAT3 further reinforce this framework through their roles in cytokine-mediated intracellular signaling and their likely involvement in the prolactin signaling pathway, PD-L1 expression and PD-1 checkpoint pathway in cancer, and other immune-regulatory pathways.^25,26^ In addition, AKT1, PIK3R1, and PIK3CA may contribute to inflammation-associated responses through the PI3K–AKT signaling pathway, which interacts extensively with cytokine signaling and regulates inflammatory cell activation and survival. EGFR and SRC may also participate in inflammatory crosstalk by amplifying intracellular signaling cascades, whereas BCL2 may promote the survival of activated cells by inhibiting apoptosis under inflammatory conditions. The coordinated targeting of these interconnected pathways by structurally diverse ASD components, including phenolic compounds, flavonoids, sterols, and terpenoids, supports a multi component and multi target mode of action.^7,25–27^ This pattern is increasingly recognised as a key characteristic of dietary phytochemicals and functional foods. It also provides a mechanistic explanation for the dose dependent reduction in TNF-α and IL6 secretion observed in macrophages. Among the ten hub proteins identified, TNF-α and IL6 were selected as the main targets for subsequent docking and molecular dynamics analyses. This selection was based on their roles as the highest ranked hubs in the protein interaction network and as experimentally validated cytokine markers of the ASD extract.

### Molecular docking identifies key bioactive–target interactions

#### Docking protocol validation

To verify the reliability of the docking protocol, grid validation was performed by redocking the co-crystallized ligand into the binding site using the same grid parameters as those applied in the main docking study. The docked pose was then superimposed onto the original crystallographic conformation, and the root mean square deviation (RMSD) was calculated to assess the agreement between the two structures. As presented in Fig. S1, the redocking procedure was successfully achieved. In the case of TNF-α, the co-crystallized ligand yielded a docking score of −7.073 kcal mol^−1^, and the superimposed docked and native poses showed an RMSD of 0.565 Å, which is well below the commonly accepted threshold of 2.0 Å. Such a low RMSD value indicates a close correspondence between the predicted and experimental binding modes. These findings confirm that the generated grid accurately encompassed the inhibitor-binding region and support the validity of the docking strategy employed in this study.

#### Molecular docking

To evaluate the binding interactions between compounds identified in the ASD extract and the two principal target proteins, molecular docking simulations were performed using AutoDock Vina (Table S3). Most ligand–protein complexes exhibited predicted binding affinities more favorable than −5.0 kcal mol^−1^, whereas nine compounds against TNF-α and seven compounds against IL6 showed comparatively weaker predicted binding affinities. The five ligands with the most favorable docking scores for each target were subsequently selected for detailed interaction analysis. These ligands were found to occupy an overlapping region within the same binding site of their respective target proteins, as illustrated in Fig. S3 and S4. In addition, compound 307 were used as control ligands for comparison, based on their reported inhibitory activities against TNF-α.^28^

For TNF-α, two ligands, ASD171 (β-sitosterol) and ASD21 (zeorinone), displayed lower binding energies than the ligand 307 with docking scores of −7.546 and −7.093 kcal mol^−1^, respectively. Moreover, all of the top five ligands interacted with TNF-α through hydrophobic contact with TYR59, except ASD17, which interacted predominantly through TYR151, as presented in Table 2 and Fig. 6.

**Table 2:** Top five docking poses of ASD-derived compounds against TNF-α

Compound	Docking scores (kcal mol^−1^)	Interaction residue	
		Hydrogen bond	Other bonds
ASD171	−7.546		HIS15, TYR59
ASD21	−7.093		TYR59, TYR119
ASD68	−7.003	GLY121	TYR59
ASD 88	−6.39		TYR59, TYR119
ASD17	−6.242		TYR151
307^a^	−7.073	TYR151	GLY121, TYR59, LEU57, LEU120

a Control ligand.

**Fig. 6:**
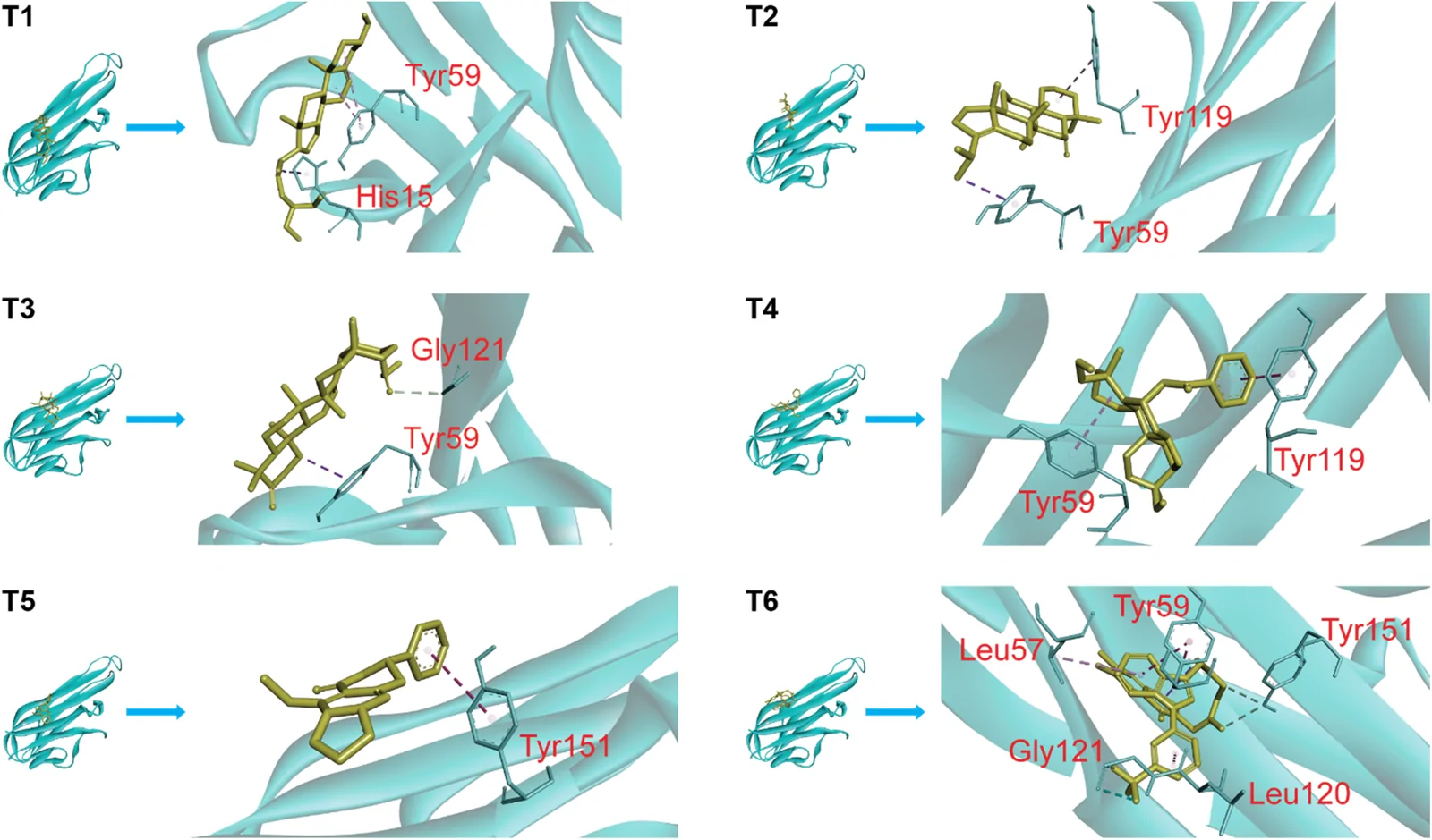
3D interaction between top 5 selected compounds and protein TNF-α. (T1) ASD171. (T2) ASD21. (T3) ASD68. (T4) ASD88. (T5) ASD17. (T6) 307.

For IL6, three ligands, ASD38 (5-hydroxy-6,7,8,3′,4′-pentamethoxyflavone), ASD21, and ASD179 (3,6-dimethoxy kaempferol), exhibited binding affinities lower than −7.0 kcal mol^−1^, with ASD38 showing the most favorable docking score (−7.843 kcal mol^−1^), as presented in Table 3 and Fig. 7. Specifically, ASD38 formed a hydrogen bond with GLN75 and a π–π stacking interaction with PHE74. By contrast, ASD21 interacted predominantly through alkyl contacts involving LEU178 and ARG179, whereas ASD68 formed only hydrogen bonds, specifically with GLN175 and ARG179. LEU178 was also involved in hydrophobic interactions with ASD88. In addition, ASD179 established hydrogen bonds with IL6 at GLN175 and LEU178. Collectively, these residues have been reported as key binding amino acids of IL6 in the reference study.^29^

**Table 3:** Top five docking poses of ASD-derived compounds against IL6

Compound	Docking scores (kcal mol^−1^)	Interaction residue	
		Hydrogen bond	Other bonds
ASD38	−7.843	GLN75, SER76, SER176	LYS66, PHE74
ASD21	−7.682		LEU33, LEU178, ARG179
ASD179	−7.365	ASP34, GLN175, LEU178, ARG182	LEU33
ASD68	−7.000	GLU172, GLN175, SER176, ARG179	
ASD88	−7.055	ARG30	LEU178

**Fig. 7:**
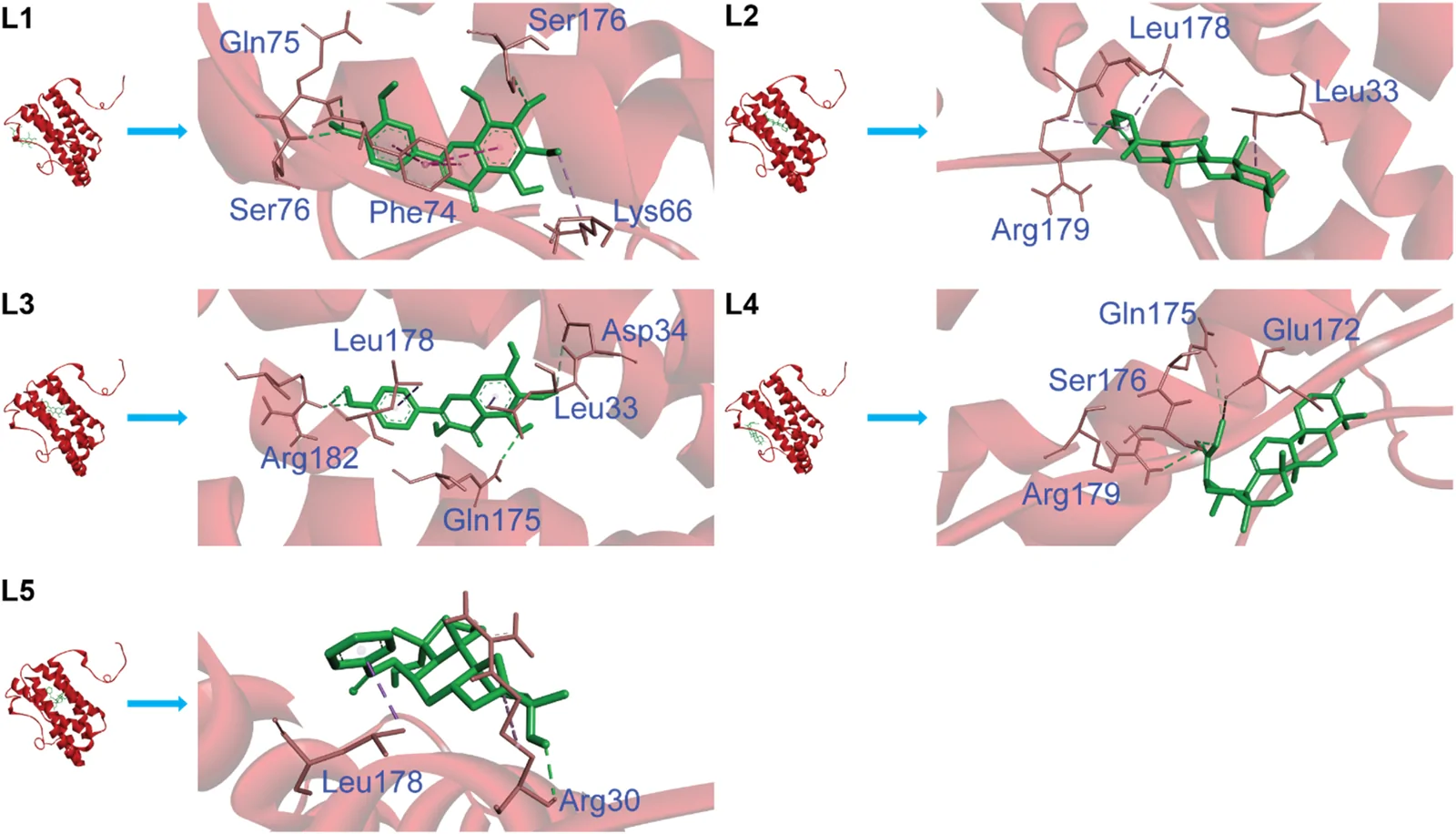
3D interaction between top 5 selected compounds and protein IL6. (L1) ASD38. (L2) ASD21. (L3) ASD179. (L4) ASD88. (L5) ASD68.

Specifically, ASD171 was identified as sitosterol, which has been reported to suppress the expression and secretion of pro-inflammatory cytokines, including TNF-α, IL6, and IL1β, in LPS-stimulated human umbilical vein endothelial cells (HUVECs).^30^ In addition, ASD38 (5-hydroxy-6,7,8,3′,4′-pentamethoxyflavone) has been shown to markedly ameliorate 12-*O*-tetradecanoylphorbol-13-acetate (TPA)-induced psoriasis-like skin lesions, accompanied by reductions in multiple inflammatory cytokines, including IL1β, IL17, IL4, IL6, TNF-α, and IFN-γ.^31^ Notably, ASD171 and ASD38 exhibited the most favorable binding affinities toward TNF-α and IL6, respectively, in agreement with their previously documented anti-inflammatory properties. Given this consistency, these two ligands were not prioritized for further molecular dynamics simulations. Instead, zeorinone (ASD21) was selected as the only ASD constituent ranking among the top-two binders of both TNF-α (−7.093 kcal mol^−1^) and IL6 (−7.682 kcal mol^−1^), highlighting its potential as a dual-target inhibitor of the two principal cytokine hubs identified by network pharmacology. ASD179 was further selected to investigate an alternative binding mode to IL6 that is characterised by an extensive hydrogen bonding network involving four key residues, including ASP34, GLN175, LEU178, and ARG182. This interaction pattern reflects the greatest density of polar contacts observed among the ASD-derived constituents and is consistent with the binding mode reported for the reference IL6 ligand.

### Molecular dynamics simulations

Two independent 300 ns MD simulations were performed for each of the three TNF-α–ligand complexes, as well as for the apoprotein and control-ligand complex, to assess the reproducibility of their conformational dynamics and binding stability. The RMSD profiles (Table 4 and Fig. 8) revealed distinct stability patterns among the simulated systems. In the first simulation run, the four systems exhibited average protein RMSD values predominantly below 3 Å, indicating generally stable conformations, with the T2 complex showing the lowest average RMSD of 2.24 Å. The apoprotein displayed two relatively stable intervals (30–110 ns and 130–170 ns), followed by a moderate increase from approximately 2.5 Å to 3.5 Å around 270 ns, after which the system remained stable until the end of the simulation. By comparison, the T2 complex reached equilibrium at an early stage and preserved a highly stable conformation throughout nearly the entire trajectory, except for a brief increase to approximately 3.2 Å around 120 ns. Similarly, the T3 complex remained stable at around 2.0 Å during 10–125 ns and again over the final 50 ns, while maintaining an intermediate stable phase near 2.5 Å during the middle stage of the simulation. In contrast, the T1 complex remained relatively stable until approximately 250 ns, after which the RMSD increased and reached a maximum of 4.1 Å at around 290 ns. In the second simulation run, the protein RMSD profiles of the four systems were generally comparable to those observed in the first run, with no substantial differences in overall stability, as shown in Fig. 8. Notably, the T3 complex exhibited reduced RMSD fluctuations during the second run, with an oscillation amplitude of less than 0.5 Å, indicating greater conformational stability than that observed in the first simulation. With respect to ligand RMSD, ASD21 and ASD68 exhibited the greatest stability across both independent simulations, with average RMSD values of 0.44 Å and 1.61 Å, respectively. In contrast, the control ligand showed substantially greater fluctuations, with RMSD values ranging approximately from 2 to 5 Å throughout most of both simulation runs. Therefore, the first simulation run was selected as the representative trajectory for subsequent analyses of the remaining structural and dynamic parameters.

**Table 4:** The average and standard deviation of five parameters of complexes between TNF-α, IL6 and ligands

Protein with ligand	RMSD of protein (Å)	RMSD of ligand (Å)	RMSF of protein (Å)	*R*_g_ (Å)	SASA (nm^2^)
**TNF-α with ligand**					
(T0) apoprotein	2.59 ± 0.36		2.03 ± 1.22	17.15 ± 0.19	93.24 ± 2.33
(T1) ASD21	2.38 ± 0.32	0.44 ± 0.07	2.39 ± 1.38	17.09 ± 0.16	92.37 ± 2.79
(T2) ASD68	2.24 ± 0.21	1.61 ± 0.08	1.94 ± 1.24	17.27 ± 0.20	91.04 ± 1.86
(T3) 307^a^	2.28 ± 0.35	3.99 ± 0.58	2.73 ± 1.50	17.05 ± 0.13	91.59 ± 2.29

**IL6 with ligand**					
(L0) apoprotein	5.09 ± 0.57		2.36 ± 1.66	17.66 ± 0.28	122.53 ± 3.10
(L1) ASD21	3.92 ± 0.42	0.85 ± 0.07	1.77 ± 1.04	17.44 ± 0.25	118.38 ± 3.48
(L2) ASD179	4.14 ± 0.34	1.00 ± 0.29	1.50 ± 0.93	17.35 ± 0.14	119.04 ± 3.60

a Control ligand.

**Fig. 8:**
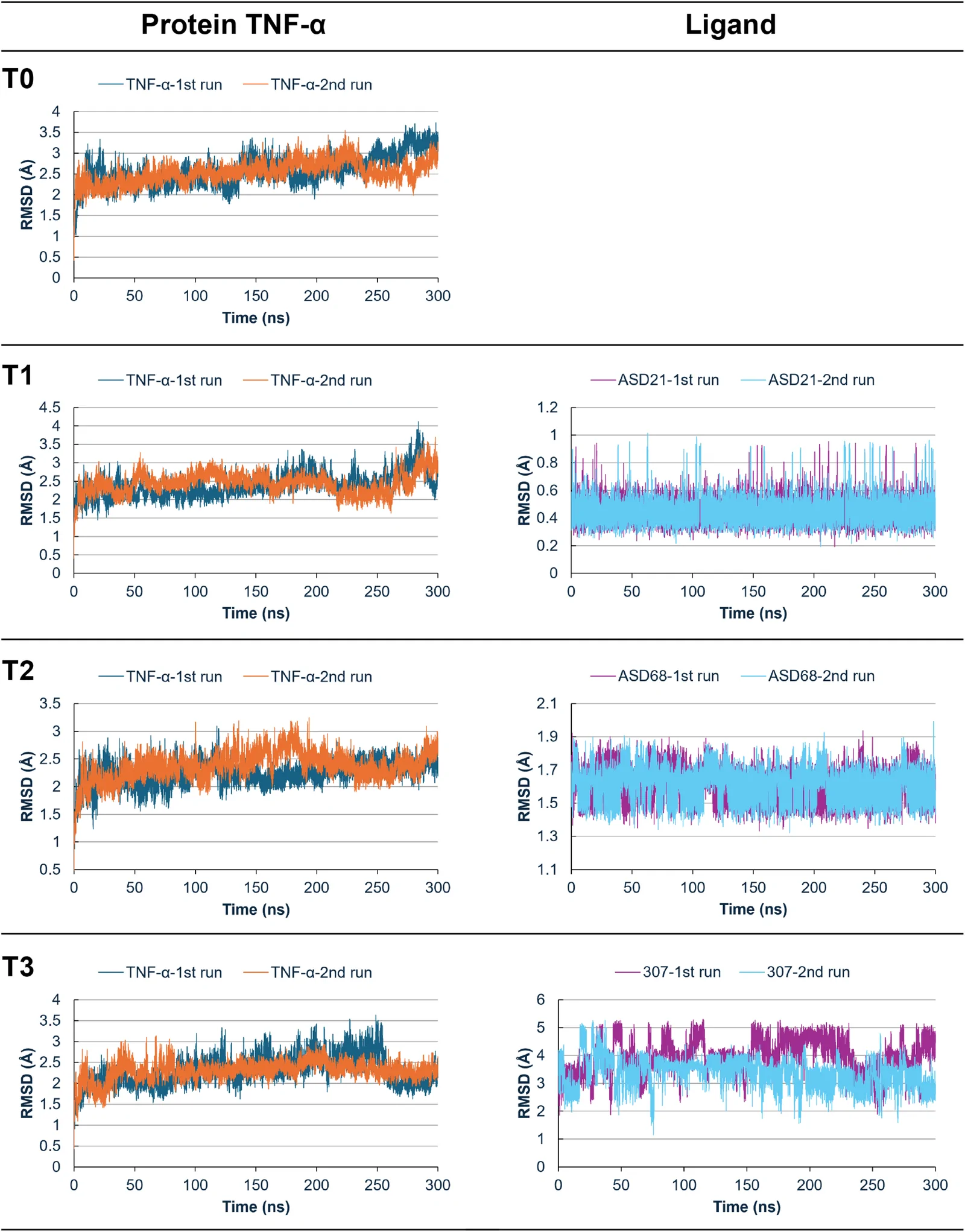
Backbone RMSD profiles of TNF-α in the apoprotein and ligand-bound states across two independent MD simulation runs. TNF-α was analyzed in the apoprotein form (T0) and in complex with ASD21 (T1), ASD68 (T2), and compound 307 (T3).

Residue-level flexibility was further evaluated using RMSF analysis of the TNF-α backbone and its ligand-bound complexes. As shown in Fig. 10A1, the overall fluctuation patterns were broadly similar among systems, and the key active-site residues, including TYR59, TYR119, GLY121, and TYR151, maintained low flexibility, with RMSF values consistently below 2 Å. In addition, the docked complexes and the apoprotein exhibited *R*_g_ values ranging from 16.52 to 17.79 Å with only minor variation (Fig. 10A2), indicating preserved structural compactness without substantial conformational rearrangement. The SASA profiles also remained relatively stable, fluctuating around an average of 92 nm^2^ and ranging from 84.20 to 104.74 nm^2^ (Fig. 10A3). These results suggest limited solvent exposure and effective burial of the ligands within the binding pocket, thereby supporting favorable binding interactions and enhanced complex stability.

A separate set of two independent 300 ns MD simulations was performed for each of the two IL6-ligand complexes, together with the IL6 apoprotein, to assess the reproducibility of their conformational dynamics and binding stability (Fig. 9). In the first simulation run, the RMSD profile of the L0 apoprotein exhibited two distinct stable phases separated at approximately 120 ns, with the first phase centered around 4 Å and the second around 5 Å. The L1 complex remained relatively stable at approximately 4 Å for most of the trajectory, although a pronounced decrease was observed between 130 and 200 ns, reaching a minimum RMSD of approximately 3 Å. In contrast, the L2 complex also remained near 4 Å for most of the simulation but displayed a marked increase between 170 and 200 ns, forming a plateau that approached 6 Å. In the second independent simulation, the overall protein RMSD profiles of the three systems were broadly consistent with those observed in the first run, with no substantial changes in their general stability patterns over the 300 ns trajectory. Notably, the transient increase in RMSD to approximately 6 Å observed for the L2 complex during the first simulation was not reproduced in the second independent run, suggesting variability in this localized conformational fluctuation between the two trajectories. In terms of ligand stability, ASD21 and ASD179 both exhibited low average ligand RMSD values (0.85 Å and 1.00 Å, respectively), suggesting stable retention within the binding site. However, ASD21 showed only a brief fluctuation with an amplitude of approximately 0.7 Å, whereas ASD179 exhibited comparatively larger fluctuations over the course of the simulation. Compared with the second simulation run, ASD21 exhibited ligand RMSD values that were broadly consistent with those observed in the first run, whereas ASD179 showed a noticeable decrease in RMSD between approximately 75 and 200 ns during the second simulation.

**Fig. 9:**
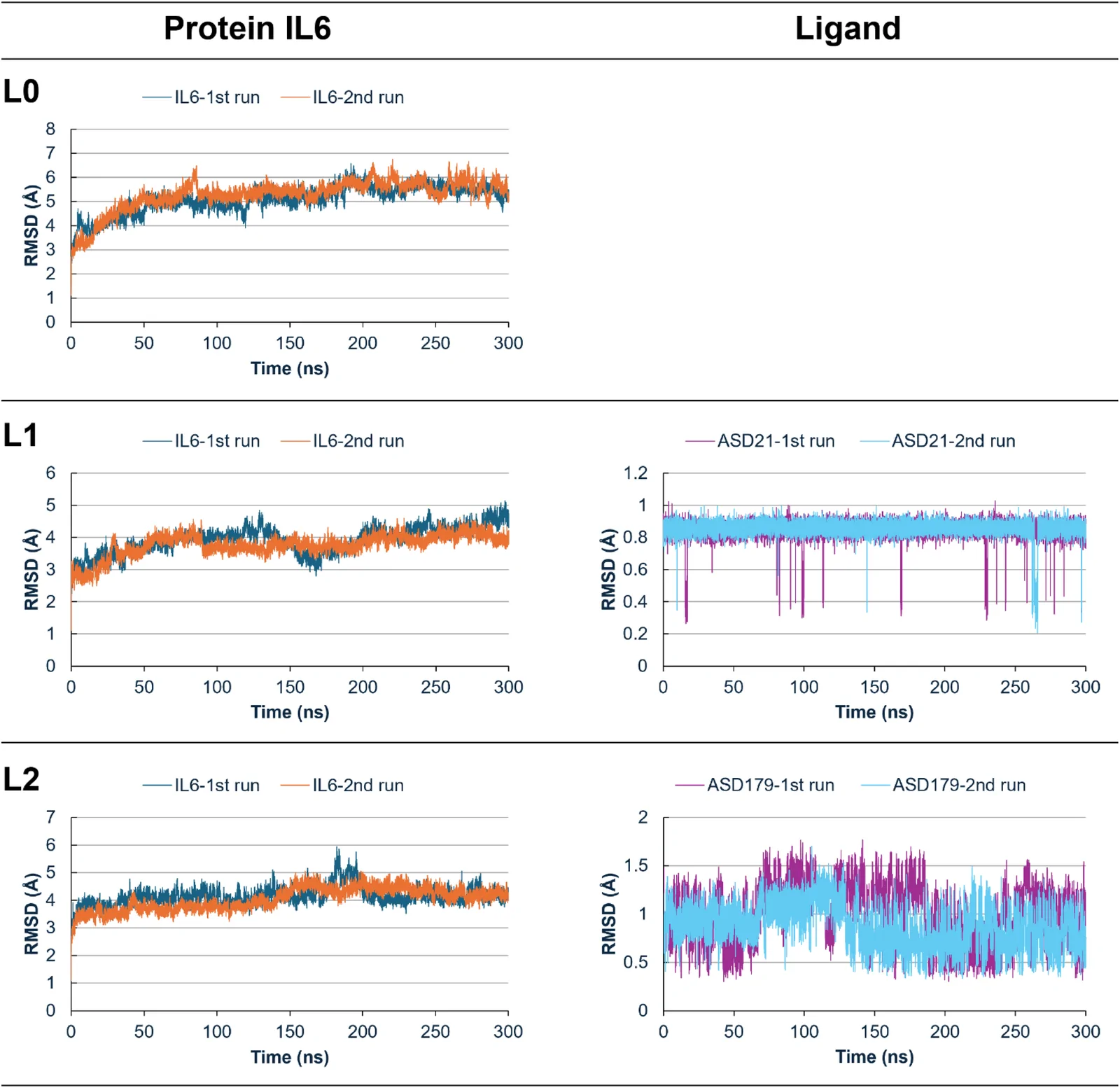
Backbone RMSD profiles of IL6 in the apoprotein and ligand-bound states across two independent MD simulation runs. IL6 was analyzed in the apoprotein form (L0) and in complex with ASD21 (L1) and ASD179 (L2).

RMSF analysis of the IL6 backbone and its ligand-bound complexes revealed generally similar fluctuation patterns across all systems. As illustrated in Fig. 10B1, the key binding-region residues PHE74, GLN75, GLN175, LEU178, and ARG179 remained relatively rigid, with RMSF values below 2 Å throughout the simulation. Furthermore, the ligand-bound complexes maintained *R*_g_ values ranging from 16.81 to 18.17 Å with only minor fluctuations (Fig. 10B2), indicating preserved structural compactness and the absence of major conformational rearrangements. The apoprotein showed a broadly similar *R*_g_ profile. However, a transient increase was observed between approximately 160 and 210 ns, during which the *R*_g_ value reached a maximum of 18.89 Å. The SASA profiles of both ligand-bound complexes remained relatively stable, with values ranging from 106.76 to 133.11 nm^2^ (Fig. 10B3), indicating limited solvent exposure and stable accommodation of the ligands within the binding region. The apoprotein exhibited a broadly comparable pattern, although a distinct transient increase was observed, reaching a maximum SASA value of 139.44 nm^2^. Collectively, these findings support stable ligand binding to IL6 and indicate favorable interactions that contribute to the overall conformational stability of the complexes.

**Fig. 10:**
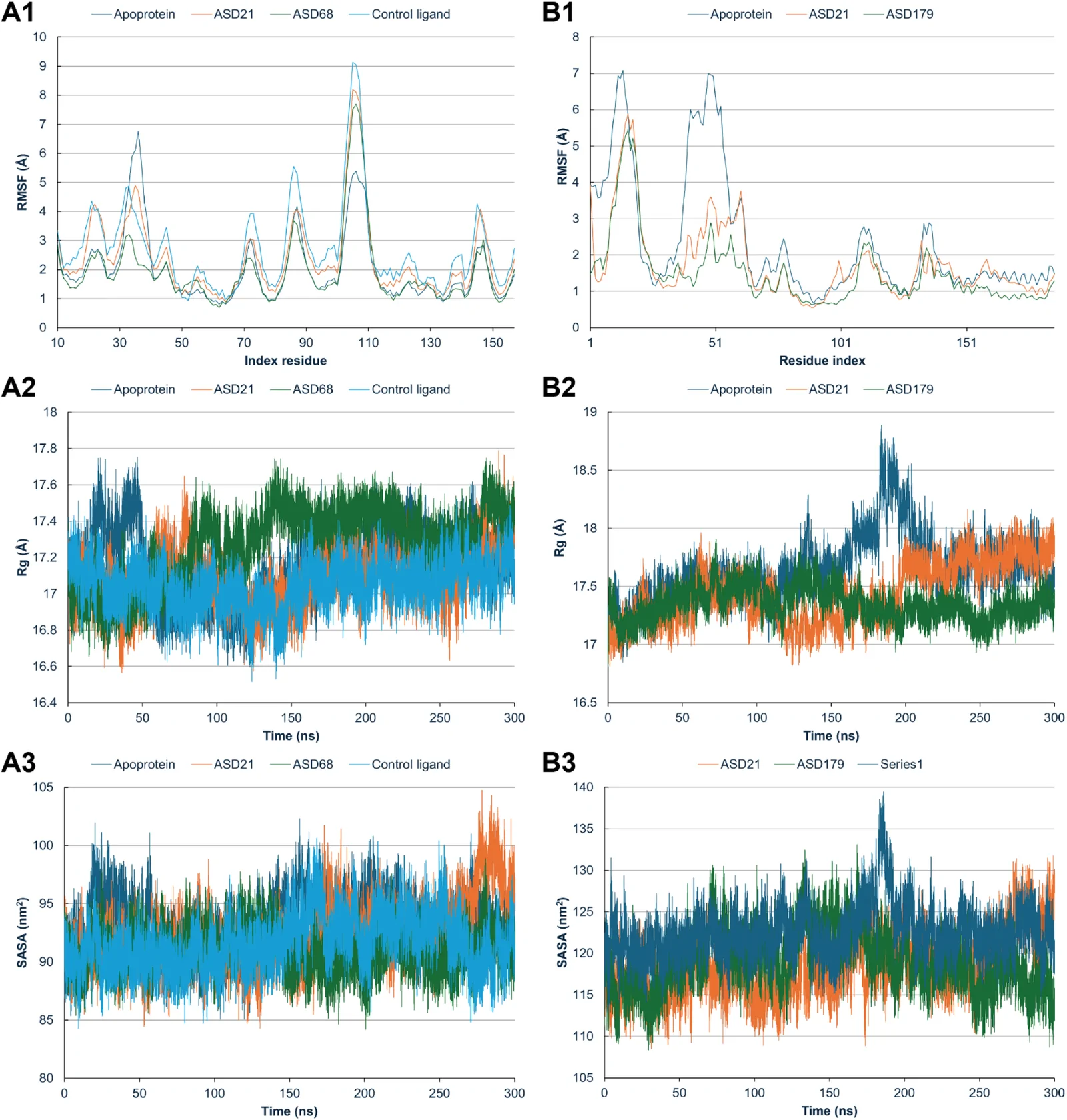
RMSF (A1 and B1), *R*_g_ (A2 and B2), and SASA (A3 and B3) profiles of ligand-bound TNF-α and IL6 complexes during MD simulations, where panels (A1–A3) correspond to TNF-α and panels (B1–B3) correspond to IL6.

Hydrogen-bond occupancy analysis, as summarized in Tables S4 and S5, demonstrated distinct ligand-specific interaction profiles with TNF-α. Two compounds showed particularly prominent binding behavior, with cumulative hydrogen-bond occupancies exceeding 70% at key residues. ASD21 exhibited a single dominant hydrogen-bond interaction with TYR59, reaching a total occupancy of 125.61%. By contrast, ASD68 formed two major hydrogen-bond interactions, involving TYR59 (86.77%) and TYR151 (78.09%). The control ligand displayed the most extensive interaction pattern overall, forming hydrogen bonds with TYR59 (207.08%), TYR119 (127.78%), and TYR151 (75.82%), with the TYR59 interaction showing the highest occupancy among all analyzed complexes. For IL6, only ASD21 exhibited a hydrogen-bond interaction, specifically with GLN175, with an occupancy of 83.84%, as presented in Table S6.

This study employed PCA, a widely used dimensionality reduction approach, to examine the conformational dynamics of TNF-α and IL6 in the presence of ligands, with particular emphasis on dominant structural motions and their association with binding stability. The PCA results revealed distinct distribution patterns of the first two principal components (PC1 and PC2) among the simulated systems. For TNF-α, the ligand-bound forms T1, T2, and T3 exhibited markedly tighter clustering in the PC1–PC2 conformational space than the apoprotein (T0), indicating reduced conformational dispersion and the presence of more stable dominant conformers, as shown in Fig. S3. In the IL6 systems, the PCA distributions of L0 and L1 were broadly comparable, whereas L2 showed a more diffuse and less densely clustered distribution, suggesting greater conformational variability relative to the other two systems.

Further conformational analysis was performed using the FEL derived from the first two principal components (PC1 and PC2). The resulting FEL plots, presented as three-dimensional surfaces in which color intensity reflects Gibbs free energy (dark blue-purple: 0–2 kJ mol^−1^; dark red: up to 21.40 kJ mol^−1^), provided an integrated view of the conformational freedom and stability of the simulated systems (Fig. 11). For TNF-α, the apoprotein (T0) exhibited two deep and broad energy minima, consistent with relatively restricted conformational flexibility and substantial structural stability during the simulation. In contrast, T1 displayed a single deep and extensive energy basin, suggesting the predominance of a more stable conformational state than that observed for the apoprotein. Meanwhile, T2 was characterized by a very small energy minimum and a high energy barrier, indicating the sampling of multiple conformational states and greater conformational heterogeneity. With respect to IL6, the L0 apoprotein showed two energy minima, one of which was elongated, implying the existence of more than one relatively stable conformational state. Compared with L0, L1 exhibited a small energy basin associated with a high energy barrier, together with two additional minima separated by relatively low energy barriers. By contrast, L2 appeared to sample the widest range of conformational states throughout the simulation, indicating greater conformational flexibility than the IL6 apoprotein.

**Fig. 11:**
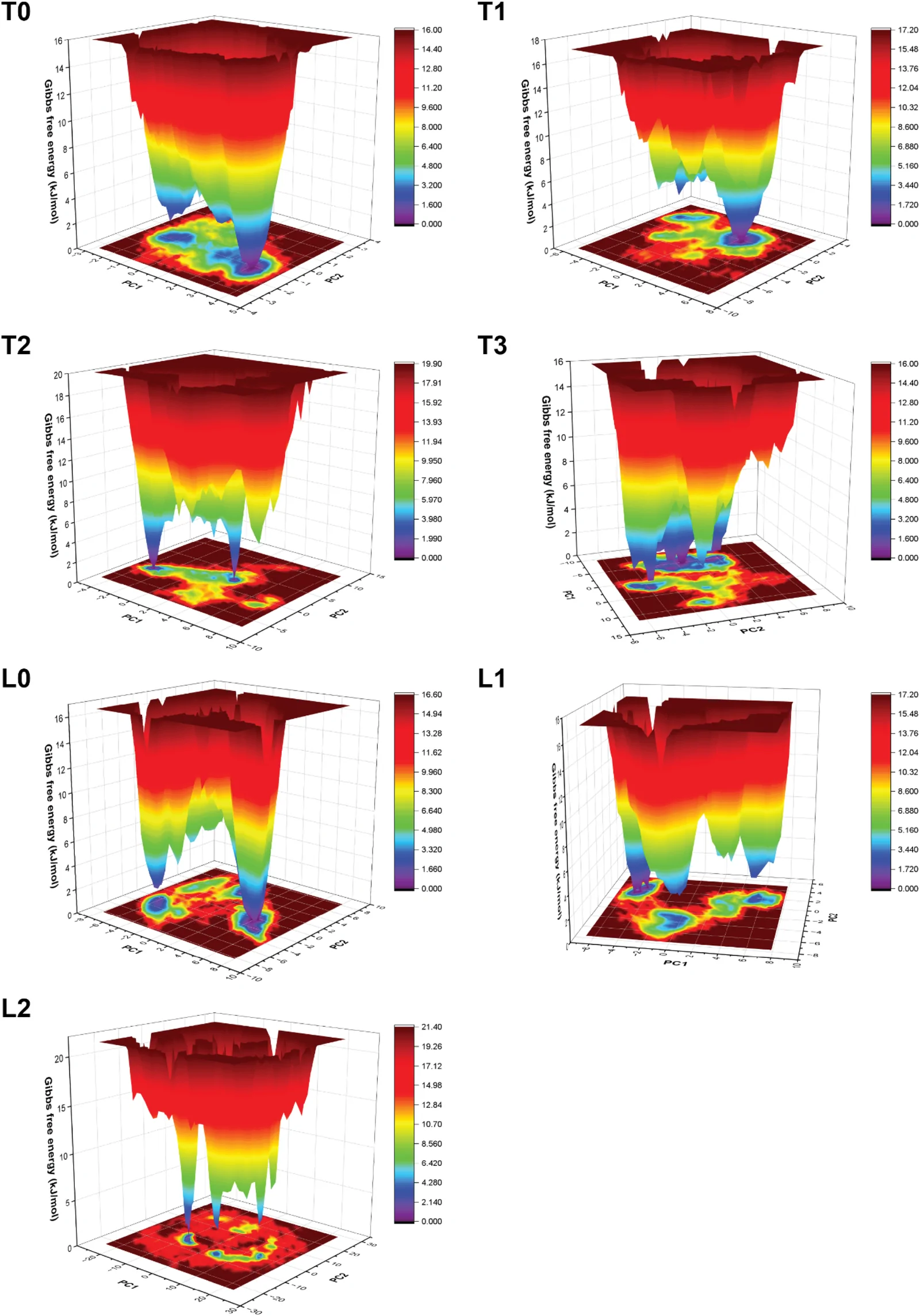
3D-FEL plots of TNF-α and IL6 in the apoprotein and ligand-bound states. TNF-α systems include the apoprotein (T0), ASD21-bound form (T1), ASD68-bound form (T2), and control ligand-bound form (T3), whereas IL6 systems comprise the apoprotein (L0), ASD21-bound form (L1), and ASD179-bound form (L2).

MM–PBSA binding free energy calculations were performed to quantitatively assess the interaction strengths of the selected ligands and control ligands with the target proteins TNF-α and IL6, as summarized in Table 5. These calculations were derived from the 300 ns molecular dynamics trajectories. Among the ligands evaluated against TNF-α, ASD21 exhibited the most favorable binding free energy (−21.52 kcal mol^−1^), followed by ASD68 (−19.23 kcal mol^−1^) and the control ligand (−17.73 kcal mol^−1^). Similarly, for IL6, ASD21 also showed the lowest binding free energy (−32.97 kcal mol^−1^), compared with ASD179 (−14.47 kcal mol^−1^). Taken together, the integrated analyses of complex stability, hydrogen-bond occupancy at key residues, PCA, FEL, and MM–PBSA binding free energy consistently indicate that ASD21 demonstrated favorable and stable binding to both targets among the MD-evaluated candidates. In particular, ASD21 should be evaluated *in vitro* to confirm its direct inhibitory effects on these target proteins, followed by additional studies to verify its broader anti-inflammatory activity and underlying mechanism of action.

**Table 5:** The calculation of binding free energy results of top selected ligands with TNF-α and IL6

Complexes	Δ*E*_vdW_ (kcal mol^−1^)	Δ*E*_elec_ (kcal mol^−1^)	Δ*G*_PB_ (kcal mol^−1^)	Δ*G*_SA_ (kcal mol^−1^)	Δ*G*_gas_ (kcal mol^−1^)	Δ*G*_sol_ (kcal mol^−1^)	Δ*G*_bind_ (kcal mol^−1^)
T1	−26.16 ± 3.07	−1.12 ± 1.59	8.32 ± 2.54	−2.57 ± 0.26	−27.27 ± 3.26	5.75 ± 2.40	−21.52 ± 2.30
T2	−24.76 ± 3.95	−1.07 ± 3.36	9.26 ± 3.18	−2.66 ± 0.35	−25.83 ± 4.85	6.60 ± 2.98	−19.23 ± 3.31
T3^a^	−26.00 ± 4.63	−3.66 ± 3.69	14.95 ± 4.44	−3.02 ± 0.45	−29.66 ± 6.46	11.94 ± 4.10	−17.73 ± 3.54
L1	−40.58 ± 7.06	−2.55 ± 5.65	14.22 ± 7.64	−4.06 ± 0.64	−43.13 ± 11.23	10.16 ± 7.16	−32.97 ± 5.60
L2	−19.28 ± 2.45	−7.69 ± 8.11	14.59 ± 6.41	−2.09 ± 0.22	−26.97 ± 8.54	12.50 ± 6.31	−14.47 ± 4.05

a Control ligand.

## Experimental

### Plant material and preparation of bioactive-enriched extract

The aerial parts of SD (ASD) were collected in Dong Thap Province, Vietnam, in September 2025, and taxonomically authenticated by Dr Huong-Giang Le. A voucher specimen (registry no. SD311291) was deposited at the College of Pharmacy, Taipei Medical University. The leaves were air-dried and pulverized into a fine powder, and 100 g of the material was extracted by ultrasonic-assisted extraction with 96% ethanol (3 × 300 mL) at room temperature, with each extraction cycle lasting 45 min. The combined extracts were subsequently concentrated under reduced pressure to yield the crude extract (CR, 17.8 g).

### Untargeted LC-HRMS/MS profiling of food bioactives

Fragment ion data were obtained using ultra-performance liquid chromatography coupled with tandem mass spectrometry (UPLC-MS/MS) on a Waters SYNAPT G2 LC/Q-TOF system (Waters Corporation, Milford, MA, USA). Chromatographic separation was carried out on a Waters Acquity UPLC BEH C18 column (2.1 mm × 100 mm, 1.7 µm particle size). The mobile phase consisted of acetonitrile (A) and water, both containing 0.1% formic acid. The gradient program was set as follows: 0.01–7 min, 5–10% A; 7–15 min, 10–50% A; 15–17.5 min, 50–60% A; 17.5–25 min, 60% A; 25–27.5 min, 60–70% A; 27.5–28.5 min, 70–100% A; and 28.5–30 min, 100% A. The chromatographic separation was carried out at a flow rate of 0.5 mL min^−1^, with the column oven maintained at 40 °C. For analysis, 4.0 mg of the sample was dissolved in 1 mL of methanol, passed through a 0.45 µm membrane filter, and introduced into the system *via* an autosampler, using an injection volume of 5 µL. Mass spectral data were recorded across an *m*/*z* range of 100–2000 in both MS1 and MS2 modes. Fragmentation spectra were obtained under data-dependent acquisition (DDA) conditions, with the collision energy progressively varied from 10 to 50 eV to fragment up to five precursor ions per acquisition cycle. The resulting data, including fragment ion patterns, were subsequently processed using the MassFragment module implemented in MassLynx 4.1 software (Waters, MA, USA).

Raw LC-MS/MS datasets acquired in both ionization modes were first converted into mzML files using MSConvert and subsequently analyzed in MZmine 4. The processing workflow included mass detection, chromatogram construction, peak deconvolution, isotope grouping, feature alignment, blank filtering, gap filling, and ion identity networking. Metabolite annotation was performed by combining experimental MS^2^ spectral matching with *in silico* approaches using GNPS resources, MassBank of North America, the MS-DIAL ESI(+)-MS/MS MSP database, and a customized SIRIUS-compatible library constructed from 385 natural products of the *Scoparia* spp retrieved from Reaxys. Spectral matching required at least two MS^2^ scans, four matched fragment ions, and a minimum weighted cosine similarity score of 0.7. For ion identity annotation, positive-mode adducts included [M + H]^+^, [M + Na]^+^, [M + K]^+^, [M + NH_4_]^+^, [M + 2H]^2+^, [M + H + Na]^2+^, [M + H + NH_4_]^2+^, and [M + H + K]^2+^, while negative-mode annotation considered [M − H]^−^, [M + Cl]^−^, [M + CH_3_COO]^−^, [M + HCOO]^−^, [M + Br]^−^, [M − H − H_2_O]^−^, and [M + CF_3_COO]^−^. The final aligned feature list was exported for downstream analyses in GNPS, MetaboAnalyst, SIRIUS, and R, and chemical class prediction was performed using CANOPUS.

### Feature-based molecular networking and chemical class annotation

Feature-based molecular networking was constructed from MGF files exported from MZmine 4 and analyzed through the GNPS platform. Both precursor and fragment mass tolerances were defined at 0.02 Da. Network generation required a minimum of six matched fragment ions and a cosine similarity score of 0.7, and these same criteria were also used for spectral library annotation. Separate GNPS jobs were generated for positive- and negative-ion datasets. The GNPS job results are available at the following links: negative ion mode (https://gnps.ucsd.edu/ProteoSAFe/status.jsp?task=b8ecda9ec29245efbad99bb3f185c613), positive ion mode (https://gnps.ucsd.edu/ProteoSAFe/status.jsp?task=fa9a4f01ce8b40df8744e86a61b73b96).

### *In silico* screening of absorption-related properties and putative protein targets of identified bioactives

Bioactive compounds from SD were identified based on LC-MS/MS analysis. Their drug-likeness, Caco-2 permeability (>−5.15 log cm s^−1^), and oral bioavailability (≥30%) were evaluated using ADMETlab 3.0,^16^ and these parameters were applied as screening criteria prior to subsequent analyses. Putative protein targets of the selected compounds were then predicted using the SwissTargetPrediction platform^17^ and further supplemented with additional targets obtained from a traditional Chinese medicine database. To identify inflammation-related genes, relevant entries were collected from the DisGeNET,^18^ OMIM,^32^ and GeneCards^19^ databases using the keyword “Inflammation” and restricting the search to *Homo sapiens*. GeneCards results were further filtered using a relevance score threshold of ≥5.0, and duplicate entries across the databases were removed to generate a unified set of inflammation-associated genes.

### Network analysis linking food bioactives to inflammation-related targets

By employing a Venn diagram approach, common targets were identified through the intersection of candidate targets with disease-associated genes. To identify the major ASD constituents potentially involved in inflammation, a compound-target-disease network was established by integrating the selected compounds, overlapping targets, and inflammation-associated genes, and the resulting network was examined using Cytoscape v3.8.2.^33^ The overlapping targets were then submitted to the STRING database (version 12.0) to construct a protein–protein interaction (PPI) network, with the analysis limited to *Homo sapiens*. Network generation was performed using a high-confidence interaction score of 0.700 and a false discovery rate (FDR) threshold of 5%, and the complete STRING network was retained for subsequent evaluation. The resulting PPI output was downloaded in TSV format and further processed in Cytoscape for downstream topological analysis. The resulting PPI data were exported in TSV format and imported into Cytoscape for further analysis. Evaluation of network topological parameters, including node degree, betweenness centrality (BC), and closeness centrality (CC), was performed using the CytoNCA plugin. Nodes exhibiting high BC values were considered critical for network communication, whereas those with elevated CC values were indicative of central positioning within the network. Key targets were identified by selecting nodes with values exceeding the median thresholds for BC and CC, ultimately yielding a set of 10 core targets.^34,35^

### GO enrichment and KEGG pathway analysis

To elucidate the functional characteristics and biological relevance of the top ASD-associated targets in inflammation, Gene Ontology (GO) and Kyoto Encyclopedia of Genes and Genomes (KEGG) enrichment analyses were performed. The analyses were performed using ShinyGO v0.82 (https://bioinformatics.sdstate.edu/go/), encompassing GO categories including biological process, molecular function, and cellular component, as well as KEGG pathways. The top 20 enriched GO terms and KEGG pathways, ranked according to −log_10_(FDR), were visualized using bubble and bar plots.^36,37^

### Protein and ligand preparation

The three-dimensional crystal structures of the key target proteins, including tumor necrosis factor alpha (TNF-α) and interleukin-6 (IL6), were obtained from the Protein Data Bank (PDB) under the accession codes 2AZ5 and 1ALU, respectively. SPD304 (PDB ligand ID: 307), the co-crystallized small-molecule inhibitor of TNF-α in the 2AZ5 structure, was used as the reference ligand. SPD304 has been experimentally demonstrated to bind directly to TNF-α and promote dissociation of the biologically active TNF-α trimer.^28^ Therefore, it was selected as an experimentally validated reference for docking protocol validation and comparison with the ASD-derived compounds. The protein structures were prepared using AutoDockTools 1.5.6 by removing crystallographic water molecules and non-essential heteroatoms, adding polar hydrogen atoms, assigning Kollman charges, and converting the structures into PDBQT format for subsequent docking analysis. The docking search space was defined by specifying the center coordinates and dimensions of the grid box for each target protein. For TNF-α, the grid was centered at *x* = −19.410, *y* = 74.651, and *z* = 33.850 Å based on the position of the co-crystallized ligand (307). For IL6, the grid was centered at *x* = −13.019, *y* = −13.542, and *z* = 3.410 Å to encompass the previously reported key binding residues.^29,38^ For both targets, the dimensions of the docking search space were set to 35 × 35 × 35 Å to ensure adequate coverage of the corresponding binding regions. In parallel, the chemical structures of the selected compounds were energy-minimized using Open Babel,^39^ and subsequently converted into the appropriate PDBQT format for molecular docking.

### Molecular docking

Molecular docking was employed to evaluate the interactions between the selected compounds and their respective target proteins based on structural complementarity and predicted binding affinity. Docking simulations were performed using AutoDock Vina.^40^ An exhaustiveness value of 8 was applied, and up to 10 binding poses were generated for each ligand. The pose with the most favorable predicted binding affinity was selected for subsequent interaction analysis. The resulting protein–ligand complexes were further examined and visualized using Discovery Studio 2021 to characterize intermolecular interactions and identify the amino acid residues involved in ligand binding.

### Molecular dynamics simulations

Based on docking-derived initial conformations, protein–ligand complexes were subjected to molecular dynamics simulations using GROMACS 2024.1 (ref. 41 and 42) to examine their stability and binding behavior.^43^ Protein structures were prepared using the CHARMM-GUI platform,^44,45^ while ligand structures were protonated in Discovery Studio Visualizer and subsequently parameterized through the SwissParam server.^46^ Protein topologies were generated in GROMACS with the pdb2gmx module using the CHARMM36 force field, which was applied consistently throughout the simulations.^47^ The apoproteins and protein–ligand systems were placed in cubic solvent boxes containing TIP3P water molecules,^48,49^ with a minimum solute-to-box-edge distance of 10 Å. To neutralize the systems, appropriate counterions were added,^50^ and 0.15 M NaCl was introduced to mimic physiological ionic strength.^51,52^ Prior to equilibration, each system underwent energy minimization using the steepest descent method for 50 000 steps to relieve unfavorable contacts and improve structural stability.^53^ Equilibration was then performed in two successive stages, consisting of an *NVT* ensemble at 300 K followed by an *NPT* ensemble at 1 atm, with each stage run for 100 ps.^36,54^ Production simulations were then performed for 300 ns, with trajectory data recorded at 0.01 ns intervals. Following the completion of the simulations, structural and dynamic properties were comprehensively characterized through the calculation of root mean square deviation (RMSD), root mean square fluctuation (RMSF), radius of gyration (*R*_g_), and solvent-accessible surface area (SASA), while ligand–residue interactions were further examined by analyzing hydrogen bond formation and visualized using VMD software.^55^ Based on a donor–hydrogen–acceptor (D–H⋯A) angle exceeding 120° and a donor–acceptor distance within the defined cutoff, hydrogen bonds were identified.^56^ In this study, the cumulative hydrogen-bond occupancy between a ligand and a given amino acid residue was calculated using the following equation:^50^
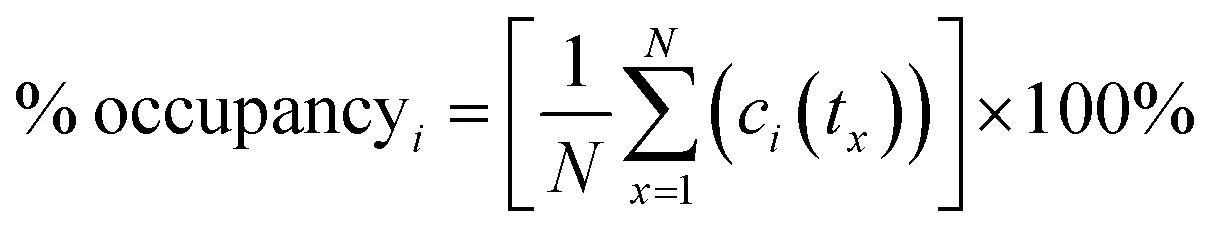
where *N* represents the total number of analyzed trajectory frames, and *c*_*i*_(*t*_*x*_) denotes the number of hydrogen-bond contacts formed between the ligand and residue *i* at frame *t*_*x*_. The frequency values of residues can reach over 100% as they have formed multiple contacts with the ligand.

### Principal component and free energy landscape analysis

Principal component analysis (PCA) was employed to capture the dominant collective motions of the protein in response to ligand binding by converting correlated atomic movements into a set of orthogonal principal components (PCs). Examination of the primary components (PC1 and PC2), represented through a three-dimensional free energy landscape (FEL),^57^ enabled evaluation of the interplay between conformational dynamics, energetic profiles, and structural stability. This approach facilitated the identification of energetically favorable conformational states and provided insight into the potential binding efficiency of the ligands.

### MM–PBSA binding energy

Binding free energies were determined *via* the gmx_MMPBSA approach, employing a single trajectory derived from GROMACS simulations performed with the CHARMM36 force field.^58^ The MM–PBSA method was applied to frames extracted from the final 100 ns of the simulation, with one frame sampled per nanosecond. The binding free energy (Δ*G*_bind_) was computed according to the following relationship:^59^

1 Δ*G*_bind_ = Δ*G*_complex_ − (Δ*G*_protein_ + Δ*G*_ligand_)

Although the MM–GBSA approach is computationally less demanding, the MM–PBSA method was selected due to its more rigorous theoretical basis for estimating binding affinities. Accordingly, binding free energies were estimated for each protein–ligand complex using the corresponding MD trajectories *via* the MM–PBSA approach.^60^

### Anti-inflammatory activity in LPS-stimulated RAW 264.7 macrophages

#### Cell viability assay

The cytotoxic effects of silibinin and thymol in RAW264.7 cells (ATCC, Manassas, VA, USA) were assessed using the MTT assay.^61^ RAW264.7 cells were seeded into 96-well plates at a density of 5 × 10^3^ cells per well and allowed to adhere for 24 h. The cells were then treated with the test samples at concentrations of 100, 50, and 10 µg mL^−1^ for 2 h, followed by incubation for 24 h in the absence or presence of lipopolysaccharide (LPS, 1 µg mL^−1^). Subsequently, 100 µL of MTT solution (0.5 mg mL^−1^) was added to each well, and the plates were incubated for an additional 3 h. After removal of the supernatant, the formazan crystals generated by MTT reduction were dissolved in 100 µL of DMSO. Absorbance was measured at 570 nm using a microplate reader. Each treatment condition was evaluated in triplicate.

#### ELISA assay

RAW264.7 cells were pretreated with the ethanol extract for 2 h, followed by stimulation with LPS (1 µg mL^−1^) for 24 h. Dexamethasone served as the positive control. After incubation, the culture supernatants were collected and clarified by centrifugation. The levels of TNF-α and IL6 released into the culture medium were then quantified using commercially available mouse ELISA kits, following the manufacturer's instructions.

#### Statistical analysis

All statistical analyses for the *in vitro* assays were performed with GraphPad Prism (version 8.0.2). The results are presented as mean ± standard deviation (SD). Group differences were assessed by one-way analysis of variance (ANOVA), with multiple comparisons subsequently examined using Tukey's post hoc test. Statistical significance was defined as *p* < 0.05.

## Conclusions

This study provides an integrated chemical, experimental, and computational characterization of the aerial parts of *Scoparia dulcis* as a potential source of dietary anti-inflammatory bioactives. LC-HRMS/MS coupled with GNPS feature-based molecular networking enabled the annotation of 184 metabolites in the ASD extract, predominantly shikimates and phenylpropanoids (44.0%) and terpenoids (39.7%), including well-documented food bioactives such as β-sitosterol, polymethoxyflavones, catechin, and naringin. Consistent with this chemically diverse profile, the ASD extract significantly attenuated LPS-induced TNF-α and IL6 secretion in RAW 264.7 macrophages at 50–100 µg mL^−1^ without apparent cytotoxicity, while IL6 inhibition at 100 µg mL^−1^ exceeded that achieved by the steroidal reference dexamethasone. To further investigate the molecular basis underlying these effects, network pharmacology and KEGG enrichment analyses linked ASD constituents to inflammation-related pathways of nutritional relevance, including the TNF, JAK–STAT, PI3K–AKT, and AGE–RAGE signaling pathways. Molecular docking and molecular dynamics simulations further identified zeorinone (ASD21) as a stable dual binder of TNF-α and IL6. Collectively, these findings suggest that SD may represent a promising candidate for the development of functional foods, herbal beverages, or dietary supplements targeting chronic inflammation, potentially through the additive or synergistic effects of structurally diverse bioactives rather than a single dominant compound. Future studies should compare ethanolic extracts with traditional aqueous preparations, evaluate total phenolic and flavonoid contents together with antioxidant capacity, assess gastrointestinal bioaccessibility, isolate and validate the activity of zeorinone and other lead compounds, and ultimately confirm efficacy through controlled human intervention studies.

## Author contributions

Ngoc-Thac Pham: writing – review & editing, writing – original draft, investigation, formal analysis, data curation, conceptualization. Thanh-Sang Nguyen: writing – original draft, investigation, formal analysis, data curation, conceptualization. Ngo Thi My Duyen: visualization, validation, investigation. Huong-Giang Le: visualization, validation, investigation. Thuy-Tien Thi Phan: visualization, validation, investigation. Quoc-Dung Tran Huynh: visualization, validation, investigation. Phuong Vu Luu: visualization, validation. Quang-Hien Kha: visualization, validation. Vu Duc Loi: visualization, validation. Duc-Toan Nguyen: visualization, validation. Chung-Kuang Lu: visualization, validation. Kuei-Hung Lai: writing – review & editing, visualization, validation, supervision, methodology, investigation, data curation, conceptualization.

## Conflicts of interest

There are no conflicts to declare.

## Supplementary Material

RA-OLF-D6RA06433G-s001

## Data Availability

The data supporting this article have been included as part of the supplementary information (SI). Supplementary information is available. See DOI: https://doi.org/10.1039/d6ra06433g.
