# Supplementary material for: GNPS-assisted identification of bioactive constituents from Scoparia dulcis and their anti-inflammatory mechanisms via network pharmacology and experimental validation
Source: RSC Adv. 2026 Oct 7;16(46):60650–70. doi: 10.1039/d6ra06433g (PMC13643700; doi:10.1039/d6ra06433g)
Supplement: Supplementary file 1 [file RA-016-D6RA06433G-s001.pdf]

Supplementary Information (SI) for RSC Advances.
This journal is © The Royal Society of Chemistry

SUPPLEMENTARY MATERIAL

GNPS-Assisted Identification of Bioactive Constituents from *Scoparia dulcis* and Their Anti-Inflammatory Mechanisms via Network Pharmacology and Experimental Validation

Ngoc-Thac Pham [a,b], Thanh-Sang Nguyen [b,c], Ngo Thi My Duyen [d], Huong-Giang Le [a], Thuy-Tien Thi Phan [e], Quoc Dung Huynh Tran [e], Phuong Vu Luu [f], Quang-Hien Kha [g], Vu Duc Loi [h], Duc-Toan Nguyen [i], Chung-Kuang Lu [b,j,k,*], Kuei-Hung Lai [b,f,l,m,**]

[a] *Department of Pharmacognosy and Traditional Pharmacy, School of Pharmacy, University of Medicine and Pharmacy at Ho Chi Minh City, Ho Chi Minh City 700000, Vietnam.*

[b] *PhD Program in Clinical Drug Development of Herbal Medicine, College of Pharmacy, Taipei Medical University, Taipei 110301, Taiwan.*

[c] *Food Technology Faculty, Saigon Technology University, Ho Chi Minh City 700000, Vietnam.*

[d] *Faculty of Pharmacy, Lac Hong University, 10 Huynh Van Nghe street, Tran Bien ward, Dong Nai city, Viet Nam.*

[e] *Institute of Pharmaceutical Education and Research, Binh Duong University, Ho Chi Minh city, Vietnam.*

[f] *Faculty of Pharmacy, Ton Duc Thang University, Ho Chi Minh City, Vietnam.*

[g] *International Ph.D. Program in Medicine, College of Medicine, Taipei Medical University, Taipei 110301, Taiwan.*

[h] *Vietnam University of Traditional Medicine, 2 Tran Phu, Dai Mo, Hanoi, Vietnam.*

[i] *University of Information Technology, Computer science, Ho Chi Minh city, Vietnam*

[j] *National Research Institute of Chinese Medicine, Ministry of Health and Welfare, Taipei 112026, Taiwan.*

[k] *Department of Life Sciences and Institute of Genome Sciences, National Yang Ming Chiao Tung University, No. 155, Sec. 2, Li-Nong Street, Beitou, Taipei, 112304, Taiwan.*

[l] *Traditional Herbal Medicine Research Center, Taipei Medical University Hospital, Taipei 110301, Taiwan.*

[m] *National Museum of Marine Biology and Aquarium, Pingtung 944401, Taiwan.*

* Corresponding author. Chung-Kuang Lu. Email: cklu@nricm.edu.tw

** Corresponding author. Kuei-Hung Lai. Email: kueihunglai@tmu.edu.tw

Table legend

Table S1. Annotation summary of metabolites identified in ASD extract, including retention time, mass accuracy, adduct type, and chemical classification

Table S2. Compounds identified from SD that satisfied the criteria for Lipinski's rule, Caco–2 permeability, and oral bioavailability

Table S3. Molecular docking results of ASD-derived compounds against TNF-α and IL-6

Table S4. Hydrogen bond occupancy between top ligands and main amino acids of TNF-α.

Table S5. Total hydrogen bond occupancy (over 70 %) between top ligands and main amino acids of TNF-α.

Table S6. Hydrogen bond occupancy between top ligands and main amino acids of IL6.

Table S7. Total hydrogen bond occupancy (over 70 %) between top ligands and main amino acids of IL6.

Table S1. Annotation summary of metabolites identified in ASD extract, including retention time, mass accuracy, adduct type, and chemical classification

No	Condition	Smiles	Compound_Name	Adduct	tR	Observed	Theoretica	△mmu	Error	Classifier_Pathway	Classifier_Superclass	Classifier_Class
1	Pos+Neg	C1=CC(=C(C=C1C2=CC(=O)C3=C(C=C(C=C3O2)O)O)O)O	Luteolin	[M+H]+	5.29	287.0538	287.0550	1.21	4.23	Shikimates and Phenylpropanoids	Flavonoids	Flavones
2	Pos+Neg	COC1=CC=C(C=C1)C2=CC(=O)C3=C(C=C(C=C3O2)O)O	acacetin	[M+H]+	5.94	285.0746	285.0757	1.15	4.03	Shikimates and Phenylpropanoids	Flavonoids	Flavones
3	Pos+Neg	COC1=C(C=C(C=C1)C2=CC(=O)C3=C(C(=C(C(=C3O2)OC)O)OC)O)O	acerosin	[M+H]+	7.40	361.0904	361.0918	1.39	3.86	Shikimates and Phenylpropanoids	Flavonoids	Flavones
4	Pos+Neg	COC1=C(C2=C(C=C1O)OC(=CC2=O)C3=CC=C(C=C3)O)O	Hispidulin	[M+H]+	7.26	301.0699	301.0707	0.76	2.54	Shikimates and Phenylpropanoids	Flavonoids	Flavones
5	Pos+Neg	C1=CC(=CC=C1C2=C(C(=O)C3=C(C=C(C=C3O2)O)O)O)O	kaempferol	[M+H]+	4.16	287.0537	287.0550	1.31	4.58	Shikimates and Phenylpropanoids	Flavonoids	Flavonols
6	Pos+Neg	C1=CC(=CC=C1C2=CC(=O)C3=C(O2)C=C(C(=C3O)O)O)O	scutellarein	[M+H]+	4.41	287.0537	287.0550	1.31	4.58	Shikimates and Phenylpropanoids	Flavonoids	Flavones
7	Neg	COC1=C(C(=C2C(=C1)OC(=CC2=O)C3=CC=C(C=C3)O)O)OC	Scrophulein	[M-H]-	5.99	313.0736	313.0718	1.84	5.87	Shikimates and Phenylpropanoids	Flavonoids	Flavones
8	Neg	C1=CC(=CC=C1C2=CC(=O)C3=C(C=C(C=C3O2)O)O)O	apigenine	[M-H]-	5.09	269.0470	269.0455	1.45	5.40	Shikimates and Phenylpropanoids	Flavonoids	Flavones
9	Pos	C[C@@H]1CC[C@@H]2[C@@H]1[C@H]3[C@H](C3(C)C)CCC2=C	aromandendrene	[M+Na]+	11.19	227.1782	227.1770	1.18	5.20	Terpenoids	Sesquiterpenoids	Aromadendrane sesquiterpenoids
10	Pos	CC(C1=CC(=C(C(=C1)CC=C(C)CO)O)CC=C(C)CO)OC	(±)-scopacoumaricin B	[M+NH4]+	6.38	338.2313	338.2326	1.28	3.79	Shikimates and Phenylpropanoids	Unknown	Unknown
11	Pos	CC(=CCC1=CC(=CC(=C1O)CC=C(C)CO)C(CO)O)CO	(±)-scopacoumaricin E	[M+Na]+	8.05	345.1677	345.1672	0.46	1.33	Alkaloids	Unknown	Unknown
12	Pos	C1=CC(=C(C=C1O)O)CC#N	(2,4-dihydroxyphenyl)acetonitrile	[M+H]+	3.26	150.0541	150.0550	0.85	5.69	Shikimates and Phenylpropanoids	Unknown	Unknown
13	Pos	C/C=C(\C)/C(=O)O[C@H]1C[C@@H]2[C@H](C1=C)[C@@H]3[C@@H]([C@H](CC2=C)O)C(=C)C(=O)O3	(E)-2-Methyl-but-2-enoic acid (3aR,4S,6aR,8S,9aR,9bR)-4-hydroxy-3,6,9-trimethylene-2-oxo-dodecahydro-azuleno[4,5-b]furan-8-yl ester	[M+H]+	10.77	345.1685	345.1696	1.15	3.33	Terpenoids	Sesquiterpenoids	Guaiane sesquiterpenoids
14	Pos	COC1=C(C=CC(=C1)/C=C/C(=O)O)O	Ferulic acid	[M+H]+	5.27	195.0642	195.0652	0.98	5.05	Shikimates and Phenylpropanoids	Phenylpropanoids (C6-C3)	Cinnamic acids and derivatives
15	Pos	C/C(=C\CO)/C(=O)O[C@H]1C[C@@H]2[C@H](C1=C)[C@@H]3[C@@H]([C@H](CC2=C)OC(=O)/C(=C/CO)/C)C(=C)C(=O)O3	(E)-4-Hydroxy-2-methyl-but-2-enoic acid (3aR,4S,6aR,8S,9aR,9bR)-4-((E)-4-hydroxy-2-methyl-but-2-enoyloxy)-3,6,9-trimethylene-2-oxo-dodecahydro-azuleno[4,5-b]furan-8-yl ester	[M+NH4]+	10.58	476.2293	476.2279	1.41	2.96	Terpenoids	Sesquiterpenoids	Guaiane sesquiterpenoids

16	Pos	C/C(=C\CO)/C(=O)O[C@H]1C[C@@H]2[C@H](C1=C)[C@@H]3[C@@H]([C@H](CC2=C)O)C(=C)C(=O)O3	(E)-4-Hydroxy-2-methyl-but-2-enoic acid (3aR,4S,6aR,8S,9aR,9bR)-4-hydroxy-3,6,9-trimethylene-2-oxo-dodecahydro-azuleno[4,5-b]furan-8-yl ester	[M+H-H2O]+	10.38	343.1528	343.1540	1.20	3.50	Terpenoids	Sesquiterpenoids	Guaiane sesquiterpenoids
17	Pos	COC1=C2C(=CC3=C1OCO3)C[C@H](OC2=O)C4=CC=CC=C4	(S)-4-methoxy-7-phenyl-7,8-dihydro[1,3]dioxolo[4,5-g]isochromen-5-one	[M+H]+	11.09	299.0900	299.0914	1.40	4.68	Shikimates and Phenylpropanoids	Coumarins	Isocoumarins
18	Pos	C[C@H]1C/C=C/[C@H]2[C@H]3[C@](O3)([C@H]([C@@H]4[C@]2(C(=O)N[C@H]4CC5=CC=C(C=C5)O)OC(=O)C/C=C(\C1=O)/C)C)C	[12]-cytochalasin	[M+H]+	4.27	480.2421	480.2381	4.04	8.40	Alkaloids	Pseudoalkaloids	Cytochalasan alkaloids
19	Pos	CC(C)(C)OC(=O)N1CCCC(C1)C(=O)O	1-(tert-butoxycarbonyl)piperdine-3-carboxylic-acid	[M+NH4]+	21.98	247.1656	247.1652	0.37	1.49	Alkaloids	Unknown	Unknown
20	Pos	CC[C@@H](C#CC#CC=CC(CCCCCC=C)O)O	1,2-dihydrodendroarboreol B	[M+H-H2O]+	10.83	243.1732	243.1743	1.14	4.69	Fatty acids	Fatty acyls	Fatty alcohols
21	Pos	CC(C)C1=C2CC[C@@]3([C@@H]([C@]2(CC1)C)CC[C@H]4[C@]3(CC(=O)C5[C@@]4(CCCC5(C)C)C)C)C	17(21)-Hopen-6-one	[M+H]+	14.79	425.3762	425.3778	1.59	3.74	Terpenoids	Triterpenoids	Hopane and Moretane triterpenoids
22	Pos	C=CCCCCC/C=C\CC#CC#CC(=O)CCO	1-hydroxy-heptadeca-9c,16-diene-4,6-diyn-3-one	[M+H]+	13.95	259.1682	259.1693	1.06	4.07	Fatty acids	Fatty acyls	Fatty alcohols
23	Pos	CCCCCCCCCCCCCCCCCC(=O)OC[C@H](COP(=O)([O-])OCC[N+](C)(C)C)O	1-Stearoyl-sn-glycero-3-phosphocholine	[M+H]+	12.30	524.3688	524.3711	2.27	4.32	Fatty acids	Glycerophospholipids	Glycerophosphocholines
24	Pos	CC1(CC2C3=CCC4C5(CCC(C(C5CCC4(C3(CCC2(C(C1)O)C(=O)O)C)C)(C)C)O)C)C	22-hydroxyoleanolic acid	[M+H-H2O]+	10.88	455.3504	455.3520	1.57	3.45	Terpenoids	Triterpenoids	Oleanane triterpenoids
25	Pos	C[C@@]12CCC3[C@@](C1CC=C4[C@]2(CC[C@@]5(C4CC(CC5)(C)C)C(=O)O[C@H]6[C@@H]([C@H]([C@@H]([C@H](O6)CO)O)O)O)C)(C[C@@H]([C@@H](C3(C)C)O)O)C	2β,3β-dihydroxyolean-12-en-28-oic acid 28-O-β-D-glucopyranoside	[M+H-H2O]+	14.22	617.4000	617.4048	4.79	7.77	Terpenoids	Triterpenoids	Oleanane triterpenoids
26	Pos	CC1C(C(C(C(O1)OCC2C(C(C(C(O2)OC3=CC(=C4C(=C3)OC(=CC4=O)C5=CC=C(C=C5)O)O)O)O)O)OC(=O)C)OC(=O)C)O	3-(acetyloxy)-5-hydroxy-6-methyl-2-[(3,4,5-trihydroxy-6-{[5-hydroxy-2-(4-hydroxyphenyl)-4-oxo-4H-chromen-7-yl]oxy}oxan-2-yl)methoxy]oxan-4-yl acetate	[M+Na]+	4.26	685.1737	685.1739	0.20	0.30	Shikimates and Phenylpropanoids	Flavonoids	Flavones

27	Pos	COC1=CC2=C(C=C1)N(C(=O)O2)OC	3,6-dimethoxy-benzoxazolin-2(3H)-one	[M+H]+	3.24	196.0593	196.0604	1.13	5.78	Alkaloids	Unknown	Unknown
28	Pos	CC(=CCCC(C)(C=C)O)C	Linalool	[M+Na]+	14.22	177.1265	177.1250	1.52	8.56	Terpenoids	Monoterpenoids	Acyclic monoterpenoids
29	Pos	C[C@H](CCC(=O)OC1=CC2=C(C=C1)[C@H]3CC[C@]4([C@H]([C@@H]3CC2)CCC4=O)C)[C@H]5CC[C@@H]6[C@@]5(CC[C@H]7[C@H]6[C@@H]([C@@H]([C@H]8[C@@]7(CC[C@H](C8)O)C)O)O)C	3a, 6a, 7a-hayochlic-acid_estrone	[M+NH4]+	12.40	678.4767	678.4728	3.89	5.73	Terpenoids	Steroids	Cholane steroids
30	Pos	C[C@H](/C=C/C(=O)NCCCCCN)[C@H]1CC[C@@H]2[C@@]1([C@H](C[C@H]3[C@H]2[C@@H](C[C@H]4[C@@]3(CC[C@H](C4)O)C)OC(=O)CC(C)O)OC(=O)CC(C)O)C	3a,7a,12a-delta-22-cholic-acid_3-hydroxybutanoic-acid_3-hydroxybutanoic-acid_pentane-1,5-diamine	[M+NH4]+	19.12	680.4776	680.4844	6.84	10.05	Terpenoids	Steroids	Unknown
31	Pos	CC1=NCCC(N1)C(=O)O[C@@H]2CC[C@]3([C@@H](C2)C[C@H]([C@@H]4[C@@H]3C[C@@H]([C@]5([C@H]4CC[C@@H]5[C@H](C)/C=C/C(=O)NCCCNCCCCN)C)OC(=O)C(=C)C)O)C	3a,7a,12a-delta-22-cholic-acid_methacrylic-acid_2-methyl-1,4,5,6-tetrahydropyrimidine-4-carboxylic-acid_N1-(3-aminopropyl)butane-1,4-diamine	[M+H-H2O]+	19.13	708.5088	708.5058	2.95	4.17	Alkaloids	Ornithine alkaloids	Polyamines
32	Pos	COC1=C(C=CC(=C1)C(=O)CCO)O	β-hydroxypropiovanillone	[M+H]+	3.28	197.0801	197.0808	0.73	3.73	Shikimates and Phenylpropanoids	Unknown	Unknown
33	Pos	C[C@H]1[C@@H]([C@H]([C@H]([C@H](O1)O[C@H]2[C@H](CO[C@H]([C@@H]2O)O[C@H]3CC[C@@]4([C@H]5CC[C@@H]6[C@@H]7CC(CC[C@@]7(CC[C@]6([C@@]5(CC[C@H]4C3(C)C)C)C)C(=O)O)(C)C)C)O)O)O[C@H]8[C@@H]([C@H]([C@@H](CO8)O)O)O)O	scoposide E	[M+NH4]+	21.26	886.5555	886.5522	3.26	3.67	Terpenoids	Triterpenoids	Oleanane triterpenoids
34	Pos	C[C@H]1[C@@H]2[C@H](CC(=C)[C@@H]3C[C@@H](C(=C)[C@@H]3[C@H]2OC1=O)O)OC(=O)C4=CC(=C(C=C4)OC)OC	3β-hydroxy-8α-(3,4-dimethoxybenzoyloxy)-11β,13-dihydro-1αH,5αH,6βH,7αH-guai-4(15),10(14)-dien-6,12-olide	[M+K]+	4.49	467.1506	467.1467	3.94	8.43	Terpenoids	Sesquiterpenoids	Guaiane sesquiterpenoids
35	Pos	C/C(=C\CO)/CC[C@@H]1C(=C)[C@@H]([C@H]([C@@H]2[C@@]1(CCC[C@]2(C)C(=O)O)C)OC(=O)C3=CC=CC=C3)OC(=O)C	4-epi-7α-acetoxyscoparic acid A	[M+NH4]+	11.03	516.2940	516.2956	1.58	3.05	Terpenoids	Diterpenoids	Labdane diterpenoids
36	Pos	C[C@H]1[C@@H]([C@H]([C@H]([C@@H](O1)C2=C3C(=C(C(=C2O)[C@H]4[C@@H]([C@H]([C@@H]([C@H](O4)CO)O)O)O)O)C(=O)C=C(O3)C5=CC=C(C=C5)O)O)O)O	5,7-dihydroxy-2-(4-hydroxyphenyl)-6-[(2S,3R,4R,5S,6R)-3,4,5-trihydroxy-6-	[M+H]+	4.42	579.1686	579.1708	2.23	3.85	Shikimates and Phenylpropanoids	Flavonoids	Flavones

			(hydroxymethyl)oxan-2-yl]-8-[(2S,3R,4R,5R,6S)-3,4,5-trihydroxy-6-methyloxan-2-yl]chromen-4-one								
37	Pos	C1C(C(C(C(O1)C2=C3C(=C(C(=C2O)C4C(C(C(C(O4)CO)O)O)O)O)C(=O)C=C(O3)C5=CC=C(C=C5)O)O)O)O	Vicenin III	[M+H]+	4.11	565.1530	565.1552	2.18	3.86	Shikimates and Phenylpropanoids	Flavonoids	Flavones
38	Pos	COC1=C(C=C(C=C1)C2=CC(=O)C3=C(C(=C(C(=C3O2)OC)OC)OC)O)OC	5-hydroxy-6,7,8,3',4'-pentamethoxyflavone	[M+H]+	10.15	389.1221	389.1231	0.99	2.55	Shikimates and Phenylpropanoids	Flavonoids	Flavones
39	Pos	CCC/C=C/C#CCCCO[C@H]1[C@@H]([C@H]([C@@H]([C@H](O1)CO)O)O)O	6E-decen-4-ynol 1-O-β-D-glucopyranoside	[M+Na]+	0.87	337.1592	337.1622	2.96	8.77	Fatty acids	Unknown	Unknown
40	Pos	CC1=CCC(C2(C1C3C(CC2)C(=C)C(=O)O3)C)O	6-hydroxy-5a,9-dimethyl-3-methylene-3a,4,5,5a,6,7,9a,9b-octahydronaphtho[1,2-b]furan-2(3H)-one	[M+H-H2O]+	10.24	231.1368	231.1380	1.16	5.00	Terpenoids	Sesquiterpenoids	Eudesmane sesquiterpenoids
41	Pos	C/C(=C\CO)/CC[C@H]1[C@]2(CCC[C@@]([C@@H]2[C@@H]([C@H]([C@]13CO3)OC(=O)C)OC(=O)C4=CC=CC=C4)(C)C(=O)O)C	7α-acetoxy-8,17β-epoxyscoparic acid A	[M+H-H2O]+	9.55	497.2489	497.2534	4.48	9.01	Terpenoids	Diterpenoids	Labdane diterpenoids
42	Pos	C=C1C[C@@H]([C@@H]2[C@@H]([C@@H]3[C@H]1C[C@@H]([C@]34CO4)O)OC(=O)C2=C)O	8-desacylrepin	[M+H]+	6.41	279.1215	279.1227	1.20	4.30	Terpenoids	Sesquiterpenoids	Guaiane sesquiterpenoids
43	Pos	CC1=C[C@@H]2[C@@H](CC=C([C@@]2(CC1)O)C)C(C)(C)O	8-ene-pubinernoid C	[M+H-H2O]+	5.61	219.1732	219.1743	1.14	5.21	Terpenoids	Sesquiterpenoids	Cadinane sesquiterpenoids
44	Pos	C1C(C(C(C(O1)C2=C(C(=C3C(=C2O)C(=O)C=C(O3)C4=CC=C(C=C4)O)C5C(C(C(CO5)O)O)O)O)O)O)O	apigenin 6,8-di-C-α-L-arabinopyranoside	[M+H]+	4.48	535.1429	535.1446	1.72	3.21	Shikimates and Phenylpropanoids	Flavonoids	Flavones
45	Pos	C[C@H]1[C@@H]([C@H]([C@H]([C@@H](O1)OC[C@@H]2[C@H]([C@@H]([C@H]([C@@H](O2)OC3=CC(=C4C(=C3)OC(=CC4=O)C5=CC=C(C=C5)O)O)O)O)O)OC(=O)C)OC(=O)C)O	apigenin 7-O-α-L-2,3-di-O-acetylrhamnopyranosyl-(1->6)-β-D-glucopyranoside	[M+H]+	4.61	663.1873	663.1920	4.66	7.03	Shikimates and Phenylpropanoids	Flavonoids	Flavones
46	Pos	COC1=CC(=C2C(=C1)OC(=C(C2=O)OC)C3=CC(=C(C=C3)O)OC)O	ayanin	[M+H]+	8.34	345.0956	345.0969	1.28	3.71	Shikimates and Phenylpropanoids	Flavonoids	Flavonols
47	Pos	CC/C=C/C(=O)O[C@H]1C[C@H]2[C@H]([C@H]3[C@]1([C@H](CC3)[C@H](C)CC(=O)NCCCCN=C(N)N)C)C(=O)C[C@H]4[C@@]2(CC[C@H](C4)OC(=O)CCCC#C)C	C23-nor-3a,7k,12a-cholic-acid_hex-5-ynoic-acid_(E)-pent-2-enoic-acid_1-(4-aminobutyl)guanidine	[M+H-H2O]+	19.12	663.4516	663.4480	3.60	5.43	Terpenoids	Steroids	Cholestane steroids
48	Pos	C1=CC(=C(C=C1CCOC2C(C(C(C(O2)CO)OC(=O)C=CC3=CC(=C(C=C3)O)O)O)O)O)O	calceolarioside A	[M+H]+	4.29	479.1526	479.1548	2.19	4.57	Shikimates and Phenylpropanoids	Phenylethanoids (C6-C2), Phenylpropanoids (C6-C3)	Cinnamic acids and derivatives, Phenylethanoids

49	Pos	C[C@]12CC[C@@H](C([C@@H]1CC[C@@]3([C@@H]2CC=C4[C@]3(CC[C@@]5([C@H]4CC(CC5)(C)C)C(=O)O)C)C)(C)C)O[C@H]6[C@@H]([C@H]([C@@H]([C@H](O6)C(=O)O)O)O)O	calenduloside E	[M+Na]+	12.43	655.3794	655.3817	2.25	3.44	Terpenoids	Triterpenoids	Oleanane triterpenoids
50	Pos	CC(C)CCOC(=O)CCCCCCCCCCCC(=O)OC(CC(=O)O)C[N+](C)(C)C	Carnitine_Isoamyl alcohol_mesaconic acid	[M]+	12.57	458.3462	458.3476	1.41	3.09	Fatty acids	Fatty esters	Fatty acyl carnitines
51	Pos	CC1(C[C@H]2[C@H]1CCC3(C(O3)CCC2=C)C)C	Caryophyllene oxide	[M+H-H2O]+	11.17	203.1784	203.1794	1.03	5.05	Terpenoids	Sesquiterpenoids	Caryophyllane sesquiterpenoids
52	Pos	CC1(CCC2(CCC3(C(=CCC4C3(CCC5C4(CCC(C5(C)C)O)C)C)C2C1)C)C(=O)O)C	Caryophyllin	[M+H-H2O]+	14.26	439.3553	439.3571	1.76	4.00	Terpenoids	Triterpenoids	Oleanane triterpenoids
53	Pos	C=C1C[C@@H]([C@@H]2[C@@H]([C@@H]3[C@H]1C[C@@H](C3=C)O)OC(=O)C2=C)O	deacylcynaropicrin	[M+Na]+	3.75	285.1108	285.1097	1.07	3.76	Terpenoids	Sesquiterpenoids	Guaiane sesquiterpenoids
54	Pos	CCCCCCCCCC1=CC=C(C=C1)OCCOCCOCCOCCOCCOCCOCCOCCOCCO	Nonoxynol-9	[M+H]+	12.43	617.4237	617.4259	2.22	3.60	Fatty acids	Glycerophospholipids	Unknown
55	Pos	C[C@@]1(C[C@H]([C@]2(C(=C1)[C@@H]([C@]3([C@@]4([C@@]2(CCCC4(C)C)CO3)O)O)O)O)O)C=C	Diaporthein A	[M+H]+	9.27	367.2102	367.2115	1.31	3.58	Terpenoids	Diterpenoids	Pimarane and Isopimarane diterpenoids
56	Pos	C[C@@]1(C[C@H]([C@]2(C(=C1)C(=O)[C@]3([C@@]4([C@@]2(CCCC4(C)C)CO3)O)O)O)O)C=C	Diaporthein B	[M+H-H2O]+	10.64	347.1840	347.1853	1.30	3.74	Terpenoids	Diterpenoids	Pimarane and Isopimarane diterpenoids
57	Pos	C[C@H](/C=C/[C@H](C)C(C)C)[C@H]1CCC2=C3C=CC4=CC(=O)CC[C@@]4([C@H]3CC[C@]12C)C	ergosta-4,6,8(14),22-tetraen-3-one; (22E,24R)-ergosta-4,6,8(14),22-tetraen-3-one	[M+H]+	17.62	393.3136	393.3152	1.59	4.05	Terpenoids	Steroids	Ergostane steroids
58	Pos	C[C@@H](CCC(=C)C(C)C)[C@@H]1CC[C@]2([C@]1(CCC3=C2CCC4[C@@]3(CC[C@@H](C4(C)C)O)C)C)C	euphorbol	[M+H-H2O]+	15.86	423.4026	423.3985	4.07	9.62	Terpenoids	Triterpenoids	Lanostane, Tirucallane and Euphane triterpenoids
59	Pos	CC1OC2=C(C=C(C(=C2OC)C)C)C(=O)O1	eutyscoparol F	[M+H]+	8.95	223.0956	223.0965	0.88	3.97	Polyketides	Aromatic polyketides	Unknown
60	Pos	CCCCCCCC1=C(C=C(C(=C1C=O)O)CC=C(C)C)O	flavoglaucin	[M+H-H2O]+	9.94	287.1992	287.2006	1.36	4.72	Polyketides	Aromatic polyketides	Unknown
61	Pos	C1=CC(=C(C=C1C2=CC(=O)C3=C(O2)C=C(C(=C3O)C4C(C(C(C(O4)CO)O)O)O)O)O)O	Homoorientin	[M+H]+	3.92	449.1060	449.1078	1.84	4.09	Shikimates and Phenylpropanoids	Flavonoids	Flavones
62	Pos	C1=CC(=C(C=C1C2=C(C(=O)C3=C(C=C(C=C3O2)O)O)O[C@H]4[C@@H]([C@H]([C@H]([C@H](O4)CO)O)O)O)O)O	Hyperoside; quercetin-3-O-galactoside	[M+NH4]+	2.79	482.1252	482.1293	4.10	8.50	Shikimates and Phenylpropanoids	Flavonoids	Flavonols

63	Pos	C=C1C[C@@H]([C@@H]2[C@@H]([C@@H]3[C@H]1C[C@@H]([C@]34CO4)O)OC(=O)C2=C)OC(=O)C(=C)CO	janerin	[M+H-H2O]+	9.07	345.1356	345.1333	2.34	6.77	Terpenoids	Sesquiterpenoids	Guaiane sesquiterpenoids
64	Pos	CNC[C@@H](C1=CC(=C(C=C1)O)O)O	L-epinephrine	[M+H-H2O]+	1.19	166.0853	166.0863	0.95	5.75	Alkaloids	Tyrosine alkaloids	Phenylethylamines
65	Pos	CC(=CCC1=C(C2=C(C=C1O)OC(=C(C2=O)O)C3=CC=C(C=C3)O)O)C	Licoflavonol	[M+H-H2O]+	10.12	337.1057	337.1070	1.35	4.00	Shikimates and Phenylpropanoids	Flavonoids	Flavonols
66	Pos	C1=CC(=C(C=C1C2=CC(=O)C3=C(C(=C(C=C3O2)OC4C(C(C(C(O4)CO)O)O)O)C5C(C(C(C(O5)CO)O)O)O)O)O)O	Lutonarin	[M+H-H2O]+	5.17	593.1484	593.1501	1.70	2.86	Shikimates and Phenylpropanoids	Flavonoids	Flavones
67	Pos	CCCCCCCCCCCCCCCC(=O)OCC(COP(=O)([O-])OCC[N+](C)(C)C)O	LysoPhosphatidylcholine_16_0	[M+H]+	11.00	496.3379	496.3398	1.87	3.76	Fatty acids	Glycerophospholipids	Glycerophosphocholines
68	Pos	C[C@H]1[C@@H](CC[C@]2([C@@H]1C3=CCC4[C@]5(CCC(=O)C(C5CC[C@]4([C@@]3(CC2)C)C)(C)C)C)C)C(=O)OC	methyl ifflaionate	[M+H]+	19.28	469.3657	469.3676	1.92	4.09	Terpenoids	Triterpenoids	Lupane triterpenoids, Ursane and Taraxastane triterpenoids
69	Pos	COC1=CC=C(C=C1)C2=CC(=O)C3=C(C(=C(C(=C3O2)OC)O)OC)O	nevadensin	[M+H]+	9.07	345.0958	345.0969	1.08	3.13	Shikimates and Phenylpropanoids	Flavonoids	Flavones
70	Pos	COC[C@@H](CC1=CC(=C(C=C1)OC)OC)[C@H](CC2=CC3=C(C(=C2)OC)OCO3)COC	niranthin	[M+Na]+	5.61	455.2023	455.2040	1.72	3.78	Shikimates and Phenylpropanoids	Lignans	Dibenzylbutane lignans
71	Pos	C[C@H]1CC[C@H]([C@@]2(C1=C[C@H](CC2)C(C)(C)O)C)O	nitidumine D	[M+Na]+	10.25	261.1837	261.1825	1.20	4.60	Terpenoids	Sesquiterpenoids	Eudesmane sesquiterpenoids
72	Pos	C/C/1=C\CCC(=C[C@@H]2[C@@H]([C@H](C1)OC(=O)C(=C)CO)C(=C)C(=O)O2)CO	onopordopicrin	[M+Na]+	4.72	371.1475	371.1465	0.99	2.68	Terpenoids	Sesquiterpenoids	Germacrane sesquiterpenoids
73	Pos	CCC1=C(C2=NC1=CC3=C(C4=C(CC(=C5[C@H]([C@@H](C(=CC6=NC(=C2)C(=C6C)C=C)N5)C)CCC(=O)O)C4=N3)O)C)C	Pyropheophorbide a	[M+H]+	15.51	535.2684	535.2704	1.97	3.67	Alkaloids	Unknown	Unknown
74	Pos	C[C@@]1(CC[C@@H](O1)C(C)(C)O)[C@H](CC[C@@](C)(C=C)O)O	rel-(3S,6S,7R,10R)-7,10-epoxy-3,7,11-trimethyldodec-1-ene-3,6,11-triol	[M+Na]+	12.14	295.1891	295.1880	1.12	3.80	Terpenoids	Monoterpenoids	Acyclic monoterpenoids
75	Pos	C[C@@]1(CC[C@]2(C(=C1)C(=O)[C@]3([C@@]4([C@@]2(CCCC4(C)C)CO3)O)O)O)C=C	scopararane A	[M+H-H2O]+	9.27	331.1889	331.1904	1.48	4.48	Terpenoids	Diterpenoids	Pimarane and Isopimarane diterpenoids
76	Pos	C[C@@]1(CC[C@]2(C(=C1)C(=O)C(=C3[C@@]2(CCCC3(C)C)C)O)O)C=C	scopararane C	[M+H-H2O]+	15.88	299.1994	299.2006	1.16	3.86	Terpenoids	Diterpenoids	Pimarane and Isopimarane diterpenoids

77	Pos	C[C@@]1(CCC2=C([C@H]1O)[C@@H]([C@]3([C@@]4([C@@]2(CCCC4(C)C)CO3)O)O)O)C=C	scopararane F	[M+H-H2O]+	9.51	333.2039	333.2060	2.14	6.41	Terpenoids	Diterpenoids	Pimarane and Isopimarane diterpenoids
78	Pos	C[C@@]1(CCC2=C([C@H]1O)[C@@H](C(=O)[C@@]3([C@@]2(CCCC3(C)C)C)O)O)C=C	scopararane G	[M+H]+	9.92	335.2196	335.2217	2.09	6.22	Terpenoids	Diterpenoids	Pimarane and Isopimarane diterpenoids
79	Pos+Neg	C/C(=C/[C@H]([C@H](C(C)(C)O)O)O)/C=C	(+)-(3R,4R,5Z)-2,6-trimethyl-5,7-octadiene-2,3,4-triol	[M+Na]+	6.49	209.1164	209.1148	1.59	7.59	Terpenoids	Monoterpenoids	Acyclic monoterpenoids
80	Pos+Neg	COC[C@@H]1CC2=CC3=C(C(=C2[C@@H]([C@H]1COC)C4=CC(=C(C=C4)OC)OC)OC)OCO3	(+/-)-nirtetralin	[M+NH4]+	9.20	448.2316	448.2330	1.38	3.07	Shikimates and Phenylpropanoids	Lignans	Arylnaphthalene and aryltetralin lignans
81	Pos+Neg	CC(=CCC1=CC(=CC(=C1O)CC=C(C)CO)C(COC)O)CO	(±)-scopacoumaricin A	[M+H]+	10.02	337.1995	337.2009	1.45	4.30	Unknown	Unknown	Unknown
82	Pos+Neg	C/C(=C\C=O)/C(=O)O[C@H]1CC(=C)[C@@H]2C[C@@H]([C@@]([C@@H]2[C@@H]3[C@@H]1C(=C)C(=O)O3)(CCl)O)O	(1R,3S,4S,5S,6S,7R,8S)-4β-(chloromethyl)-3β,4α-dihydroxy-8α-(3-formyl-2-methyl-propenoyloxy)-1αH,5αH,6βH,7αH-guai-10(14),11(13)-dien-6,12-olide	[M+H]+	2.98	411.1244	411.1205	3.89	9.47	Terpenoids	Sesquiterpenoids	Guaiane sesquiterpenoids
83	Pos+Neg	C/C(=C\CO)/CCC1C(=C)[C@@H]([C@H]([C@@H]2[C@@]1(CCC[C@@]2(C)CO)C)OC(=O)C3=CC=CC=C3)OC(=O)C	(1S,2S,4aR,8R,8aR)-2-acetoxy-4-((E)-5-hydroxy-3-methylpent-3-en-1-yl)-8-(hydroxymethyl)-4a,8-dimethyl-3-methylenedecahydronaphthalen-1-yl benzoate	[M+H]+	12.43	485.2856	485.2898	4.17	8.58	Terpenoids	Diterpenoids	Labdane diterpenoids
84	Pos+Neg	COC1=CC2=C(C=C1)NC(=O)[C@@H](O2)O[C@H]3[C@@H]([C@H]([C@@H]([C@H](O3)CO)O)O)O	(2R)-7-methoxy-2H-1,4-benzoxazin-3(4H)-one 2-O-β-galactopyranoside	[M+H]+	3.25	358.1119	358.1133	1.36	3.79	Alkaloids	Unknown	Unknown
85	Pos+Neg	CC1=CC(=O)C[C@]2([C@H]1C[C@@H]([C@H](C2)OC(=O)C)C(C)(C)O)C	(2S,3S,4aR,8aS)-3-(1-hydroxy-1-methylethyl)-5,8a-dimethyl-7-oxo-1,2,3,4,4a,7,8,8a-octahydronaphthalen-2-yl acetate	[M+H]+	11.00	295.1892	295.1904	1.19	4.02	Terpenoids	Sesquiterpenoids	Eudesmane sesquiterpenoids

86	Pos+Neg	C[C@]1(CC=C[C@](O1)(C)CC[C@@H](C(C)(C)O)O)C=C	(3S,7S,10S)-3,7,11-trimethyl-3,7-epoxy-1,5-dodecadien-10,11-diol	[M+NH4]+	7.05	272.2209	272.2220	1.12	4.11	Terpenoids	Sesquiterpenoids	Farnesane sesquiterpenoids
87	Pos+Neg	C[C@]12CCCC([C@@H]1C(=O)OC(=O)C3=CC(CC[C@]23O)(C)C=C)(C)C	(4aS,11aS,11bS)-11a-hydroxy-4,4,9,11b-tetramethyl-9-vinyl-1,2,3,4,4a,9,10,11,11a,11b-decahydrodibenzo[c,e]oxepine-5,7-dione	[M+H-H2O]+	9.30	315.1943	315.1955	1.17	3.71	Terpenoids	Diterpenoids	Pimarane and Isopimarane diterpenoids
88	Pos+Neg	C[C@]1(CCC[C@]2([C@H]1[C@@H](C[C@@H]3[C@@]24CC[C@]([C@H](C3)C4)(C)O)OC(=O)C5=CC=CC=C5)C)CO	(4R,4aR,5R,6aR,8R,9S,11aS,11bS)-9-hydroxy-4-(hydroxymethyl)-4,9,11b-trimethyltetradecahydro-8,11a-methanocyclohepta[a]naphthalen-5-yl benzoate	[M+H-H2O]+	14.56	409.2716	409.2737	2.12	5.18	Terpenoids	Diterpenoids	Podocarpane diterpenoids
89	Pos+Neg	C[C@@]12CC[C@]3(C1)[C@@H](C[C@H](C4[C@@]3(CCC[C@@]4(C)C(=O)O)C)OC(=O)C5=CC=CC=C5)CC2=O	(4R,5R,6aS,9R,11aS,11bS)-5-(benzoyloxy)-4,9,11b-trimethyl-8-oxotetradecahydro-9,11a-methanocyclohepta[a]naphthalene-4-carboxylic acid	[M+Na]+	12.25	461.2282	461.2298	1.64	3.56	Terpenoids	Diterpenoids	Podocarpane diterpenoids
90	Pos+Neg	C[C@@]12CC[C@]3(C1)C(C[C@H](C4[C@@]3(CCC[C@]4(C)C(=O)O)C)OC(=O)C5=CC=CC=C5)C[C@@H]2O	(4S,5R,8S,9R,11aS,11bS)-5-(benzoyloxy)-8-hydroxy-4,9,11b-trimethyltetradecahydro-9,11a-methanocyclohepta[a]naphthalene-4-carboxylic acid	[M+H]+	11.38	441.2620	441.2636	1.55	3.51	Terpenoids	Diterpenoids	Podocarpane diterpenoids
91	Pos+Neg	C/C(=C\CO)/CC[C@@H]1C(=C)[C@@H]([C@H]([C@@H]2[C@@]1(CCC[C@]2(C)C(=O)O)C)OC(=O)C3=CC=CC=C3)O	(7S)-4-epi-7-hydroxyscoparic acid A	[M+H]+	10.06	457.2547	457.2585	3.76	8.23	Terpenoids	Diterpenoids	Labdane diterpenoids
92	Pos+Neg	COC1=CC2=C(C=C1)N(C(=O)[C@@H](O2)O[C@H]3[C@@H]([C@H]([C@@H]([C@H](O3)CO)O)O)O)OC	(S)-4,7-dimethoxy-2-(((2S,3R,4S,5S,6R)-3,4,5-trihydroxy-6-(hydroxymethyl)tetrahydro-2H-pyran-2-yl)oxy)-2H-benzo[b][1,4]oxazin-3(4H)-one	[M+NH4]+	5.49	405.1526	405.1504	2.23	5.51	Alkaloids	Anthranilic acid alkaloids	Quinoline alkaloids

93	Pos+Neg	COC1=CC2=C(C=C1)N(C(=O)O2)O	1-hydroxy-6-methoxy-2-benzoxazolin-2(3H)-one	[M+H]+	1.41	182.0437	182.0448	1.08	5.95	Alkaloids	Unknown	Unknown
94	Pos+Neg	C[C@]12CC[C@@H](C(C1CC[C@@]3(C2CC=C4[C@]3(CC[C@@]5(C4CC(C[C@H]5O)(C)C)C(=O)O)C)C)(C)C)O	22β-Hydroxy-oleanolsaeure	[M+H-H2O]+	14.85	455.3503	455.3520	1.67	3.67	Terpenoids	Triterpenoids	Oleanane triterpenoids
95	Pos+Neg	C1C(C(C(CC1(C(=O)O)O)OC(=O)C=CC2=CC(=C(C=C2)O)O)OC(=O)C=CC3=CC(=C(C=C3)O)O)O	3,4-bis({[3-(3,4-dihydroxyphenyl)prop-2-enoyl]oxy})-1,5-dihydroxycyclohexane-1-carboxylic acid	[M+H-H2O]+	6.98	499.1189	499.1235	4.59	9.19	Shikimates and Phenylpropanoids	Phenylpropanoids (C6-C3)	Cinnamic acids and derivatives
96	Pos+Neg	C1[C@H]([C@@H]([C@@H](C[C@]1(C(=O)O)O)OC(=O)/C=C/C2=CC(=C(C=C2)O)O)OC(=O)/C=C/C3=CC(=C(C=C3)O)O)O	3,4-di-O-caffeoylquinic acid	[M+H]+	4.48	517.1324	517.1341	1.65	3.19	Shikimates and Phenylpropanoids	Phenylpropanoids (C6-C3)	Cinnamic acids and derivatives
97	Pos+Neg	COC1=C(C=C(C=C1)C2=CC(=O)C3=C(O2)C=C(C=C3OC)OC)O	3'-hydroxy-5,7,4'-trimethoxyflavone	[M+H]+	10.55	329.1005	329.1020	1.46	4.45	Shikimates and Phenylpropanoids	Flavonoids	Flavones
98	Pos+Neg	CC(=CCC/C(=C/CC1=C(C=CC(=C1O)C2=COC3=CC(=CC(=C3C2=O)O)O)O)/C)C	3'-geranyl-5,7,2',5'-tetrahydroxyisoflavone	[M+H-H2O]+	3.32	405.1714	405.1696	1.75	4.32	Shikimates and Phenylpropanoids	Isoflavonoids	Isoflavones
99	Pos+Neg	COC1=C(OC2=CC(=CC(=C2C1=O)O)O)C3=CC=C(C=C3)O	3-methoxy apigenin	[M+H]+	4.82	301.0698	301.0707	0.86	2.87	Shikimates and Phenylpropanoids	Flavonoids	Flavonols
100	Pos+Neg	CC(C)C[C@H]1[C@@H]2[C@H](CC(=C)[C@@H]3C[C@@H]([C@@]([C@@H]3[C@H]2OC1=O)(CCl)O)O)OC(=O)C(=C)CO	diain	[M+H-H2O]+	2.38	425.1764	425.1725	3.86	9.07	Terpenoids	Sesquiterpenoids	Guaiane sesquiterpenoids
101	Pos+Neg	C[C@@]12CC[C@@]3([C@H]4CC=C5[C@H]([C@@]4(CC[C@]3([C@@H]1CC(CC2)(C)C)C)C)CC[C@H](C5(C)C)O)C	5(6)-gluten-3α-ol; glutinol; α-glutinol	[M+H]+	19.64	427.3918	427.3934	1.64	3.84	Terpenoids	Triterpenoids	Glutinane triterpenoids
102	Pos+Neg	COC1=C(C(=C2C(=C1)OC(=CC2=O)C3=CC(=C(C=C3)O)O)O)O	5,6,3',4'-tetrahydroxy-7-methoxyflavone; pedalitin	[M+H]+	6.44	317.0643	317.0656	1.28	4.03	Shikimates and Phenylpropanoids	Flavonoids	Flavones
103	Pos+Neg	COC1=CC2=C(C=C1)OC(=C(C2=O)OC)C3=C(C(=C(C=C3)O)O)O	5',6',4'-trihydroxy-3,6-dimethoxyflavone	[M+H]+	7.19	331.0801	331.0812	1.13	3.41	Shikimates and Phenylpropanoids	Flavonoids	Flavonols
104	Pos+Neg	COC1=C(C(=C2C=CC(=O)OC2=C1)OC)OC	5,6,7-trimethoxycoumarin; 5,6,7-trimethoxy-2H-chromen-2-one	[M+NH4]+	5.13	254.1010	254.1023	1.30	5.10	Shikimates and Phenylpropanoids	Coumarins	Simple coumarins
105	Pos	C1=CC=C(C(=C1)C2=CC(=O)C3=C(C=C(C=C3O2)O)O)O	5,7,2'-Trihydroxyflavone	[M+H]+	4.80	271.0588	271.0601	1.30	4.79	Shikimates and Phenylpropanoids	Flavonoids	Flavones
106	Pos+Neg	COC1=C(C=CC(=C1)C2=C(C(=O)C3=C(C=C(C=C3O2)O)O)OC4C(C(C(C(O4)CO)O)O)O)O	5,7-dihydroxy-2-(4-hydroxy-3-methoxyphenyl)-3-{[3,4,5-trihydroxy-6-(hydroxymethyl)oxan-2-yl]oxy}-4H-chromen-4-one	[M+H]+	4.69	479.1167	479.1184	1.70	3.55	Shikimates and Phenylpropanoids	Flavonoids	Flavonols

107	Pos+Neg	COC1=C(C=C2C(=C1)C=CC(=O)O2)O	Scopoletin	[M+H]+	4.54	193.0488	193.0495	0.73	3.81	Shikimates and Phenylpropanoids	Coumarins	Simple coumarins
108	Pos+Neg	C[C@H]1[C@@H]([C@H]([C@H]([C@@H](O1)O[C@@H]2[C@H]([C@@H](O[C@@H]([C@H]2OC(=O)/C=C/C3=CC(=C(C=C3)O)O)CO)OCCC4=CC(=C(C=C4)O)O)O)O)O)O)O	Acteoside	[M+H]+	4.39	625.2099	625.2127	2.80	4.47	Shikimates and Phenylpropanoids	Phenylethanoids (C6-C2), Phenylpropanoids (C6-C3)	Cinnamic acids and derivatives, Phenylethanoids
109	Pos+Neg	CC(=O)OC1=C(C=C(C(=C1)O)C2=CC(=O)C3=C(C(=C(C=C3O2)OC)OC)O)OC	arcapillin 4'-O-acetate	[M+H]+	4.95	403.1009	403.1024	1.46	3.62	Shikimates and Phenylpropanoids	Flavonoids	Flavones
110	Pos+Neg	CC(=C)[C@@H]1CC[C@]2([C@H]1[C@H]3CC[C@@H]4[C@]5(CC[C@@H](C([C@@H]5CC[C@]4([C@@]3(CC2)C)C)(C)C)O)C)C(=O)O	Betulinic acid	[M+H]+	14.25	457.3657	457.3676	1.92	4.20	Terpenoids	Triterpenoids	Lupane triterpenoids
111	Pos+Neg	C1=CC=C2C(=C1)NC(=O)C(O2)O	Blepharigenin	[M+H]+	4.96	166.0489	166.0499	0.97	5.84	Alkaloids	Anthranilic acid alkaloids	Unknown
112	Pos+Neg	C1=CC(=C(C=C1C=CC(=O)O)O)O	Caffeic acid	[M+H]+	4.29	181.0485	181.0495	1.03	5.72	Shikimates and Phenylpropanoids	Phenylpropanoids (C6-C3)	Cinnamic acids and derivatives
113	Pos+Neg	CC(=CCC1=C(C(=CC(=C1)/C=C/C(=O)O)C/C=C(\C)/CO)O)C	capillartemisin A	[M+K]+	3.58	355.1275	355.1306	3.12	8.78	Shikimates and Phenylpropanoids	Phenylpropanoids (C6-C3)	Cinnamic acids and derivatives
114	Pos+Neg	C1[C@H]([C@H]([C@@H](C[C@@]1(C(=O)O)O)OC(=O)C=CC2=CC(=C(C=C2)O)O)O)O	chlorogenic acid	[M+H]+	2.91	355.1008	355.1024	1.56	4.39	Shikimates and Phenylpropanoids	Phenylpropanoids (C6-C3)	Cinnamic acids and derivatives
115	Pos+Neg	C/C(=C\COC(=O)C)/C(=O)O[C@H]1CC(=C)[C@@H]2C[C@@H]([C@@]([C@@H]2[C@@H]3[C@@H]1C(=C)C(=O)O3)(CCl)O)O	chloroscoparin	[M+H]+	2.76	455.1505	455.1467	3.78	8.30	Terpenoids	Sesquiterpenoids	Guaiane sesquiterpenoids
116	Pos+Neg	COC1=C(C=CC(=C1)C2=CC(=O)C3=C(C(=C(C=C3O2)OC)OC)O)O	cirsilineol	[M+H]+	8.73	345.0953	345.0969	1.58	4.58	Shikimates and Phenylpropanoids	Flavonoids	Flavones
117	Pos+Neg	CCCCC/C=C/C1=C([C@H]2[C@]3(C[C@@H](C(O2)(C)C)O)[C@H]([C@@H]1O)O3)CO	cytosporin D	[M+H-H2O]+	9.27	321.2048	321.2060	1.24	3.85	Polyketides	Chromanes	Cytosporins
118	Pos+Neg	CCCCC/C=C/C1=C([C@H]2[C@](C[C@@H](C(O2)(C)C)O)([C@H]3[C@@H]1OC(=O)O3)O)CO	cytosporin E	[M+H-H2O]+	7.04	365.1923	365.1959	3.56	9.76	Polyketides	Chromanes	Cytosporins
119	Pos+Neg	COC1=C(C=C(C=C1)C2=C(C(=O)C3=C(C=C(C=C3O2)O)O)O[C@H]4[C@@H]([C@H]([C@@H]([C@H](O4)COC(=O)C=CC5=CC=C(C=C5)O)O)O)O)OC	dillenetin 3-O-(6"-O-p-coumaroyl)-β-D-glucopyranoside	[M+H]+	6.31	639.1691	639.1708	1.73	2.71	Shikimates and Phenylpropanoids	Flavonoids	Flavonols
120	Pos+Neg	C[C@]12CC[C@@H]([C@@](C1CC[C@@]3(C2CC=C4[C@]3(CC[C@@]5(C4CC(CC5)(C)C)C(=O)O)C)C)(C)C(=O)O)O	gypsogenic acid	[M+K]+	5.67	525.3019	525.2977	4.22	8.03	Terpenoids	Triterpenoids	Oleanane triterpenoids
121	Pos+Neg	COC1=C(C=C(C=C1)C2=CC(=O)C3=C(C(=C(C(=C3O2)OC)O)OC)O)OC	hymenoxin; 5,7-dihydroxy-6,8,3',4'-tetramethoxyflavone	[M+H]+	8.44	375.1062	375.1074	1.24	3.32	Shikimates and Phenylpropanoids	Flavonoids	Flavones
122	Pos+Neg	CC1[C@@H](CCC2(C1C3=CCC4C5(CCC(=O)C(C5CCC4([C@@]3(CC2)C)C)(C)C)C)C)C(=O)O	Ifflaionsaeure	[M+H-H2O]+	13.76	437.3397	437.3414	1.71	3.90	Terpenoids	Triterpenoids	Lupane triterpenoids, Ursane and Taraxastane triterpenoids

123	Pos+Neg	COC1=C(C=CC(=C1)C2=C(C(=O)C3=C(C=C(C=C3O2)O)O)O[C@H]4[C@@H]([C@H]([C@H]([C@H](O4)CO)O)O)O)O	isorhamnetin 3-β-D-galactopyranoside	[M+H-H2O]+	6.40	461.1062	461.1078	1.64	3.55	Shikimates and Phenylpropanoids	Flavonoids	Flavonols
124	Pos+Neg	C1[C@@H]([C@@H]([C@H]([C@@H](O1)C2=C(C(=C3C(=C2O)C(=O)C=C(O3)C4=CC=C(C=C4)O)[C@H]5[C@@H]([C@H]([C@@H]([C@H](O5)CO)O)O)O)O)O)O)O	Isoschaftoside	[M+H]+	3.71	565.1527	565.1552	2.48	4.39	Shikimates and Phenylpropanoids	Flavonoids	Flavones
125	Pos+Neg	C1=CC(=CC=C1C2=CC(=O)C3=C(O2)C=C(C(=C3O)[C@H]4[C@@H]([C@H]([C@@H]([C@H](O4)CO)O)O)O)O)O	isovitexin	[M+H]+	4.80	433.1117	433.1129	1.22	2.82	Shikimates and Phenylpropanoids	Flavonoids	Flavones
126	Pos+Neg	COC1=CC(=C2C(=C1)OC(=C(C2=O)OC)C3=CC=C(C=C3)O)O	kumatakenin	[M+H]+	8.28	315.0850	315.0863	1.31	4.17	Shikimates and Phenylpropanoids	Flavonoids	Flavonols
127	Pos	C1=CC(=C(C=C1C2=CC(=O)C3=C(O2)C(=C(C=C3O)O)[C@H]4[C@@H]([C@H]([C@@H]([C@H](O4)CO)O)O)O)O)O	Luteolin-8-C-glucoside	[M+H]+	4.54	449.1062	449.1078	1.64	3.65	Shikimates and Phenylpropanoids	Flavonoids	Flavones
128	Pos+Neg	C1=CC(=C(C=C1C2=CC(=O)C3=C(C(=C(C=C3O2)O[C@H]4[C@@H]([C@H]([C@@H]([C@H](O4)CO)O)O)O)[C@H]5[C@@H]([C@H]([C@@H]([C@H](O5)CO)O)O)O)O)O)O	lutonarin	[M+H]+	3.11	611.1579	611.1607	2.76	4.52	Shikimates and Phenylpropanoids	Flavonoids	Flavones
129	Pos+Neg	COC1=C(C=CC(=C1)C[C@H]2COC(=O)[C@@H]2CC3=CC(=C(C=C3)O)OC)O	matairesinol	[M+H-H2O]+	4.77	341.1367	341.1383	1.65	4.84	Shikimates and Phenylpropanoids	Lignans	Dibenzylbutyrolactone lignans
130	Pos+Neg	CCOC(=O)[C@@H]1[C@H]([C@@H]([C@H]([C@@H](O1)O[C@H]2CC[C@]3(C(C2(C)C)CC[C@@]4(C3CC=C5[C@]4(CC[C@@]6(C5CC(CC6)(C)C)C(=O)O)C)C)C)O)O[C@H]7[C@@H]([C@H]([C@@H](CO7)O)O)O)O	momordin Ic 6'-ethyl ester	[M+Na]+	16.08	815.4475	815.4552	7.71	9.46	Terpenoids	Triterpenoids	Oleanane triterpenoids
131	Pos+Neg	C1=CC(=CC=C1/C=C/C(=O)O)O	p-Coumaric Acid	[M+H]+	4.23	165.0538	165.0546	0.82	4.97	Shikimates and Phenylpropanoids	Phenylpropanoids (C6-C3)	Cinnamic acids and derivatives
132	Pos+Neg	C[C@H]1[C@@H]([C@H]([C@H]([C@@H](O1)OC[C@@H]2[C@H]([C@@H]([C@H]([C@@H](O2)OC3=C(C(=C4C(=C3)OC(=CC4=O)C5=CC=C(C=C5)OC)O)OC)O)O)O)O)O)O	pectolinarin	[M+H]+	5.99	623.1941	623.1970	2.95	4.73	Shikimates and Phenylpropanoids	Flavonoids	Flavones
133	Pos+Neg	C[C@H]1[C@@H]([C@H]([C@H]([C@@H](O1)OC[C@@H]2[C@H]([C@@H]([C@H]([C@@H](O2)OCCC3=CC(=C(C=C3)O)O)O)O[C@H]4[C@@H]([C@@H]([C@H]([C@@H](O4)C)O)O)O)OC(=O)/C=C/C5=CC(=C(C=C5)O)O)O)O)O	Poliummoside	[M+NH4]+	4.29	788.2944	788.2972	2.75	3.49	Shikimates and Phenylpropanoids	Phenylethanoids (C6-C2), Phenylpropanoids (C6-C3)	Cinnamic acids and derivatives, Phenylethanoids
134	Pos+Neg	C[C@H]1[C@@H]([C@H]([C@H]([C@@H](O1)OC[C@@H]2[C@H]([C@@H]([C@H]([C@@H](O2)OC3=CC(=C4C(=C3)OC(=CC4=O)C5=CC(=C(C=C5)O)O)O)O)O)O)O)O)O	scolymoside	[M+H]+	3.43	595.1634	595.1657	2.35	3.94	Shikimates and Phenylpropanoids	Flavonoids	Flavones
135	Pos+Neg	C[C@]12CC[C@]3(C1)[C@@H](C[C@H]([C@@H]4[C@@]3(CCC[C@]4(C)C(=O)O)C)OC(=O)C5=CC=CC=C5)CC2=O	scopadulcic acid A	[M+H]+	10.91	455.2411	455.2428	1.71	3.77	Terpenoids	Diterpenoids	Podocarpane diterpenoids,

												Tetracyclic diterpenoids
136	Pos+Neg	CC(=O)CCC1C(=C)C[C@H](C2[C@@]1(CCC[C@@]2(C)C(=O)O)C)OC(=O)C3=CC=CC=C3	scoparic acid B	[M+NH4]+	11.13	430.2571	430.2588	1.70	3.94	Terpenoids	Diterpenoids	Norlabdane diterpenoids
137	Pos+Neg	CC(=O)CC[C@@H]1C(=C)C[C@H]([C@@H]2[C@@]1(CCC[C@@]2(C)CO)C)OC(=O)C3=CC=CC=C3	scoparicol A	[M+H]+	12.73	399.2511	399.2530	1.89	4.72	Terpenoids	Diterpenoids	Norlabdane diterpenoids
138	Pos+Neg	C[C@@]1(CCC[C@@]2([C@@H]1[C@H](CC(=C)[C@@H]2CCC(=C)C=O)OC(=O)C3=CC=CC=C3)C)CO	scoparicol B	[M+H]+	12.77	411.2493	411.2530	3.69	8.96	Terpenoids	Diterpenoids	Labdane diterpenoids
139	Pos+Neg	C[C@]1(CCC[C@]2([C@H]1[C@@H](CC(=C)[C@H]2CCC(=C)[C@H](CO)O)OC(=O)C3=CC=CC=C3)C)CO	scoparicol C	[M+H-H2O]+	10.89	425.2670	425.2686	1.64	3.85	Terpenoids	Diterpenoids	Labdane diterpenoids
140	Pos+Neg	COC1=C(C=C2C(=C1)C=CC(=O)O2)OC	Scoparone	[M+NH4]+	4.90	224.0907	224.0917	1.03	4.60	Shikimates and Phenylpropanoids	Coumarins	Simple coumarins
141	Pos+Neg	COC1=C(C=C2C(=C1O)C(=O)C=C(O2)C3=CC=C(C=C3)O)O[C@H]4[C@@H]([C@H]([C@@H]([C@H](O4)CO)O)O)O	tectoridin	[M+H]+	4.82	463.1221	463.1235	1.39	3.00	Shikimates and Phenylpropanoids	Flavonoids	Flavones
142	Pos+Neg	C1=CC(=CC=C1C2=CC(=O)C3=C(C(=C(C(=C3O2)C4C(C(C(C(O4)CO)O)O)O)O)C5C(C(C(C(O5)CO)O)O)O)O)O	Violantin	[M+H-H2O]+	3.43	577.1529	577.1552	2.28	3.95	Shikimates and Phenylpropanoids	Flavonoids	Flavones
143	Pos+Neg	C1=CC(=CC=C1C2=CC(=O)C3=C(O2)C(=C(C=C3O)O)C4C(C(C(C(O4)CO)O)O)O)O	Vitexin	[M+H]+	4.33	433.1116	433.1129	1.32	3.05	Shikimates and Phenylpropanoids	Flavonoids	Flavones
144	Neg	C/C(=C/CC1=C(C(=CC(=C1)/C=C/C(=O)O)C/C=C(/C)\COC(=O)C)O)/CO	cis-scopa-trans-coumaricin	[M-H]-	9.06	373.1655	373.1657	0.16	0.44	Shikimates and Phenylpropanoids	Phenylpropanoids (C6-C3)	Cinnamic acids and derivatives
145	Neg	C/C=C(\CO)/C(=O)O[C@H]1CC(=C)[C@@H]2C[C@@H]([C@@]([C@@H]2[C@@H]3[C@@H]1C(=C)C(=O)O3)(CCl)O)O	(1R,3S,4S,5S,6S,7R,8S)-4β-(chloromethyl)-3β,4α-dihydroxy-8α-(sarracenoyloxy)-1αH,5αH,6βH,7αH-guai-10(14),11(13)-dien-6,12-olide	[M+Cl]-	4.16	447.0962	447.0978	1.58	3.54	Terpenoids	Sesquiterpenoids	Guaiane sesquiterpenoids
146	Neg	C/C=C/C=C/C#CCCCO[C@H]1[C@@H]([C@H]([C@@H]([C@H](O1)CO)O)O)O	6E,8E-decadien-4-ynol 1-O-β-D-glucopyranoside	[M+Br]-	5.11	391.0728	391.0756	2.82	7.22	Fatty acids	Unknown	Unknown
147	Neg	C[C@@]1(CC[C@@H](C([C@H]1CCC(C)(C=C)O)(C)C)O)O	2-(3-hydroxy-3-methylpent-4-en-1-yl)-1,3,3-trimethylcyclohexane-1,4-diol; 3,7,10-trihydroxy-6,11-cyclofarnes-1-ene	[M+FA]-	7.50	301.2037	301.2015	2.20	7.30	Terpenoids	Sesquiterpenoids	Cyclofarnesane sesquiterpenoids
148	Neg	C[C@@]12CC[C@]3(C1)[C@@H](C[C@H]([C@@H]4[C@@]3(CCC[C@]4(C)C=O)C)OC(=O)C5=CC=CC=C5)C[C@@H]2O	dulcinodal	[M+Br]-	4.91	503.1808	503.1797	1.10	2.19	Terpenoids	Diterpenoids	Podocarpane diterpenoids

149	Neg	C[C@@]12CC[C@]3(CCC(C=C3[C@H]1CC[C@H]4[C@]2(CC[C@@H]5[C@@]4(CC[C@@H](C5(C)C)O[C@H]6[C@@H]([C@H]([C@@H]([C@H](O6)C(=O)O)O)O[C@H]7[C@@H]([C@H]([C@@H](CO7)O)O)O)O)C)C)(C)C)C(=O)O	(2S,3S,4S,5R,6R)-6-(((3S,4aR,6aR,6bR,8aS,12bS,14aR,14bR)-8a-carboxy-4,4,6a,6b,11,11,14b-heptamethyl-1,2,3,4,4a,5,6,6a,6b,7,8,8a,9,10,11,12b,13,14,14a,14b-icosahydropicen-3-yl)oxy)-3,5-dihydroxy-4-(((2S,3R,4S,5R)-3,4,5-trihydroxytetrahydro-2H-pyran-2-yl)oxy)tetrahydro-2H-pyran-2-carboxylic acid	[M+CF3CO2]-	10.25	877.4213	877.4197	1.57	1.79	Terpenoids	Triterpenoids	Oleanane triterpenoids
150	Neg	C[C@]12CC[C@@H](C(C1CC[C@@]3(C2CC=C4[C@]3(CC[C@@]5(C4CC(CC5)(C)C)C(=O)O[C@H]6[C@@H]([C@H]([C@@H]([C@H](O6)CO)O)O)O)C)C)(C)C)O[C@H]7[C@@H]([C@H]([C@@H]([C@H](O7)C(=O)OC)O)O)O[C@H]8[C@@H]([C@H]([C@@H](CO8)O)O)O)O	momordin IIc 6′-methyl ester	[M+Cl]-	7.19	975.4730	975.4721	0.93	0.96	Terpenoids	Triterpenoids	Oleanane triterpenoids
151	Neg	C[C@]12CC[C@@H](C(C1CC[C@@]3(C2CCC4=C5CC(CC[C@@]5(CC[C@]43C)C(=O)O)(C)C)C)(C)C)O[C@H]6[C@@H]([C@H]([C@@H]([C@H](O6)CO)O)O)O	(3β)-3-(β-D-glucopyranosyloxy)olean-13(18)-en-28-oic acid	[M+Acetate]-	6.95	677.4292	677.4265	2.73	4.02	Terpenoids	Triterpenoids	Oleanane triterpenoids
152	Neg	C[C@]12CC[C@]3(C1)[C@@H](C[C@H]([C@@H]4[C@@]3(CCC[C@]4(C)C=O)C)OC(=O)C5=CC=CC=C5)CC2=O	dulcinodal-13-one	[M+Acetate]-	11.88	481.2603	481.2590	1.29	2.67	Terpenoids	Diterpenoids	Podocarpane diterpenoids
153	Neg	C[C@]12CCC[C@@](C1C(CC(=C)C2CCO)O)(C)C(=O)O	scoparic acid D	[M+FA]-	7.19	327.1830	327.1808	2.24	6.83	Terpenoids	Diterpenoids	Norlabdane diterpenoids
154	Neg	C[C@H]1[C@@H]([C@H]([C@H]([C@@H](O1)O[C@@H]2[C@H]([C@@H](O[C@@H]([C@H]2OC(=O)/C=C/C3=CC(=C(C=C3)O)O)CO[C@@H]4[C@@H]([C@](CO4)(CO)O)O)OCCC5=CC(=C(C=C5)OC)OC)O)O)O)O	beta-D-Glucopyranoside, 2-(3,4-dimethoxyphenyl)ethyl 3-O-(6-deoxy-alpha-L-mannopyranosyl)-4-O-[(2E)-3-(3,4-dihydroxyphenyl)-1-oxo-2-propen-1-yl]-6-O-[(2S,3R,4R)-tetrahydro-3,4-dihydroxy-4-(hydroxymethyl)-2-furanyl]-	[M-H]-	4.71	783.2777	783.2717	6.00	7.66	Shikimates and Phenylpropanoids	Phenylethanoids (C6-C2), Phenylpropanoids (C6-C3)	Cinnamic acids and derivatives, Phenylethanoids

155	Neg	C[C@H]1[C@@H]([C@H]([C@H]([C@@H](O1)OC[C@@H]2[C@H]([C@@H]([C@H]([C@@H](O2)OC3=C(C(=C4C(=C3)OC(=CC4=O)C5=CC=C(C=C5)O)O)O)O)O)O)OC(=O)C)OC(=O)C)O	5,6,4'-trihydroxyflavone 7-O-α-L-2,3-di-O-acetylrhamnopyranosyl-(1->6)-β-D-glucopyranoside	[M+Cl]-	5.21	713.1419	713.1485	6.60	9.25	Shikimates and Phenylpropanoids	Flavonoids	Flavones
156	Neg	C[C@H]1[C@@H]([C@H]([C@H]([C@H](O1)OC[C@@H]2[C@H]([C@@H]([C@H]([C@@H](O2)OC3=CC4=C(C=C3)C(=O)C=C(O4)C5=CC=C(C=C5)OC)O)O)O)O)O)O	linarin	[M+CF3CO2]-	5.99	689.1750	689.1693	5.67	8.23	Shikimates and Phenylpropanoids	Flavonoids	Flavones
157	Neg	C[C@H]1[C@@H]([C@H]([C@H](C(O1)O[C@H]2[C@@H]([C@H](O[C@H]([C@@H]2O)OCCC3=CC(=C(C=C3)O)O)COC(=O)/C=C/C4=CC(=C(C=C4)O)O)O)O)O)O	[(2R,3R,4S,5R,6R)-6-[2-(3,4-dihydroxyphenyl)ethoxy]-3,5-dihydroxy-4-[(3R,4R,5R,6S)-3,4,5-trihydroxy-6-methyloxan-2-yl]oxyoxan-2-yl]methyl (E)-3-(3,4-dihydroxyphenyl)prop-2-enoate	[M-H]-	4.42	623.2040	623.1981	5.86	9.40	Shikimates and Phenylpropanoids	Phenylethanoids (C6-C2), Phenylpropanoids (C6-C3)	Cinnamic acids and derivatives, Phenylethanoids
158	Neg	C[C@H]1[C@@H]2C[C@@H]([C@]3([C@]45CC[C@@H]([C@]4(CC[C@@]3([C@]2(CC(=O)C1=O)C)C(=O)C5=O)C)[C@H](C)CCC=C(C)C)O)O	(4S,5S,7S,8R,9S,10S,13R,14S,17R)-7,8-dihydroxy-4,10,13-trimethyl-17-((R)-6-methylhept-5-en-2-yl)dodecahydro-2H-9,14-ethanocyclopenta[a]phenanthrene-2,3,18,19(4H)-tetraone	[M+Br]-	3.01	577.2176	577.2165	1.12	1.95	Terpenoids	Unknown	Unknown
159	Neg	C[C@H]1[C@H]2CCC(=C[C@H]2OC1=O)C	(3S,3aR,7aS)-wine lactone	[M+FA]-	3.51	211.0985	211.0970	1.47	6.94	Terpenoids	Sesquiterpenoids	Germacrane sesquiterpenoids
160	Neg	C[C@H]1C2=C(C(=CC(=C2C)OC)O)C(=O)O[C@]1(CO)O	(3R,4S)-3,8-dihydroxy-3-hydroxymethyl-6-methoxy-4,5-dimethylisochroman-1-one	[M+Acetate]-	3.09	327.1103	327.1080	2.31	7.05	Shikimates and Phenylpropanoids	Coumarins	Isocoumarins
161	Neg	C=CCCCCC/C=C\C(C#CC#CC(=O)CCO)O	1,8-dihydroxy-heptadeca-9c,16-diene-4,6-diyn-3-one	[M+Acetate]-	8.71	333.1703	333.1702	0.10	0.30	Fatty acids	Fatty acyls	Fatty alcohols
162	Neg	C1=CC(=C(C=C1C(=O)O)O)O	protocatechic acid	[M-H]-	1.90	153.0196	153.0193	0.27	1.75	Shikimates and Phenylpropanoids	Phenolic acids (C6-C1)	Simple phenolic acids
163	Neg	C1=CC=C(C=C1)C=C(C(=O)O)OC2C(C(C(C(O2)COC(=O)C=CC3=CC=C(C=C3)O)O)O)O	3-phenyl-2-[(2S,3R,4S,5S,6R)-3,4,5-trihydroxy-6-[[(E)-3-(4-hydroxyphenyl)prop-2-	[M-H]-	5.45	471.1312	471.1297	1.53	3.25	Shikimates and Phenylpropanoids	Phenylpropanoids (C6-C3)	Cinnamic acids and derivatives

			enoyl]oxymethyl]oxan-2-yl]oxyprop-2-enoic acid								
164	Neg	CC(=CCC/C(=C/CC/C(=C/CC/C=C(/CC/C=C(/CCC=C(C)C)\C)\C)/C)/C)C	Squalene	[M+Br]-	11.79	489.3101	489.3096	0.51	1.05	Terpenoids	Triterpenoids	Acyclic triterpenoids
165	Neg	CC(=CCC/C(=C/CC1=C(C=CC(=C1)C2=COC3=CC(=CC(=C3C2=O)O)O)O)/C)C	Myrsininone A	[M+Br]-	2.59	485.0944	485.0964	1.96	4.04	Shikimates and Phenylpropanoids	Isoflavonoids	Isoflavones
166	Neg	CC(=CCC1=CC(=C(C(=C1O)C=O)CCCCCC=C)O)C	2-(hept-6-en-1-yl)-3,6-dihydroxy-5-(3-methylbut-2-en-1-yl)benzaldehyde	[M+Acetate]-	10.71	361.2017	361.2015	0.20	0.55	Terpenoids	Meroterpenoids	Tetraketide meroterpenoids
167	Neg	CC1=C(C=C(C2=C1[C@](C(=C)OC2=O)(C)O)O)OC	4,8-dihydroxy-6-methoxy-4,5-dimethyl-3-methyleneisochroman-1-one	[M+FA]-	2.72	295.0838	295.0818	2.02	6.85	Shikimates and Phenylpropanoids	Coumarins	Isocoumarins
168	Neg	CC1=CCC[C@@]2([C@@H]1C[C@H](CC2)C(=C)COC(=O)C)C	eutyscoparin A	[M+FA]-	9.98	307.1931	307.1909	2.16	7.05	Terpenoids	Sesquiterpenoids	Eudesmane sesquiterpenoids
169	Neg	CC1C(C(C(C(O1)OCC2C(C(C(C(O2)OC3=C(C(=C4C(=C3)OC(=CC4=O)C5=CC=C(C=C5)OC)O)OC)O)O)O)O)O)O	Pectolinarin	[M-H]-	5.99	621.1872	621.1825	4.71	7.58	Shikimates and Phenylpropanoids	Flavonoids	Flavones
170	Neg	CC1C(CC2C1COC(C2C(=O)OC)O)OC(=O)C3=COC(C(C3CC=O)C=C)O[C@H]4[C@@H]([C@H]([C@@H]([C@@H](O4)CO)O)O)O	laciniatoside I	[M+Acetate]-	3.49	645.2420	645.2395	2.54	3.94	Terpenoids	Monoterpenoids	Iridoids monoterpenoids, Secoiridoid monoterpenoids
171	Neg	CCC(CC[C@@H](C)[C@H]1CC[C@@H]2[C@@]1(CC[C@H]3[C@H]2CC=C4[C@@]3(CC[C@@H](C4)O)C)C)C(C)C	β-sitosterol	[M+Br]-	6.45	493.3043	493.3045	0.20	0.41	Terpenoids	Steroids	Stigmastane steroids
172	Neg	CCCCCCCC1=C([C@H]2[C@]3(C[C@@H](C(O2)(C)C)O)[C@H]([C@@H]1O)O3)CO	cytosporin X	[M+FA]-	5.70	385.2254	385.2226	2.77	7.19	Polyketides	Chromanes	Cytosporins
173	Neg	CCCCCCCCCCCCC/C=C(\CCCCCCCCCCCCC)/C=O	(E)-2-Tridecyl-hexadec-2-enal	[M+Cl]-	13.96	455.4014	455.4020	0.62	1.35	Fatty acids	Fatty acyls	Hydrocarbons
174	Neg	CCCCCCCCCCCCCCCC(=O)OC[C@H](COP(=O)(O)OC1[C@@H]([C@H](C([C@H]([C@H]1O)O)O)O)O)O	1-Hexadecanoyl-sn-glycero-3-phospho-(1'-myo-inositol)	[M-H]-	11.22	571.2927	571.2889	3.81	6.67	Fatty acids	Glycerophospholipids	Glycerophosphoinositols
175	Neg	COC1=C(C(=C2C(=O)CC(OC2=C1)C3=CC=C(C=C3)O[C@H]4[C@@H]([C@H]([C@@H]([C@H](O4)CO)O)O)O)O)OC	5-hydroxy-6,7-dimethoxyflavone 4'-O-D-glucose	[M+FA]-	3.68	523.1494	523.1452	4.23	8.09	Shikimates and Phenylpropanoids	Flavonoids	Flavanones
176	Neg	COC1=C(C=C2C(=C1O)C(=O)C=C(O2)C3=CC=C(C=C3)O)OC4C(C(C(C(O4)CO)O)O)O	Hispiduloside	[M-H]-	5.79	461.1119	461.1089	2.96	6.43	Shikimates and Phenylpropanoids	Flavonoids	Flavones

177	Neg	COC1=C(C=CC(=C1)CC=C)O	eugenol	[M+FA]-	5.09	209.0803	209.0814	1.08	5.19	Shikimates and Phenylpropanoids	Phenylpropanoids (C6-C3)	Cinnamic acids and derivatives
178	Neg	COC1=C(C=CC(=C1)CC=C)O[C@H]2[C@@H]([C@H]([C@@H]([C@H](O2)CO)O)O)O	citrusin C	[M+FA]-	5.24	371.1371	371.1342	2.89	7.79	Shikimates and Phenylpropanoids	Phenylpropanoids (C6-C3)	Cinnamic acids and derivatives
179	Neg	COC1=C(C2=C(C=C1O)OC(=C(C2=O)OC)C3=CC=C(C=C3)O)O	3,6-Dimethoxy Kaempferol	[M-H]-	7.19	329.0686	329.0667	1.92	5.84	Shikimates and Phenylpropanoids	Flavonoids	Flavonols
180	Neg	COC1=CC(=C2C(=C1)OC(=CC2=O)C3=CC(=C(C=C3)O)OC)O	Velutin	[M-H]-	9.21	313.0733	313.0718	1.54	4.91	Shikimates and Phenylpropanoids	Flavonoids	Flavones
181	Neg	COC1=CC(=CC(=C1O)O)C2=CC(=O)C3=C(O2)C=C(C(=C3O)OC)O	2-(3,4-dihydroxy-5-methoxyphenyl)-5,7-dihydroxy-6-methoxy-4H-chromen-4-one	[M-H]-	6.39	345.0638	345.0616	2.21	6.40	Shikimates and Phenylpropanoids	Flavonoids	Flavones
182	Neg	COC1=CC(=CC(=C1O)OC)[C@H]2C3CO[C@@H](C3CO2)C4=CC(=C(C(=C4)OC)O[C@H]5[C@@H]([C@H]([C@@H]([C@H](O5)CO)O)O)O[C@H]6[C@@H]([C@](CO6)(CO)O)O)OC	(2R,3S,4S,5R,6S)-5-[(2S,3R,4R)-3,4-dihydroxy-4-(hydroxymethyl)oxolan-2-yl]oxy-6-[4-[(3R,6S)-3-(4-hydroxy-3,5-dimethoxyphenyl)-1,3,3a,4,6,6a-hexahydrofuro[3,4-c]furan-6-yl]-2,6-dimethoxyphenoxy]-2-(hydroxymethyl)oxane-3,4-diol	[M+FA]-	4.69	757.2624	757.2555	6.90	9.11	Shikimates and Phenylpropanoids	Lignans	Furofuranoid lignans
183	Neg	COC1=CC2=C(C=C1)N(C(=O)C(O2)OC3C(C(C(C(O3)CO)O)O)O)OC	4,7-dimethoxy-2-{[3,4,5-trihydroxy-6-(hydroxymethyl)oxan-2-yl]oxy}-3,4-dihydro-2H-1,4-benzoxazin-3-one	[M-H]-	3.84	386.1117	386.1093	2.43	6.29	Alkaloids	Anthranilic acid alkaloids	Quinoline alkaloids
184	Neg	COC1=CC2=C(C=C1)NC(=O)O2	Mboa	[M-H]-	4.95	164.0358	164.0353	0.48	2.94	Alkaloids	Tryptophan alkaloids	Unknown

Table S2. Compounds identified from SD that satisfied the criteria for Lipinski's rule, Caco–2 permeability, and oral bioavailability

Compound IDs	Molecular weight	Lipinski	Caco–2	Oral bioavailability
ASD12	149.05	0	−4.89551	0.241302
ASD17	298.08	0	−4.84855	0.001388
ASD19	229.13	0	−4.90105	0.003498
ASD20	260.18	0	−4.84619	0.000168
ASD21	424.37	0	−4.56877	0.095617
ASD22	258.16	0	−4.80495	0.144484
ASD27	195.05	0	−4.54815	0.232829
ASD32	196.07	0	−5.0115	0.190649
ASD38	388.12	0	−4.98368	0.078372
ASD46	344.09	0	−5.11778	0.216279
ASD51	220.18	0	−4.53559	0.249689
ASD59	222.09	0	−4.62207	0.000329
ASD68	468.36	0	−4.97817	0.023552
ASD70	432.21	0	−4.68109	0.002572
ASD71	238.19	0	−4.71291	0.215444
ASD79	186.13	0	−5.01658	0.276075

ASD80	430.2	0	−4.75843	0.000719
ASD85	294.18	0	−4.73697	0.114088
ASD87	332.2	0	−4.79625	0.109602
ASD88	426.28	0	−4.97624	0.266589
ASD107	192.04	0	−4.70673	0.295545
ASD109	402.1	0	−5.14884	0.23773
ASD111	165.04	0	−4.23059	0.000758
ASD126	314.08	0	−4.94947	0.183107
ASD140	206.06	0	−4.51939	0.15386
ASD161	274.16	0	−4.72589	0.080803
ASD164	410.39	0	−4.59253	0.000805
ASD168	262.19	0	−4.55645	0.13454
ASD171	414.39	0	−5.0119	0.079894
ASD179	330.07	0	−5.05242	0.152631

Table S3. Molecular docking results of ASD-derived compounds against TNF-α and IL6

Compound	Docking score (kcal/mol)	
	TNF–α	IL6
ASD12	– 4.965	– 4.896
ASD17	– 6.242	– 6.494
ASD19	– 4.49	– 4.822
ASD20	– 4.686	– 5
ASD21	– 7.093	– 7.682
ASD22	– 4.383	– 5.038
ASD27	– 4.437	– 4.623
ASD32	– 4.834	– 4.801
ASD38	– 5.787	– 7.843
ASD46	– 5.7	– 6.287
ASD51	– 5.547	– 4.941
ASD59	– 5.929	– 6.337
ASD68	– 7.003	– 7.000
ASD70	– 5.115	– 5.863
ASD71	– 5.667	– 5.101
ASD79	– 4.491	– 5.398

ASD80	−5.43	−6.243
ASD85	−6.198	−5.52
ASD87	−5.933	−6.181
ASD88	−6.39	−7.055
ASD107	−5.015	−5.182
ASD109	−5.78	−6.759
ASD111	−4.961	−4.76
ASD126	−5.527	−6.004
ASD140	−5.019	−5.147
ASD161	−4.236	−4.707
ASD164	−5.424	−5.87
ASD168	−5.785	−5.702
ASD171	−7.546	−6.654
ASD179	−5.79	−7.365

Table S4. Hydrogen bond occupancy between top ligands and main amino acids of TNF-α.

Complex protein and	Donor	Acceptor	Occupancy (%)
	LIG158-Side	TYR59-Side	94.87%
ASD21	TYR59-Side	LIG158-Side	29.74%
	TYR59-Side	LIG158-Main	1.00%
	LIG158-Side	TYR59-Side	46.41%
	TYR59-Side	LIG158-Side	33.77%
	LIG158-Main	TYR59-Side	4.01%
	TYR59-Side	LIG158-Main	2.58%
ASD68	LIG158-Side	TYR151-Side	36.10%
	TYR151-Side	LIG158-Main	23.90%
	TYR151-Side	LIG158-Side	13.57%
	LIG158-Main	TYR151-Side	4.52%
	TYR59-Side	LIG158-Side	117.23%
	LIG158-Side	TYR59-Side	89.85%
	LIG158-Side	TYR151-Side	48.59%
307*	TYR151-Side	LIG158-Side	27.23%
	TYR119-Side	LIG158-Side	58.82%
	LIG158-Side	TYR119-Side	56.40%

	LIG158-Side	TYR119-Main	9.66%
	TYR119-Main	LIG158-Side	2.90%

*Control ligand

Table S5. Total hydrogen bond occupancy (over 70 %) between top ligands and main amino acids of TNF-α.

	TYR59	TYR119	TYR151
ASD21	125.61%		
ASD68	86.77%		78.09%
307*	207.08%	127.78%	75.82%

*Control ligand

Table S6. Hydrogen bond occupancy between top ligands and main amino acids of IL6.

**Complex protein and**	**Donor**	**Acceptor**	**Occupancy (%)**
	LIG185-Side	GLN175-Side	57.99%
	GLN175-Side	LIG185-Side	15.47%
**ASD21**	LIG185-Side	GLN175-Main	5.62%
	GLN175-Side	LIG185-Main	2.70%
	GLN175-Main	LIG185-Side	2.06%

Hydrogen-bond occupancy values represent the cumulative occupancy of all donor–acceptor pairs involving the same ligand and amino acid residue. Therefore, residue-level cumulative occupancy may exceed 100% when multiple hydrogen bonds are formed.

Figure legend

Fig. S1. Superimposition of redocked (red) and the co-crystalized ligand (light blue) in the protein 2AZ5 for the validation of docking protocol

Fig. S2. 2D interaction between the co–crystalized ligand and protein TNF-α

Fig. S3 Structural superimposition of the top-ranked docked compounds within the binding site of TNF-α

Fig. S4 Structural superimposition of the top-ranked docked compounds within the binding site of IL6

Fig. S5. PCA scatter plots of PC1 versus PC2 for TNF-α and IL-6 in the apoprotein and ligand-bound states. TNF-α systems include the apoprotein (T0), ASD21-bound form (T1), ASD68-bound form (T2), and control-bound form (T3), whereas IL-6 systems comprise the apoprotein (L0), ASD21-bound form (L1), and ASD179-bound form (L2).

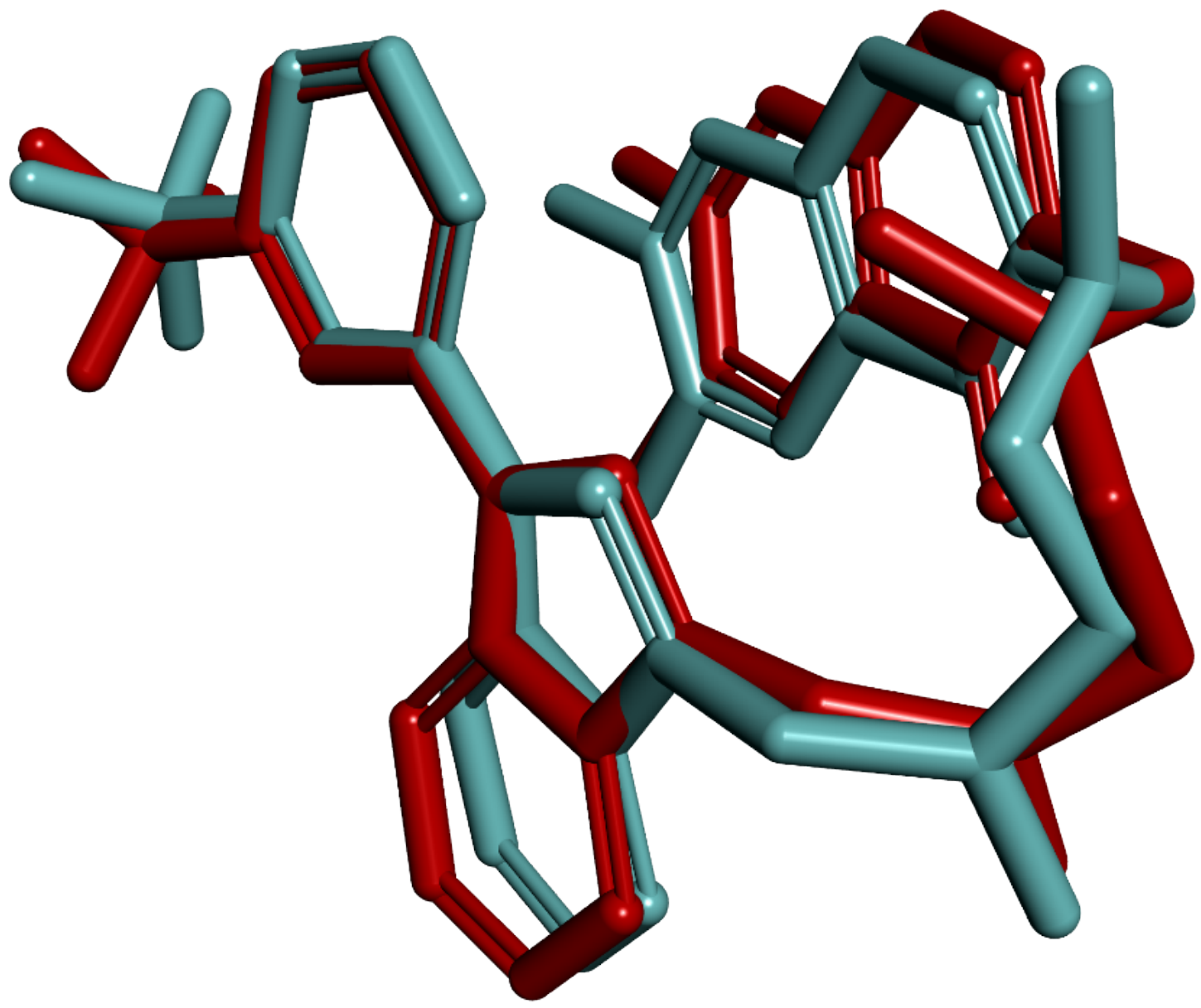

Fig. S1. Superimposition of redocked (red) and the co–crystalized ligand (light blue) in the protein 2AZ5 for the validation of docking protocol

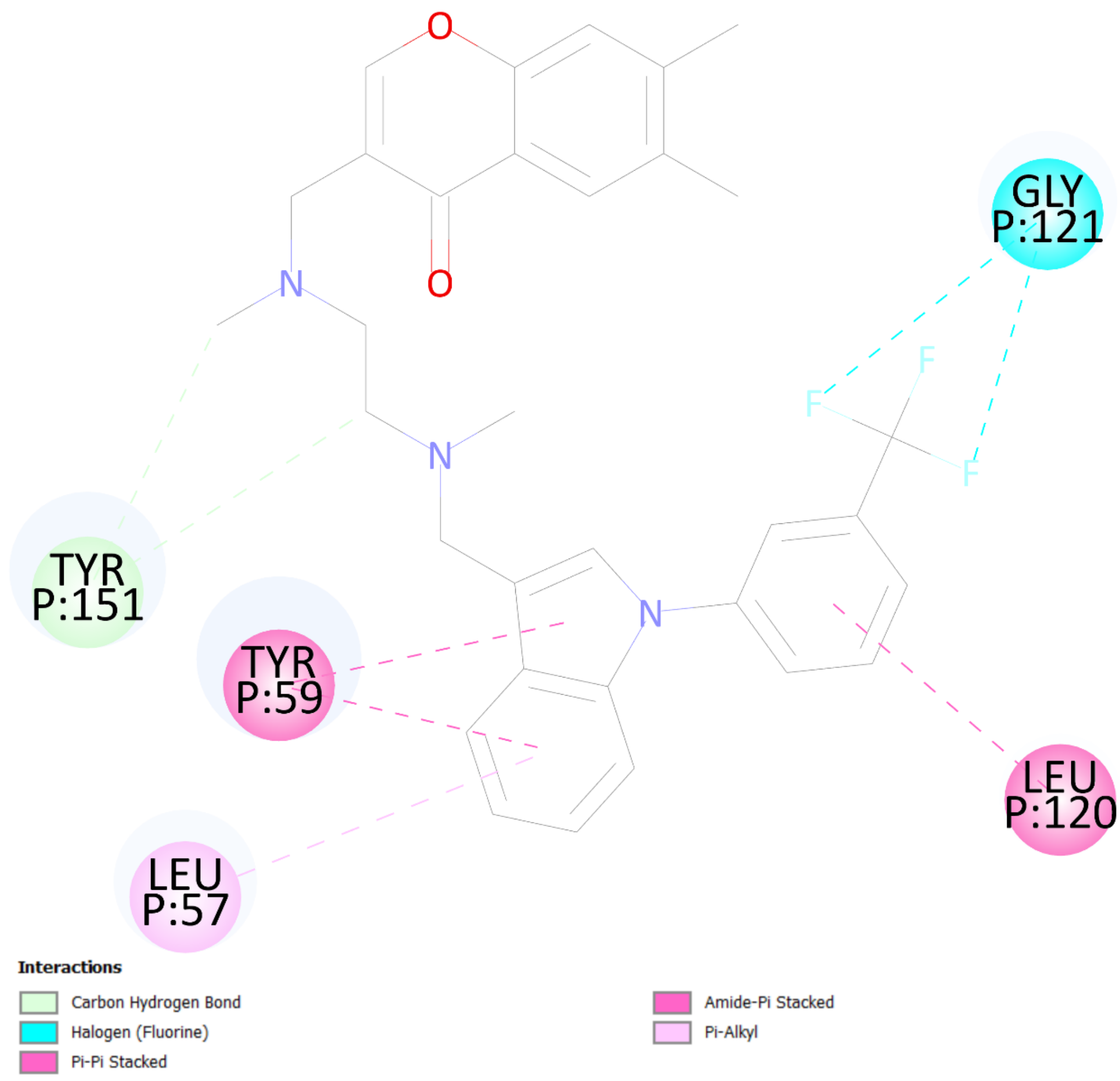

Fig. S2. 2D interaction between the co–crystalized ligand and protein TNF-α

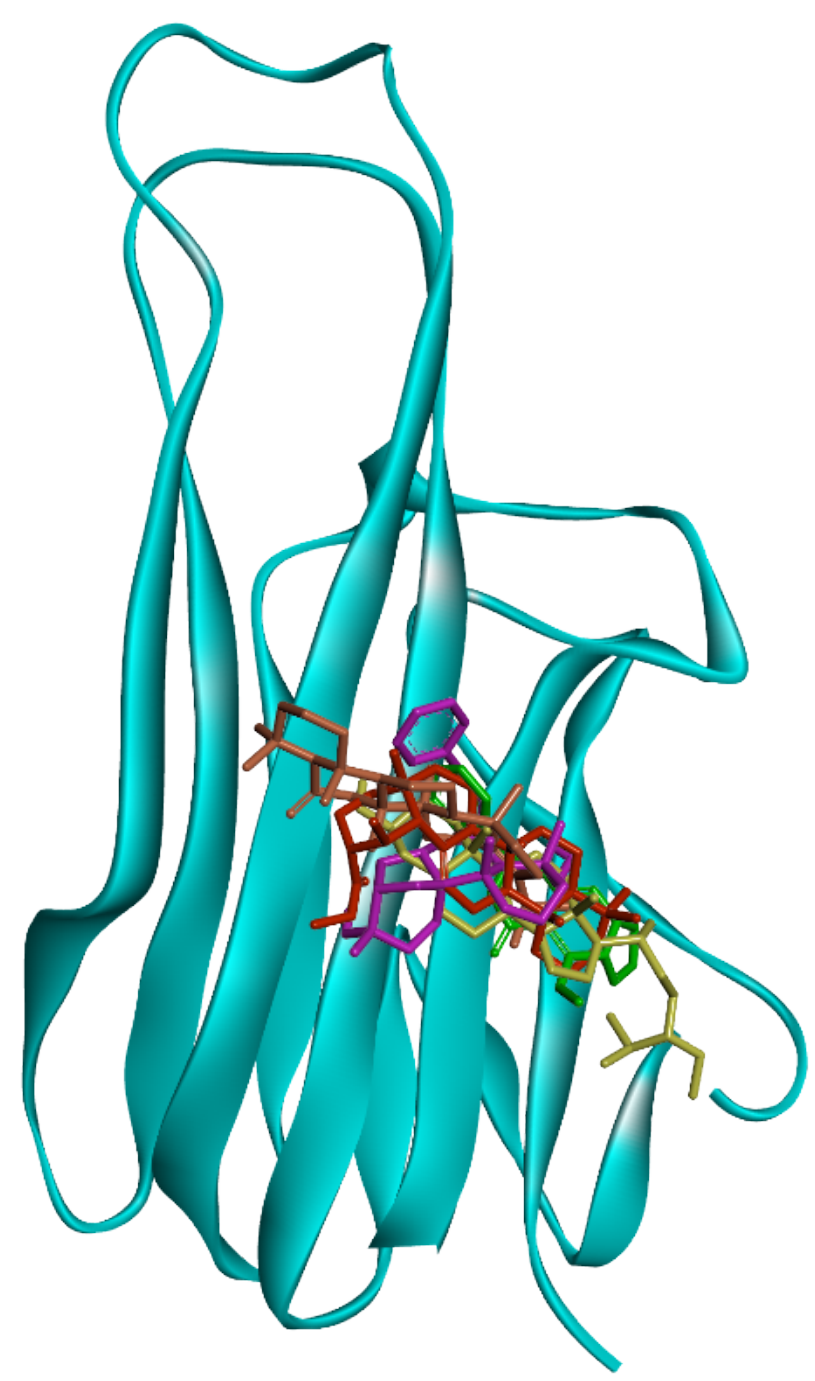

Fig. S3 Structural superimposition of the top-ranked docked compounds within the binding site of TNF-

α

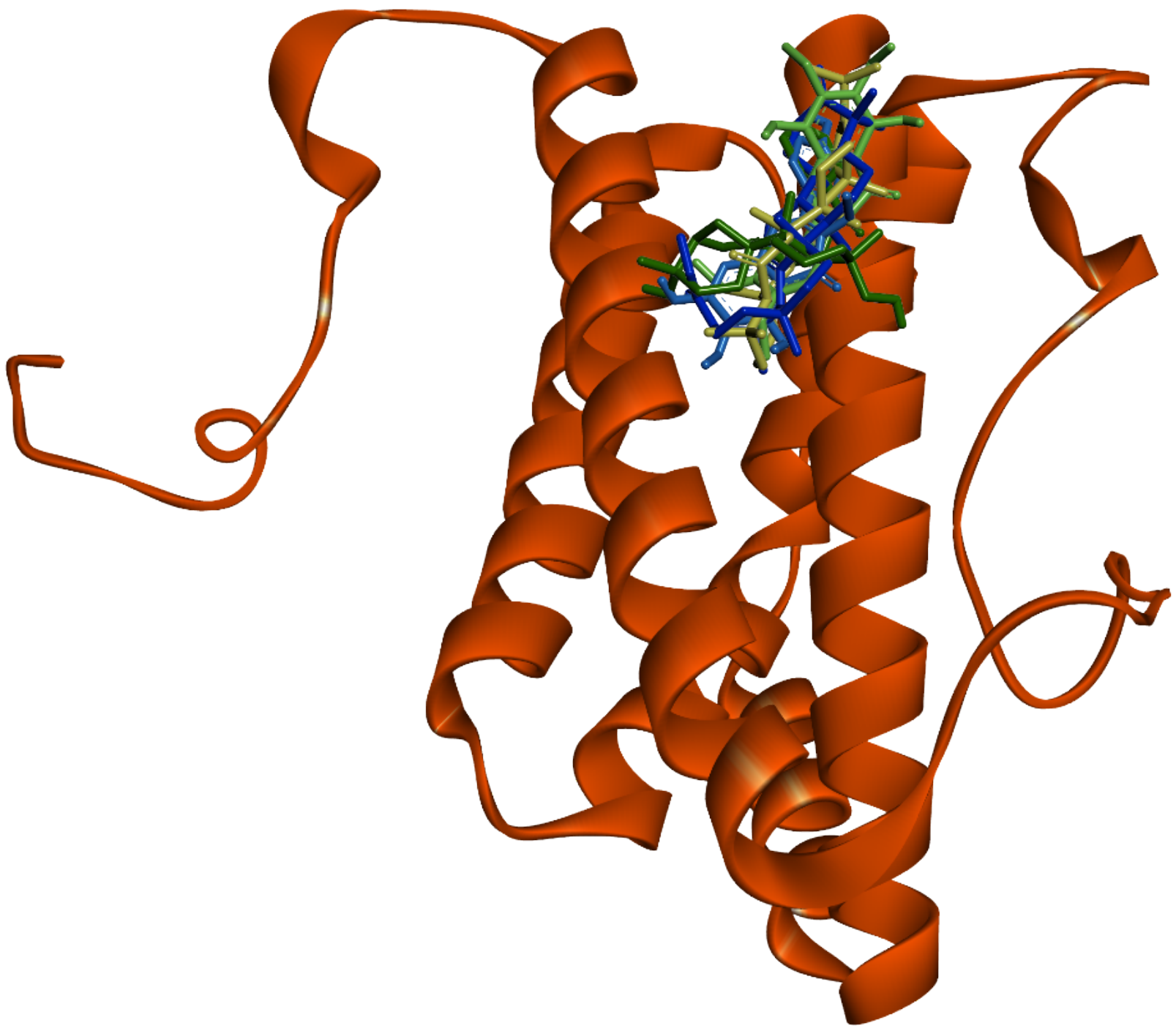

Fig. S4 Structural superimposition of the top-ranked docked compounds within the binding site of IL6

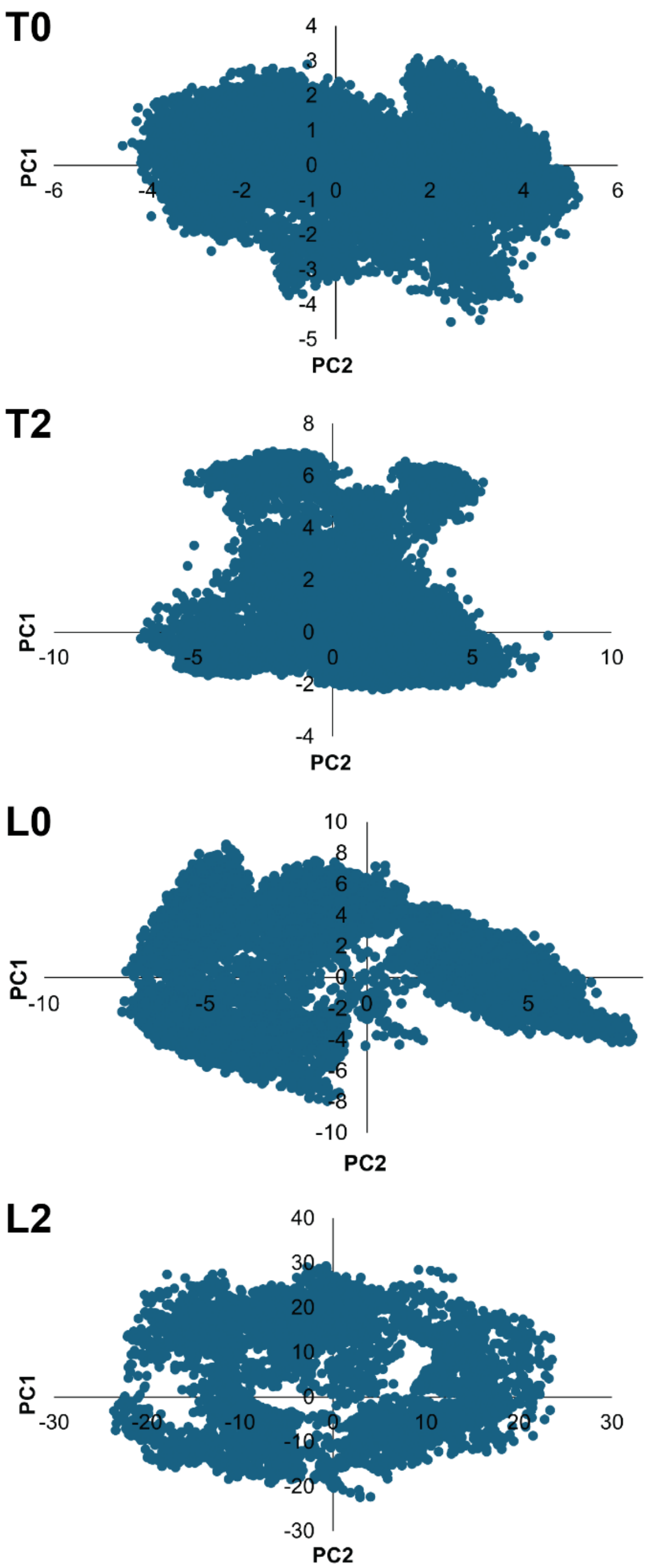

Fig. S5 PCA scatter plots of PC1 versus PC2 for TNF-α and IL-6 in the apoprotein and ligand-bound states. TNF-α systems include the apoprotein (T0), ASD21-bound form (T1), ASD68-bound form (T2),

and control-bound form (T3), whereas IL-6 systems comprise the apoprotein (L0), ASD21-bound form (L1), and ASD179-bound form (L2).
